# Unimon qubit

**DOI:** 10.1038/s41467-022-34614-w

**Published:** 2022-11-12

**Authors:** Eric Hyyppä, Suman Kundu, Chun Fai Chan, András Gunyhó, Juho Hotari, David Janzso, Kristinn Juliusson, Olavi Kiuru, Janne Kotilahti, Alessandro Landra, Wei Liu, Fabian Marxer, Akseli Mäkinen, Jean-Luc Orgiazzi, Mario Palma, Mykhailo Savytskyi, Francesca Tosto, Jani Tuorila, Vasilii Vadimov, Tianyi Li, Caspar Ockeloen-Korppi, Johannes Heinsoo, Kuan Yen Tan, Juha Hassel, Mikko Möttönen

**Affiliations:** 1 IQM, Keilaranta 19, 02150 Espoo, Finland; 2 QCD Labs, QTF Centre of Excellence, Department of Applied Physics, Aalto University, P.O. Box 13500, FIN-00076 Aalto, Finland; 3 VTT Technical Research Centre of Finland Ltd. & QTF Centre of Excellence, P.O. Box 1000, 02044 VTT Espoo, Finland

**Keywords:** Qubits, Quantum information, Superconducting devices

## Abstract

Superconducting qubits seem promising for useful quantum computers, but the currently wide-spread qubit designs and techniques do not yet provide high enough performance. Here, we introduce a superconducting-qubit type, the *unimon*, which combines the desired properties of increased anharmonicity, full insensitivity to dc charge noise, reduced sensitivity to flux noise, and a simple structure consisting only of a single Josephson junction in a resonator. In agreement with our quantum models, we measure the qubit frequency, *ω*_01_/(2*π*), and increased anharmonicity *α*/(2*π*) at the optimal operation point, yielding, for example, 99.9% and 99.8% fidelity for 13 ns single-qubit gates on two qubits with (*ω*_01_, *α*) = (4.49 GHz, 434 MHz) × 2*π* and (3.55 GHz, 744 MHz) × 2*π*, respectively. The energy relaxation seems to be dominated by dielectric losses. Thus, improvements of the design, materials, and gate time may promote the unimon to break the 99.99% fidelity target for efficient quantum error correction and possible useful quantum advantage with noisy systems.

## Abstract (web summary)

While transmon is the most widely used superconducting qubit, the search for alternative qubit designs with improved characteristic is ongoing. Hyyppä et al. demonstrate a novel superconducting qubit, the unimon, that combines high anharmonicity and protection against low-frequency charge noise and flux noise.

## Introduction

Even though quantum supremacy has already been reached with superconducting qubits in specific computational tasks^1,2^, the current quantum computers still suffer from errors owing to noise to the extent that their practical applications in areas such as physics simulations^3^, optimization^4^, machine learning^5^, and chemistry^6^ remain out of reach. In this so-called noisy intermediate-scale quantum (NISQ) era^7^, the complexity of the implementable quantum computations^8^ is mostly limited by errors in single- and two-qubit quantum gates. Crudely speaking, the process fidelity of implementing a *d*-deep *n*-qubit logic circuit with gate fidelity *F* is *F*^*dn*^. Thus, to succeed roughly half of the time in a 100-qubit circuit of depth five, one needs at least 99.9% gate fidelity. In practice, the number of qubits and especially the gate depth required for useful NISQ advantage is likely higher, leading to a fidelity target of 99.99% for all quantum gates, not yet demonstrated in any superconducting quantum computer.

The effect of gate errors can be reduced to some extent using error mitigation^9,10^ or in principle, completely using quantum error correction^11^. Surface codes^12,13^ are regarded as some of the most compelling error correction codes for superconducting qubits owing to the two-dimensional topology of the qubit register and their favorable fidelity threshold of roughly 99% which has been reached with superconducting transmon qubits already in 2014^14^ with following important steps reported in refs. 15, 16. Despite the recent major developments in implementing distance-2–5 surface codes on superconducting quantum processors^17–23^, the gate and readout fidelities of superconducting qubits need to be improved further, preferably above 99.99%, to enable efficient quantum error correction with a reasonable qubit count.

Currently, most of the superconducting multi-qubit processors utilize transmon qubits^1,17,24,25^ that can be reproducibly fabricated^24^ and have coherence times up to several hundred microseconds^26,27^, leading to record average gate fidelities of 99.98–99.99% for single-qubit gates^28,29^ and 99.8–99.9% for two-qubit gates^30,31^. The transmon was derived from the charge qubit^32^ by adding a shunt capacitor in parallel with a Josephson junction, with the result of exponentially suppressing the susceptibility of its transition frequency to charge noise. However, the large shunt capacitance results in a relatively low anharmonicity of 200–300 MHz corresponding to only 5% of the typical qubit frequency^33,34^. This limits the speed of quantum gates that can be implemented with transmons since leakage errors to the states beyond the computational subspace need to be suppressed^28,35^. Similarly, the low anharmonicity also limits the readout speed of transmon qubits and a high-power readout tone can even excite the transmon to unconfined states beyond the cosine potential^36^ (see Supplementary Note II in Supplementary Materials). A higher anharmonicity is preferred to speed up the qubit operations and to allow for higher fidelities limited by the finite coherence time.

Hence, it is desirable to find new superconducting qubit types that increase the anharmonicity–coherence-time product. Recently, major progress has been made in the development of fluxonium qubits, one of the most compelling alternatives to transmons thanks to their high anharmonicity and long relaxation and coherence times^37–39^ which recently enabled an average gate fidelity exceeding 99.99% for single-qubit gates^40^ and 99.7% for a two-qubit gate^38^. In a fluxonium qubit, a small Josephson junction is shunted by a superinductor implemented by an array of large Josephson junctions^37,39,41^, a granular aluminum wire^42^, a nanowire with a high kinetic inductance^43^, or a geometric superinductor^44^. The superinductor in the fluxonium ensures that the dephasing and relaxation rates arising from flux noise are reduced, in addition to which all the levels of a fluxonium are fully protected against dephasing arising from low-frequency charge noise. It is possible to add a large shunt capacitor into the fluxonium in order to create a so-called heavy fluxonium^39,45^, in which the transition matrix element between the ground state and the first excited state can be suppressed to enhance the relaxation time up to the millisecond regime^45^. However, special techniques are required to control, readout, and reset these high-coherence fluxonium qubits due to their low frequency and small transition matrix elements in the vicinity of the half flux quantum operation point^39^. Furthermore, these qubits do not achieve protection against both relaxation and dephasing due to flux noise at a single operation point. Parasitic capacitances in the superinductor may also provide a challenge for the reproducible fabrication of fluxonium qubits and result in parasitic modes.

By reducing the total inductance of the junction array in the fluxonium, it is possible to implement a plasmonium qubit^46^ operated at zero flux or a quarton qubit^47^ operated at the half-flux-quantum point, both of which have a small size and a high anharmonicity compared with the transmon and a sufficient protection against charge noise in comparison to current coherence times. On the other hand, an enhancement of the superinductance converts the fluxonium into a so-called quasicharge qubit^48^, the charge-basis eigenstates of which resemble those of the early charge qubits while retaining the protection against charge noise. Other qubits protected against some sources of relaxation and dephasing include the 0 − *π* qubit^49^, bifluxon^50^, and a qubit protected by two-Cooper-pair tunneling^51^. The 0 − *π* qubit is protected against both relaxation and dephasing arising from charge and flux noise thanks to its topological features, which unfortunately renders the qubit challenging to operate and its circuit relatively complicated and hence vulnerable to parasitic capacitance. Despite this great progress in fluxonium and protected qubits, they have still not shown broad superiority to the transmons. The race for the new improved mainstream superconducting qubit continues.

**Fig. 1:**
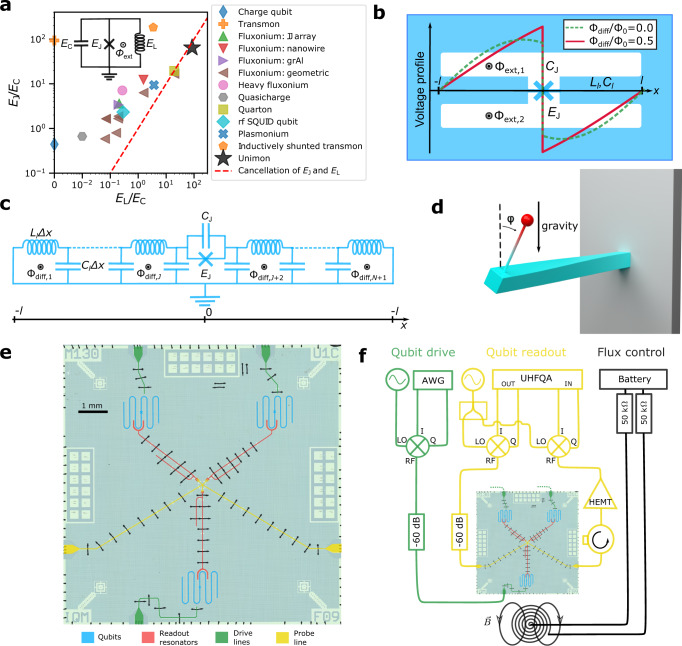
Unimon qubit and its measurement setup. **a** Superconducting-qubit types, described by the circuit in the inset, mapped by their energy scales: Josephson energy *E*_J_ and inductive energy *E*_L_ compared with the charging energy *E*_C_. Unimons lie near the red dashed line leading to the cancellation of the linear inductive energy by the quadratic contribution of the Josephson energy at half flux quantum Φ_0_/2. The black star denotes the unimons realized in this work and the other experimental data points are from refs. 32, 34, 37, 39, 42–44, 46–48, 80, 81. **b** Schematic unimon circuit consisting of a Josephson junction (*E*_J_, *C*_J_) in a grounded coplanar-waveguide (CPW) resonator of length 2*l* and an inductance and capacitance per unit length of *L*_*l*_ and *C*_*l*_, respectively. The voltage envelope functions of the qubit mode are also illustrated at external-flux biases Φ_diff_ = (Φ_ext,2_ − Φ_ext,1_)/2 = 0.0 (dashed line) and Φ_diff_ = Φ_0_/2 (solid line). **c** Distributed-element circuit model of the unimon, in which the CPW is modeled by *N* inductors *L*_*l*_Δ*x* and capacitors *C*_*l*_Δ*x*, where Δ*x* = 2*l*/*N*. **d** Schematic illustration of a mechanical inverted pendulum system, the Hamiltonian of which is identical to that of the lumped-element unimon circuit in (**a**). In this analogy, the gravitational potential energy corresponds to the Josephson potential, the harmonic potential energy of the twisting beam corresponds to the inductive energy, the moment of inertia corresponds to the capacitance of the unimon, and the angle of the zero twist position corresponds to the flux bias Φ_diff_. **e** False-color microscope image of a silicon chip containing three unimon qubits (blue) together with their readout resonators (red), drive lines (green), and a joint probe line (yellow). **f** Simplified experimental setup used to measure the unimon qubits at 10 mK (see Supplementary Methods IV for details).

**Fig. 2:**
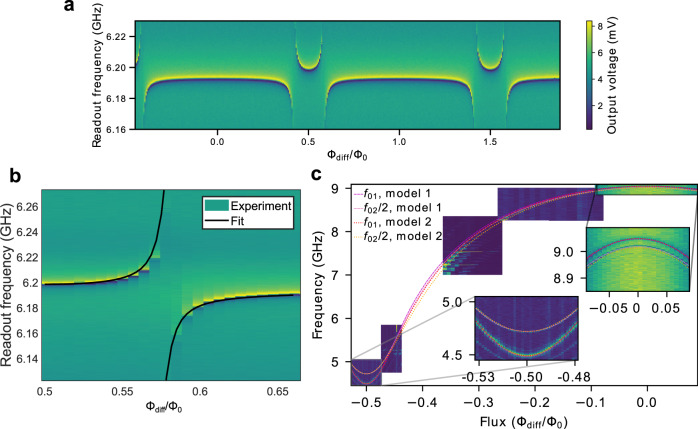
Resonator and qubit B spectroscopies. **a** Magnitude of the readout signal voltage transmitted through the probe line as a function of the signal frequency and the flux bias Φ_diff_ of the unimon. **b** Magnification at an avoided crossings of **a**, where a unimon and its readout resonator are close to resonance, together with a fit (solid black line) used to estimate the coupling capacitance *C*_g_ between the qubit and the resonator. The fit is based on diagonalizing Eq (5) in “Methods”. **c** Magnitude of the readout signal at a properly chosen readout frequency as a function of the flux bias Φ_diff_ and qubit excitation frequency, revealing the spectral lines of the unimon together with global fits to the theoretical model 1 and 2 (“Methods”). The insets show magnifications at the flux sweet spots, highlighting that at half flux quantum, the unimon frequency is minimized whereas its anharmonicity is maximized.

**Fig. 3:**
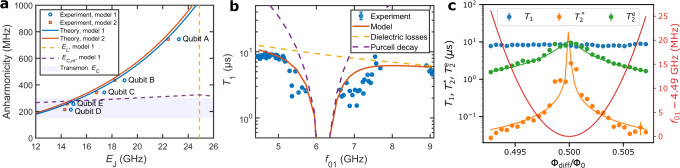
Measurement of key qubit properties. **a** Predicted (solid lines) and measured anharmonicities of five unimon qubits (markers) at flux bias Φ_diff_ = Φ_0_/2 as functions of the Josephson energy *E*_J_ estimated from data similar to Fig. 2c using the models 1 (blue color) and 2 (orange color) presented in “Methods”. The dashed yellow line highlights the *E*_J_ that perfectly cancels the linear inductive energy in model 1. The capacitive qubit energy *E*_*C*,*m*_ in model 1 follows the dashed purple line, whereas the purple shading visualizes typical transmon anharmonicities. **b** Experimentally measured mean energy relaxation time *T*_1_ of qubit B (blue circles) as a function of the qubit frequency *f*_01_ together with a model (solid line) taking into account dielectric losses (dashed yellow line) and the Purcell decay (dashed purple line). The error bars represent the standard error of the mean obtained from 6 to 30 repetitions of single *T*_1_ measurements conducted at each frequency. **c** Relaxation time *T*_1_, Ramsey coherence time ${T}_{2}^{*}$, and echo coherence time ${T}_{2}^{{{{{{{{\rm{e}}}}}}}}}$ of qubit B as functions of the flux bias Φ_diff_ in the vicinity of Φ_diff_ = Φ_0_/2. The error bars of *T*_1_ and ${T}_{2}^{*}$ represent the standard error of the mean based on 8 repeated experiments, whereas the error bars of ${T}_{2}^{{{{{{{{\rm{e}}}}}}}}}$ represent the standard error derived from the standard deviations of the fitted dephasing rates ${{{\Gamma }}}_{\varphi,{{\Phi }}}^{{{{{{{{\rm{e}}}}}}}}}$ and ${{{\Gamma }}}_{\varphi,0}^{{{{{{{{\rm{e}}}}}}}}}$ by applying Eq. (12). The green and orange lines illustrate fits to ${T}_{2}^{{{{{{{{\rm{e}}}}}}}}}$ and ${T}_{2}^{*}$ data based on models regarding the dephasing rate as a linear function of ∂*f*_01_/∂Φ_diff_ (“Methods”). The red line shows the detuning of the qubit frequency from its minimum value 4.49 GHz.

**Fig. 4:**
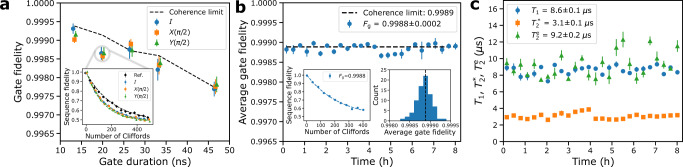
Implementation of fast and stable single-qubit gates for qubit B. **a** Average gate fidelity as a function of the gate duration for gates in the set {*I*, *X*(*π*/2), *Y*(*π*/2)}. The error bars represent the standard error of the mean based on six interleaved randomized benchmarking experiments. The dashed black line shows the coherence limit computed based on the mean values of the energy relaxation time *T*_1_ and the echo coherence time ${T}_{2}^{{{{{{{{\rm{e}}}}}}}}}$. The inset shows the results of an exemplary interleaved randomized benchmarking experiment corresponding to a gate duration of 20 ns. **b** Average gate fidelity *F*_g_ measured with randomized benchmarking as a function of time lapsed from the initial gate and parameter calibration. The error bars represent the standard error of the mean from three consecutive randomized benchmarking experiments. The dashed black line shows the coherence limit that has been computed using the mean values of *T*_1_ and ${T}_{2}^{{{{{{{{\rm{e}}}}}}}}}$ measured interleaved with the gate fidelity. The left inset illustrates the decay of the sequence fidelity as a function of its length in an exemplary randomized-benchmarking experiment. The right inset shows the histogram of the gate fidelities obtained over the measurement period of eight hours. **c** Stability of *T*_1_, ${T}_{2}^{{{{{{{{\rm{e}}}}}}}}}$, and the Ramsey coherence time ${T}_{2}^{*}$ over a period of eight hours. The qubit parameters were calibrated only once before the characterization measurements. The error bars represent the standard error of the mean from three consecutive measurements.

In this work, we introduce and demonstrate a novel superconducting qubit, *the unimon*, that consists of a single Josephson junction shunted by a linear inductor and a capacitor in a largely unexplored parameter regime where the inductive energy is mostly cancelled by the Josephson energy leading to high anharmonicity while being fully resilient against low-frequency charge noise and partially protected from flux noise (Fig. 1). We measure the unimon frequency and anharmonicity in a broad range of flux biases and find a very good agreement with first-principles models (Fig. 2), even for five different qubits (Fig. 3a). According to our experimental data, the energy relaxation time seems to be limited by dielectric losses (Fig. 3b), and the coherence time can be protected from flux noise at a flux-insensitive sweet spot (Fig. 3c). Importantly, we observe that the single-qubit gate fidelity progressively increases with decreasing gate duration, and is stable for hours at 99.9% for a 13 ns gate duration (Fig. 4).

## Results

### Unimon qubit

In practice, we implement the unimon in a simple superconducting circuit by integrating a single Josephson junction into the center conductor of a superconducting coplanar-waveguide (CPW) resonator grounded at both ends (Fig. 1b). There are no charge islands in the circuit, and hence the junction is inductively shunted. In addition to the very recent fluxonium qubit utilizing a geometric superinductance^44^, the unimon is the only superconducting qubit with the Josephson junction shunted by a geometric inductance that provides complete protection against low-frequency charge noise. Due to the non-linearity of the Josephson junction, the normal modes of the resonator with a non-zero current across the junction are converted into anharmonic oscillators that can be used as qubits. In this work, we use the lowest anharmonic mode as the qubit since it has the highest anharmonicity.

The frequency of each anharmonic mode can be controlled by applying external fluxes Φ_ext,1_ and Φ_ext,2_ through the two superconducting loops of the resonator structure as illustrated in Fig. 1b. The unimon is partially protected against flux noise thanks to its gradiometric structure, which signifies that the superconducting phase across the Josephson junction is dependent on the half difference of the applied external magnetic fluxes Φ_diff_ = (Φ_ext,2_ − Φ_ext,1_)/2. Interestingly, the anharmonicity of the unimon is maximized at a flux-insensitive sweet spot, at which the qubit frequency is unaffected by the external flux difference to the first order. This optimal operation point is obtained at Φ_diff_ = Φ_0_/2 modulo integer flux quanta Φ_0_ = *h*/(2*e*) ≈ 2.067 × 10^−15^ Wb, where *h* is the Planck constant and *e* is the elementary charge.

**Fig. 5:**
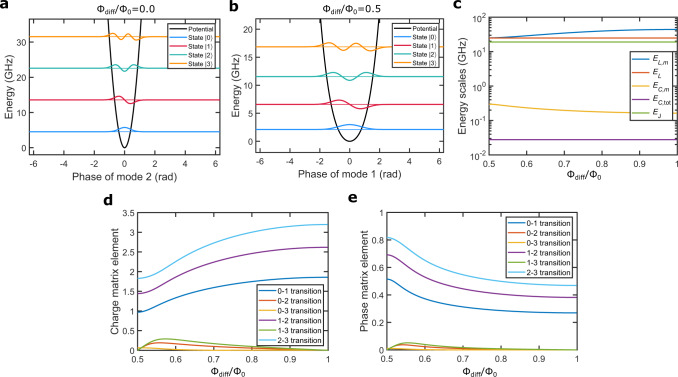
Potential-energy function, energy scales, and matrix elements of qubit B based on model 1. **a**, **b** Potential energy of the unimon based on equation (4) in Methods as a function of the phase variable *φ*_*m*_ of mode *m* together with the four lowest eigenenergies and corresponding phase-basis wave functions at flux biases of Φ_diff_ = 0 (**a**) and Φ_diff_ = Φ_0_/2 (**b**). Note that the second mode of the circuit is used as the qubit at Φ_diff_ = 0 and the first mode at Φ_diff_ = Φ_0_/2. **c** Energy scales *E*_*C*,*m*_, *E*_*L*,*m*_, *E*_*L*_, and *E*_J_ of the qubit as functions of Φ_diff_. Here, *E*_*C*,*m*_ and *E*_*L*,*m*_ are the capacitive and inductive energies of the qubit mode, *E*_*L*_ is the inductive energy corresponding to a dc current in the center conductor of the qubit, and *E*_J_ is the Josephson energy. We also show an effective charging energy *E*_*C*,tot_ = *e*^2^/(4*C*_*l*_*l*) computed based on the total capacitance 2*C*_*l*_*l* of the transmission line of the unimon. **d** Off-diagonal matrix elements $|\left\langle i\right|{\hat{n}}_{m}\left|j\right\rangle|$ of the Cooper pair number operator ${\hat{n}}_{m}$ for the four lowest-energy states of the qubit mode *m* as functions of Φ_diff_. **e**, As **d** but for the phase operator ${\hat{\varphi }}_{m}$. In all panels, the results have been obtained by using the theoretical model 1 in “Methods” and the measured parameter values of qubit B reported in Table 1.

Using the distributed-element circuit model shown in Fig. 1c, the effective Hamiltonian of the qubit mode *m* can be written (model 1 in “Methods”) as

1 $${\hat{H}}_{m}=4{E}_{C,m}({\varphi }_{0}){\hat{n}}_{m}^{2}+\frac{1}{2}{E}_{L,m}({\varphi }_{0}){\hat{\varphi }}_{m}^{2}+{E}_{L}{\hat{\varphi }}_{m}\left(\frac{2\pi {{{\Phi }}}_{{{{{{{{\rm{diff}}}}}}}}}}{{{{\Phi }}}_{0}}-{\varphi }_{0}\right)-{E}_{{{{{{{{\rm{J}}}}}}}}}\cos ({\hat{\varphi }}_{m}-{\varphi }_{0}),$$

where *φ*_0_ is the Josephson phase of a dc current across the junction, *E*_*C*,*m*_(*φ*_0_) is the capacitive energy of the qubit mode, *E*_*L*,*m*_(*φ*_0_) is the inductive energy of the qubit mode, *E*_*L*_ is the inductive energy of the dc current, *E*_J_ is the Josephson energy, and ${\hat{n}}_{m}$ and ${\hat{\varphi }}_{m}$ are the Cooper pair number and phase operators corresponding to the qubit mode *m* and satisfying $[{\hat{\varphi }}_{m},\,{\hat{n}}_{m}]={{{{{{{\rm{i}}}}}}}}$ with i being the imaginary unit. Note that *φ*_0_ is treated as a classical variable depending on the flux bias Φ_diff_ according to a transcendental equation such that 2*π*Φ_diff_/Φ_0_ − *φ*_0_ is periodic in Φ_diff_. (See Fig. 5 for solutions of Eq. (1).)

At the sweet spot Φ_diff_ = Φ_0_/2, the dc phase equals *φ*_0_ = *π* and the Hamiltonian of the unimon reduces to

2 $${\hat{H}}_{m}=4{E}_{C,m}(\pi ){\hat{n}}_{m}^{2}+\frac{1}{2}{E}_{L,m}(\pi ){\hat{\varphi }}_{m}^{2}+{E}_{{{{{{{{\rm{J}}}}}}}}}\cos ({\hat{\varphi }}_{m}),$$

where we assume that *E*_J_ ≤ *E*_*L*_. Strikingly, this Hamiltonian is exactly analogous to a simple mechanical system visualized in Fig. 1d, in which an inverted pendulum is attached to a twisting beam. In this analogy, the gravitational potential energy of the pendulum corresponds to the cosine-shaped Josephson potential, the harmonic potential energy associated with the twisting of the beam corresponds to the inductive energy of the unimon, and the moment of inertia of the pendulum is analogous to the capacitance in the unimon. Furthermore, the twist angle *φ* is analogous to the superconducting phase difference ${\hat{\varphi }}_{m}$ of the qubit mode across the Josephson junction. This mechanical analog provides great intuition to the physics of the unimon.

In this work, we employ the parameter regime *E*_J_ ≲ *E*_*L*,*m*_(*π*) ≈ *E*_*L*_ to provide a large anharmonicity without any superinductors. As a result, it is instructive to use the Taylor expansion of the cosine and write the sweet-spot Hamiltonian of the unimon in Eq. (2) as

3 $${\hat{H}}_{m}=4{E}_{C,m}(\pi ){\hat{n}}_{m}^{2}+\frac{{E}_{L,m}(\pi )-{E}_{{{{{{{{\rm{J}}}}}}}}}}{2}{\hat{\varphi }}_{m}^{2}+\frac{{E}_{{{{{{{{\rm{J}}}}}}}}}}{24}{\hat{\varphi }}_{m}^{4}+{{{{{{{\mathcal{O}}}}}}}}({\hat{\varphi }}_{m}^{6}).$$

The quadratic term proportional to (*E*_*L*,*m*_(*π*) − *E*_J_) is mostly cancelled in the unimon regime, which emphasizes the high-order terms in the potential energy and hence increases the anharmonicity of the qubit. This cancellation bears resemblance to the quarton qubit^47^ with the distinctive difference that the quadratic inductive energy of a quarton qubit is only an approximation for the actual potential energy function of a short Josephson junction array, as a result of which the quarton circuit is not fully protected against low-frequency charge noise unlike the unimon.

To experimentally demonstrate the unimon qubit, we design and fabricate samples, each of which consists of three unimon qubits as illustrated in Fig. 1e. We use niobium as the superconducting material apart from the Josephson junctions, in which the superconducting leads are fabricated using aluminum (see Sample Fabrication in Methods). The CPW structure of the unimon is designed for characteristic impedance *Z* = 100 Ω to reduce the total capacitance of the unimon in comparison to a standard 50 Ω resonator. Each qubit is capacitively coupled to an individual drive line that enables single-qubit rotations in a similar manner as for conventional transmon qubits by applying attenuated microwave pulses along the drive line as illustrated in the simplified schematic of the experimental setup in Fig. 1f (see also Supplementary Methods IV for a more detailed illustration of the setup). All experiments are carried out at 10 mK base temperature of a pulse-tube-cooled dilution refrigerator. Furthermore, each qubit is capacitively coupled to a readout resonator using a U-shaped capacitor in order to enable dispersive qubit state measurements^52,53^ similar to those conventionally used with transmon qubits^20^. The frequency of the qubits is tuned by applying a current through an external coil attached to the sample holder such that one flux quantum Φ_0_ approximately corresponds to 10 *μ*A.

### Experimental results on unimons

We experimentally study five unimon qubits, A–E, on two different chips. In all of the qubits, the geometry of the CPW resonator is similar, but the qubits have different Josephson energies *E*_J_ corresponding to different amounts of cancellation ∝ (*E*_*L*,*m*_(*π*) − *E*_J_) of the quadratic potential energy terms. Furthermore, the coupling capacitance between a qubit and its readout resonator has been designed to be different on the two chips. We present the main measured properties for all of the five qubits in Tables 1 and 2. Design targets of the parameter values are provided in Supplementary Table 2 in Supplementary Methods V. The results discussed below are obtained from qubit B unless otherwise stated.

**Table 1:** Characteristic parameter values for the five measured unimon qubits

Qubit	2*l*	*L*_*l*_	*C*_*l*_	*E*_J_/*h*	*E*_*L*,*m*_/*h*	*E*_*C*,*m*_/*h*	*ω*_01_/(2*π*)	*α*/(2*π*)	*x*_g_	*C*_g_	∣*g*_01_∣/(2*π*)	*χ*/(2*π*)	*f*_r_	*κ*/(2*π*)
	(mm)	(μH/m)	(pF/m)	(GHz)	(GHz)	(GHz)	(GHz)	(MHz)	(mm)	(fF)	(MHz)	(MHz)	(GHz)	(MHz)
A	8.0	0.821^*^	87.1^*^	23.3	24.9	0.318	3.547	744	0.596	9	53.5	0.74	5.826	0.43
B	8.0	0.821	87.1	19.0	25.2	0.297	4.488	434	0.596	10	70.0	1.2	6.198	1.24
C	8.0	0.821^*^	87.1^*^	17.4	25.3	0.290	4.781	343	0.596	12.5	79.7	9.20	5.522	9.2
D	8.0	0.821^*^	87.1^*^	14.8	25.7	0.278	5.257	214	0.596	12.5	85.7	20.2	5.699	10.0
E	8.0	0.821^*^	87.1^*^	15.0	25.7	0.279	5.224	257	0.596	12.5	92.3	4.1	6.156	1.8

For each of the characterized qubits, the data includes the total length 2*l* of the center conductor in the qubit, the fitted inductance *L*_*l*_ and capacitance *C*_*l*_ per unit length of the center conductor, the fitted Josephson energy *E*_J_, the inductive and capacitive energies *E*_*L,m*_ and *E*_*C,m*_, the measured qubit frequency *ω*_01_/(2π), the measured anharmonicity *α*/(2π), the location *x*_g_ of the unimon–resonator coupling point with respect to the junction, the measured coupling strength ∣*g*_01_∣/(2π) between the qubit and its readout resonator providing the corresponding coupling capacitance *C*_g_, the measured dispersive shift *χ*/(2π), the measured frequency of the readout resonator *f*_r_, and the measured linewidth of the readout resonator *κ*/(2π). All the fitted values were estimated using theory model 1 (“Methods”). The values of the flux-dependent quantities *E*_*L,m*_, *E*_*C,m*_, *ω*_01_/(2π), α/(2π), *g*_01_/(2π), *χ*/(2π), and *f*_r_ are reported at Φ_diff_ = Φ_0_/2. The asterisks denote the fact that the inductance *L*_*l*_ and capacitance *C*_*l*_ per unit length are estimated by fitting the theoretical model to the spectrum of qubit B and that equal values are used for the other qubits due to an identical cross section of the co-planar waveguide in all qubits. The inductive and capacitive energies *E*_*L,m*_ and *E*_*C,m*_ are computed using the theoretical model 1 (“Methods”) which is also used to obtain the measured values of *E*_*J*_, *L*_*l*_*,* and *C*_*l*_.

**Table 2:** Results of coherence characterization and randomized-benchmarking (RB) experiments for the five unimon qubits

	Φ_diff_ = Φ_0_/2					
Qubit	*T*_1_	${T}_{2}^{*}$	${T}_{2}^{{{{{{{{\rm{e}}}}}}}}}$	${A}_{{{{\Phi }}}_{{{{{{{{\rm{diff}}}}}}}}}}$	RB fidelity	Gate duration
	(μs)	(μs)	(μs)	(μΦ_0_)	(%)	(ns)
A	7.2	1.9	6.8	6.1	99.81	13.3
B	8.6	3.1	9.2	15.0	99.90	13.3
C	5.8	2.3	9.3	11.2	99.54	40
D	3.9	2.3	7.0	11.1	99.63	20
E	5.6	2.5	11.4	14.3	99.86	13.3

The measured energy relaxation time *T*_1_, the measured Ramsey coherence time ${T}_{2}^{*}$, the measured echo coherence time ${T}_{2}^{{{{{\rm{e}}}}}}$, the flux noise density parameter ${A}_{{{{\Phi }}}_{{{{{{{{\rm{diff}}}}}}}}}}$ at 1 Hz estimated from the echo coherence time, the average fidelity per microwave gate from standard RB experiments, and the gate duration used in the RB experiments. All of the values are measured in the immediate vicinity of the sweet spot Φ_diff_ = Φ_0_/2.

In Fig. 2a, we show the microwave response of the readout resonator as a function of the flux bias Φ_diff_ through the unimon loops. We observe that the frequency of the readout resonator changes periodically, as expected, since a change of flux by a flux quantum has no observable effect on the full circuit Hamiltonian in Eq. (1). Furthermore, the frequency of the readout resonator exhibits an avoided crossing where the first transition frequency of the bare qubit *f*_01_ = *ω*_01_/(2*π*) crosses the bare resonator frequency. By fitting our theoretical model of the coupled unimon-resonator system (see Methods and Supplementary Methods II) to the experimental data of the avoided crossing shown in Fig. 2b, we estimate that the coupling capacitance between the qubit and the readout resonator is *C*_g_ = 10.0 fF in good agreement with the design value of 10.4 fF obtained from our classical electromagnetic simulations.

**Fig. 6:**
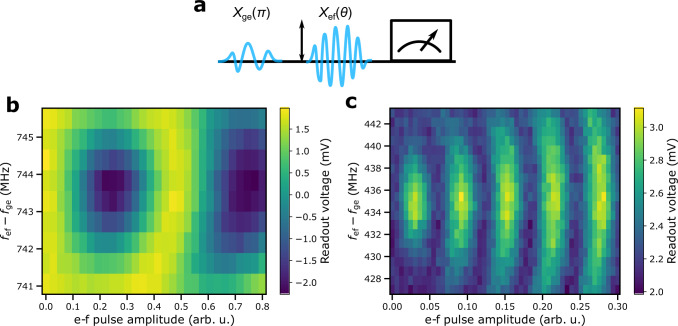
Estimation of qubit anharmonicity using Rabi oscillations. **a** Measurement sequence of an ef-Rabi experiment. In the experiment, the qubit is first prepared in the excited state with a *π*-pulse at the frequency *f*_ge_ matching with the qubit frequency *f*_01_ followed by another pulse with a fixed duration but a varying frequency *f*_ef_ and a varying amplitude. After applying the pulses, the output readout voltage is measured. Output readout voltage as a function of the amplitude of the ef-pulse and the frequency difference *f*_ef_ − *f*_ge_ in an ef-Rabi experiment conducted for qubits A (**b**) and B (**c**). The resulting observed Rabi oscillations between the first and the second excited states of the unimon comfirm that the anharmonicities *α* of qubits A and B are approximately 744 MHz and 434 MHz, respectively, as measured using a two-tone qubit spectroscopy such as that shown in Fig. 2c.

Figure 2c shows the results of a two-tone experiment to map the qubit frequency spectrum (Methods). We observe that the single-photon transition between the ground state $\left|0\right\rangle$ and the first excited state $\left|1\right\rangle$ has a minimum frequency of *f*_01_ = 4.488 GHz at Φ_diff_/Φ_0_ = − 0.5 and a maximum frequency of *f*_01_ = 9.05 GHz at Φ_diff_ = 0. The two-photon transition $\left|0\right\rangle \leftrightarrow \left|2\right\rangle$ is also clearly visible, which allows us to verify that the anharmonicity *α*/(2*π*) = *f*_12_ − *f*_01_ of the qubit is enhanced at the sweet spot Φ_diff_/Φ_0_ = −0.5 to *α*/(2*π*) = 434 MHz. (See Fig. 6 for an alternative agreeing way to measure the anharmonicity.)

Figure 2c presents fits to the experimental transition frequencies *f*_01_ and *f*_02_/2 based on two theoretical models of the circuit Hamiltonian, the first of which corresponds to Eq. (1) (model 1 in “Methods”) and the second of which is based on a path integral approach that does not require the dc phase *φ*_0_ to be treated as a classical variable (model 2 in Methods). The fits agree very well with the experimental transition frequencies, especially near the sweet spots Φ_diff_ = 0 and Φ_diff_/Φ_0_ = −0.5. Importantly, this good agreement with the models and the qubit frequency and anharmonicity is obtained with only three fitting parameters in a broad range of flux biases, and hence confirms our interpretation of the unimon physics (Fig. 1d) and justifies the use of the models for reliable predictions of promising parameter regimes. According to the fits of model 1 (model 2), the capacitance and inductance per unit length of the unimon have a value of *C*_*l*_ = 87.1 pF/m (*C*_*l*_ = 79.8 pF/m) and *L*_*l*_ = 0.821 μH/m (*L*_*l*_ = 0.893 μH/m), respectively, in good agreement with the design values of *C*_*l*_ = 83 pF/m and *L*_*l*_ = 0.83 μH/m.

The measured sweet-spot anharmonicities of the five qubits are shown in Fig. 3a as functions of the Josephson energy *E*_J_ that is estimated by fitting the models 1 and 2 to the qubit spectroscopy data as in Fig. 2c. The measured anharmonicities are slightly lower, but very close to the values predicted by the two theoretical models. The qubits A and B exhibit the highest anharmonicities of *α*/(2*π*) = 744 MHz and 434 MHz, respectively, as a result of the largest cancellation between the inductive energy *E*_*L*,*m*_ and the Josephson energy *E*_J_. Importantly, the anharmonicity of the qubits A and B is significantly higher than that of typical transmon qubits, 200–300 MHz^34^. Furthermore, the measured anharmonicities greatly exceed the capacitive energy *E*_*C*,*m*_ of the qubit mode unlike for transmons.

**Fig. 7:**
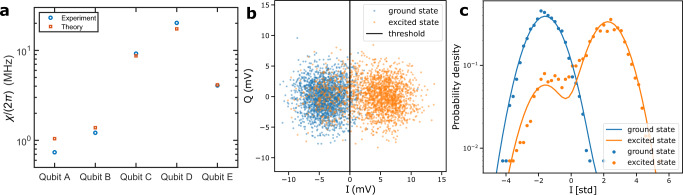
Dispersive shift and single-shot qubit readout. **a** Measured dispersive shift *χ* for different unimon qubits at a flux bias of Φ_diff_ = Φ_0_/2 (blue circles) together with theoretical predictions (orange squares) computed using Eq. (8) in “Methods” based on the measured qubit frequency, measured anharmonicity, and the fitted coupling capacitance *C*_g_, all of which are reported in Table 1. **b** Readout voltages in the *IQ* plane for a single-shot readout experiment conducted for qubit E. The qubit is prepared either to the ground state (blue dots) or to the excited state (orange dots) followed by a single-shot state measurement implemented using a readout pulse with a duration of 1.6 μs. The state preparation was repeated 2000 times for both the ground state and the excited state. The solid black line illustrates the optimal single-shot classification boundary corresponding to a readout fidelity of 89.0%. The classification errors are 5.0 and 17.0% when preparing the qubit to the ground state and to the excited state, respectively. **c** Probability density distributions for the single-shot voltage corresponding to the qubit prepared to the ground state (blue dots) or to the excited state (orange dots). The single-shot voltages have been projected along a line perpendicular to the classification boundary. The solid lines denote fits to the measured probability densities based on a model involving a sum of two Gaussian functions.

**Fig. 8:**
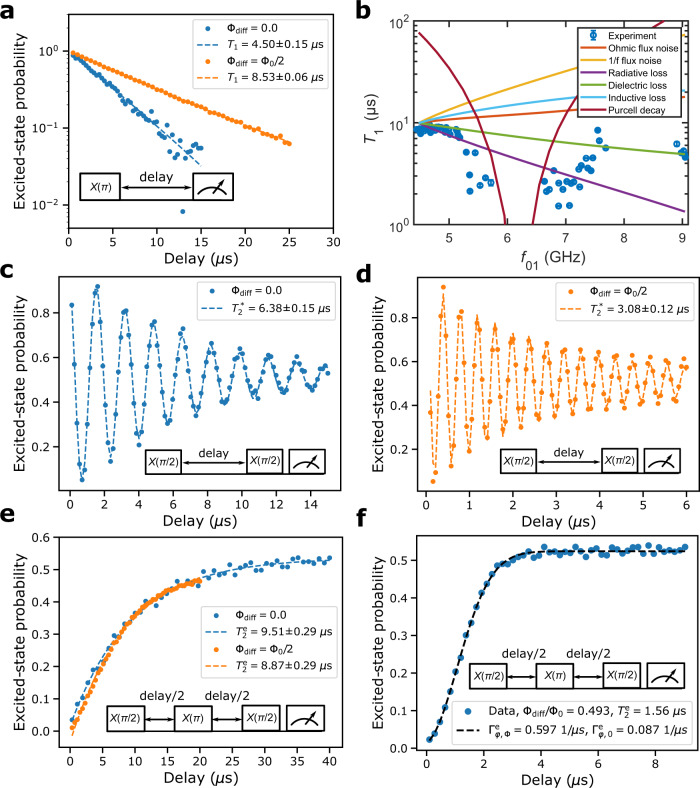
Energy relaxation and coherence for qubit B. **a** Excited-state probability as a function of the delay time between the state preparation and readout (see inset) at flux biases Φ_diff_ = 0 (blue color) and Φ_diff_ = Φ_0_/2 (orange color). The exponential fits (dashed lines) yield energy relaxation times *T*_1_ = 4.5 ± 0.2 μs and *T*_1_ = 8.5 ± 0.1 μs at Φ_diff_ = 0 and Φ_0_/2, respectively. **b** Measured mean *T*_1_ as a function of the qubit frequency *f*_01_ (blue circles) and its theoretical predictions from different indicated loss mechanisms that are scaled to coincide with the experimental data at Φ_diff_ = Φ_0_/2 excluding Purcell decay. The error bars provide the standard error of the mean based on 6–30 repeated *T*_1_ measurements. **c** Measured (markers) and fitted (dashed line) excited-state probability as a function of the delay time between the *X*(*π*/2) pulses of a Ramsey measurement (see inset) conducted at Φ_diff_ = 0 using a detuning of 0.6 MHz between the drive and qubit frequencies. **d** As **c** but for Φ_diff_ = Φ_0_/2 and a detuning of 2.5 MHz. **e** Measured excited-state probability (markers) as a function of the delay time between the *X*(*π*/2) pulses in a *T*_2_ echo measurement (see inset) conducted at the sweet spots Φ_diff_ = 0 (blue color) and Φ_diff_ = Φ_0_/2 (orange color). The dash lines provide exponential fits to the data. **f** As **e** but for Φ_diff_/Φ_0_ = 0.493. Here, the dashed line shows a model corresponding to a product of a Gaussian and an exponential with decay rates ${{{\Gamma }}}_{\varphi,{{\Phi }}}^{{{{{{{{\rm{e}}}}}}}}}=0.60\,\upmu{{{{{\rm{s}}}}}^{{-1}}}$ and ${{{\Gamma }}}_{\varphi,0}^{{{{{{{{\rm{e}}}}}}}}}=0.09\,\upmu{{{{{\rm{s}}}}}^{{-1}}}$, respectively.

To study the mechanisms determining the energy relaxation time *T*_1_ of the unimon, we measure *T*_1_ as a function of the qubit frequency as shown in Fig. 3b (see also Figs. 7 and 8). At the Φ_diff_ = Φ_0_/2 sweet spot, we find *T*_1_ ≈ 8.6 μs, whereas *T*_1_ ≈ 4.6 μs at Φ_diff_ = 0. Between these flux sweet spots, the relaxation time attains a minimum in a frequency range close to the frequency of the readout resonator *f*_r_ = 6.198 GHz. This behavior of *T*_1_ can be reasonably explained by dielectric losses with an effective quality factor of *Q*_*C*_ ≈ 3.5 × 10^5^ and Purcell decay through the readout resonator (see “Methods” and Supplementary Methods III). This suggests the qubit energy relaxation to be dominated by dielectric losses at Φ_diff_ = Φ_0_/2. The estimated quality factor of this first unimon qubit is almost an order of magnitude higher than for the geometric-superinductance qubits^54^, but of the same order of magnitude as for fluxonium qubits^39,41^ and an order of magnitude lower than in state-of-the-art transmons^26^. Improvements to design, materials, and fabrication processes are expected to reduce the dielectric losses in future unimon qubits compared with the very first samples presented here.

To characterize the sensitivity of the qubit to flux noise, we measure the Ramsey coherence time $${T}_{2}^{*}$$ and the echo coherence time $${T}_{2}^{{{{{{{{\rm{e}}}}}}}}}$$ with a single echo *π*-pulse (see Fig. 8) as a function of the flux bias Φ_diff_. Figure 3c shows that $${T}_{2}^{*}$$ and $${T}_{2}^{{{{{{{{\rm{e}}}}}}}}}$$ are both maximized at Φ_diff_ = Φ_0_/2, reaching 3.1 and 9.2 μs, respectively. Away from the sweet spot, the Ramsey coherence time $${T}_{2}^{*}$$ degrades quickly, but the echo coherence time $${T}_{2}^{{{{{{{{\rm{e}}}}}}}}}$$ stays above 1 μs even if the qubit frequency is tuned from the sweet spot by over 30 MHz. Assuming that the flux noise is described by a 1/*f* noise model $${S}_{{{{\Phi }}}_{{{{{{{{\rm{diff}}}}}}}}}}(\omega )=2\pi {A}_{{{{\Phi }}}_{{{{{{{{\rm{diff}}}}}}}}}}^{2}/\omega$$, we estimate a flux noise density of $${A}_{{{{\Phi }}}_{{{{{{{{\rm{diff}}}}}}}}}}/\sqrt{{{{{{{{\rm{Hz}}}}}}}}}=15.0$$$$\,\mu {{{\Phi }}}_{0}/\sqrt{{{{{{{{\rm{Hz}}}}}}}}}$$ based on the flux dependence of $${T}_{2}^{{{{{{{{\rm{e}}}}}}}}}$$ (“Methods”). The estimated flux noise density is an order of magnitude greater than in state-of-the-art SQUIDS^55^, but an order of magnitude lower than reported for all previous geometric-superinductance qubits^44^.

At Φ_diff_ = 0 in contrast, we measure a Ramsey coherence time of ${T}_{2}^{*}=6.8\,\upmu{{{{{\rm{s}}}}}}$ and a *T*_1_-limited echo coherence time of ${T}_{2}^{{{{{{{{\rm{e}}}}}}}}}=9.9\,\upmu{{{{{\rm{s}}}}}}$. The dephasing rate is lower here than at Φ_diff_ = −Φ_0_/2 since the qubit frequency is less sensitive to the external flux difference due to the lower second-order contribution ∣∂^2^*ω*_01_/∂^2^Φ_diff_∣. Note that the anharmonicity of the qubit at Φ_diff_ = 0 is only *α*/(2*π*) = −58 MHz, and hence this operation point is not of great interest for implementations of high-fidelity quantum logic.

Next, we demonstrate that the high anharmonicity of the unimon and its protection against charge and flux noise enable us to implement fast high-fidelity single-qubit gates. To this end, we calibrate single-qubit gates of duration *t*_g_ ∈ [13.3, 46.6] ns using microwave pulses parametrized according to the derivative removal by adiabatic gate (DRAG) framework^56,57^. To characterize the average fidelity of gates in the set {*I*, *X*(*π*/2), *Y*(*π*/2)}, we utilize interleaved randomized benchmarking^58^ (“Methods”). Figure 4a shows that we reach a practically coherence-limited fidelity of 99.9% for *I*, *X*(*π*/2), and *Y*(*π*/2) gates at 13.3 ns duration. Our electronics limit the shortest gate pulses to 13.3 ns although the anharmonicity should allow for high-fidelity gates down to 5 ns duration corresponding to a gate fidelity of 99.97% with the reported coherence properties.

To study the long-term stability of the gate fidelity, we first calibrate 20 ns single-qubit gates and then conduct repetitive measurements of the average gate fidelity using standard randomized benchmarking^59,60^ without any recalibration between repetitions. Figure 4b indicates that the measured gate fidelity is stable over the full period of eight hours with an average fidelity of 99.88 ± 0.02%, practically coinciding with the coherence limit of 99.89%. This stability can be attributed to the relaxation time *T*_1_ and the coherence times ${T}_{2}^{*}$ and ${T}_{2}^{{{{{{{{\rm{e}}}}}}}}}$ staying practically constant in time as illustrated in Fig. 4c.

## Discussion

In conclusion, we introduced and demonstrated the unimon qubit that has a relatively high anharmonicity while requiring only a single Josephson junction without any superinductors, and bearing protection against both low-frequency charge noise and flux noise. The geometric inductance of the unimon has the potential for higher predictability and reproducibility than the junction-array-based superinductors in conventional fluxoniums or in quartons. Thus, the unimon constitutes a promising candidate for achieving single-qubit gate fidelities beyond 99.99% in superconducting qubits with the help of the following future improvements: (i) redesign of the geometry to minimize dielectric losses^61^ currently dominating the energy relaxation, (ii) use of recently found low-loss materials^26^, and (iii) reduction of the gate duration to values well below 10 ns allowed even by the anharmonicities achieved here. Future unimon research is also needed to study and minimize the various on-chip cross talks, implement two-qubit gates, and to scale up to many-qubit processors. To further reduce the sensitivity of the unimon to flux noise and to scale up the qubit count, it is likely beneficial to reduce the footprint of a single unimon qubit using, e.g., a superconductor with a high kinetic inductance in the coplanar-waveguide resonator. The anharmonicity of the unimon at flux bias Φ_diff_ = Φ_0_/2 has an opposite sign to that of the transmon, which may be helpful to suppress the unwanted residual *ZZ* interaction with two-qubit-gate schemes that utilize qubits with opposite-sign anharmoncities^62,63^. In analogy to the quarton, the dominance of the quartic term in the potential energy of the unimon may enable extremely fast two-qubit gates and qubit readout in schemes utilizing the unimon as a coupler for transmon qubits^64^. The distributed-element nature of the unimon provides further opportunities for implementing a high connectivity and distant couplings in multi-qubit processors. The parameter values we have demonstrated in this work for the qubit–resonator coupling capacitance and for the corresponding coupling strength are sufficient for implementing high-fidelity two-qubit gates employing the typical coupling schemes for transmons^65,66^, as we numerically simulate in Supplementary Note I in Supplementary Materials for the cross-resonance gate. In the future, we also aim to study the utilization of other modes of the unimon circuit, for example, for additional qubits and qubit readout.

## Methods

### Hamiltonian based on a model of coupled normal modes (model 1)

Here, we provide a brief summary of the theoretical model 1 that is used to derive a Hamiltonian for the unimon qubit, starting from the normal modes of the distributed-element circuit illustrated in Fig. 1c. A complete derivation is provided in Supplementary Methods I. In this theoretical model, we extend the approach of ref. 67 to model phase-biased Josephson junctions in distributed-element resonators in the presence of an external magnetic flux.

In the discretized circuit of Fig. 1c, the Josephson junction is located at *x*_J_ and we model the CPW resonator of length 2*l* using *N* inductances *L*_*l*_Δ*x* and *N* capacitances *C*_*l*_Δ*x* with Δ*x* = 2*l*/*N*. Based on this circuit model, we construct the classical Lagrangian of the system using the node fluxes ${{{\Psi }}}_{i}=\int\nolimits_{-\infty }^{t}{V}_{i}(t^{\prime} )\,{{{{{{{\rm{d}}}}}}}}t^{\prime}$ as the coordinates with *V*_*i*_ denoting the voltage across the *i*:th capacitor^68^. From the Lagrangian, we derive the classical equations of motion for the node fluxes and take the continuum limit resulting in a continuous node-flux function $\psi (x)=\int\nolimits_{-\infty }^{t}V(x,\, t^{\prime} )\,{{{{{{{\rm{d}}}}}}}}t^{\prime}$. Under the assumption of a sufficiently homogeneous magnetic field, the flux at the center conductor *ψ*(*x*) is described with the wave equation $\ddot{\psi }={v}_{{{{{{{{\rm{p}}}}}}}}}^{2}{\partial }_{xx}\psi$, where the phase velocity is given by ${v}_{{{{{{{{\rm{p}}}}}}}}}=1/\sqrt{{L}_{l}{C}_{l}}$, where *L*_*l*_ and *C*_*l*_ denote the inductance and capacitance per unit length. Furthermore, we obtain a set of boundary conditions corresponding to the grounding of the CPW at its end points and the current continuity across the junction.

In the regime of small oscillations about a minimum of the potential energy, the classical flux *ψ*(*x*) can be decomposed into a sum of a dc component and oscillating normal modes. Using this decomposition and linearizing the junction in the vicinity of the dc operation point, we derive the classical normal-mode frequencies ${\{{\omega }_{m}/(2\pi )\}}_{m=0}^{\infty }$ and dimensionless flux-envelope functions ${\{{u}_{m}(x)\}}_{m=0}^{\infty }$. Subsequently, we invoke a single-mode approximation, in which the flux *ψ*(*x*) is expressed as *ψ*(*x*, *t*) = Φ_0_*φ*_0_*u*_0_(*x*)/(2*π*) + *ψ*_*m*_(*t*)*u*_*m*_(*x*), where *m* is the mode index corresponding to the qubit, *φ*_0_ is the dc Josephson phase, and *ψ*_*m*_(*t*) describes the temporal evolution of the flux for the qubit mode *m*. The dc phase *φ*_0_ is controlled by the flux bias Φ_diff_ as ${\varphi }_{0}+2l{L}_{l}\sin ({\varphi }_{0})/{L}_{{{{{{{{\rm{J}}}}}}}}}=2\pi {{{\Phi }}}_{{{{{{{{\rm{diff}}}}}}}}}/{{{\Phi }}}_{0}$, where ${L}_{{{{{{{{\rm{J}}}}}}}}}={{{\Phi }}}_{0}^{2}/{(2\pi )}^{2}/{E}_{{{{{{{{\rm{J}}}}}}}}}$ is the effective Josephson inductance.

Finally, we quantize the classical Hamiltonian under the single-mode approximation and obtain

4 $${\hat{H}}_{m}=4{E}_{C,m}({\varphi }_{0}){\hat{n}}_{m}^{2}+\frac{1}{2}{E}_{L,m}({\varphi }_{0}){\hat{\varphi }}_{m}^{2}+{E}_{L}{\hat{\varphi }}_{m}\left(\frac{2\pi {{{\Phi }}}_{{{{{{{{\rm{diff}}}}}}}}}}{{{{\Phi }}}_{0}}-{\varphi }_{0}\right)-{E}_{{{{{{{{\rm{J}}}}}}}}}\cos ({\hat{\varphi }}_{m}-{\varphi }_{0}),$$

where ${\hat{n}}_{m}$ and ${\hat{\varphi }}_{m}$ are the charge and phase operators corresponding to the qubit mode and satisfying $[{\hat{\varphi }}_{m},\,{\hat{n}}_{m}]={{{{{{{\rm{i}}}}}}}}$, ${E}_{L}={{{\Phi }}}_{0}^{2}/{(2\pi )}^{2}/(2l{L}_{l})$ is the inductive energy of the dc component, and the capacitive energy *E*_*C*,*m*_(*φ*_0_) and the inductive energy *E*_*L*,*m*_(*φ*_0_) of the qubit mode *m* are functions of Φ_diff_ and circuit parameters according to Supplementary Eqs. (27), (30–34), (37–38), and (40) in Supplementary Methods I.

The phase-basis wave functions and the potential energy based on the Hamiltonian in Eq. (4) are illustrated in Fig. 5a, b for the parameter values of the qubit B. In Fig. 5c–e, we further show the characteristic energy scales of the unimon (*E*_*C*,*m*_, *E*_*L*,*m*_, *E*_*L*_), the charge matrix elements $\left\langle i\right|{\hat{n}}_{m}\left|\, j\right\rangle$ and the phase matrix elements $\left\langle i\right|{\hat{\varphi }}_{m}\left|j\right\rangle$ as functions of Φ_diff_, where we denote the *k*-photon state of mode *m* by $\left|k\right\rangle$.

In our qubit samples, each unimon is coupled to a readout resonator via a capacitance *C*_g_ at a location *x*_g_ to allow measurements of the qubit state. As derived in Supplementary Methods II, the Hamiltonian of the coupled resonator-unimon system is given by

5 $$\hat{H}=\hslash {\omega }_{{{{{{{{\rm{r}}}}}}}}}{\hat{a}}_{{{{{{{{\rm{r}}}}}}}}}^{{{{\dagger}}} }{\hat{a}}_{{{{{{{{\rm{r}}}}}}}}}+\mathop{\sum}\limits_{j}\hslash {\omega }_{j}|{\,}j\rangle\, \langle \, j |+\hslash \mathop{\sum}\limits_{i,j}\left({g}_{ij}|i\rangle\, \langle \, j|{\hat{a}}_{{{{{{{{\rm{r}}}}}}}}}^{{{{\dagger}}} }+{g}_{ij}^{*}|{\,}j\rangle\, \langle i|{\hat{a}}_{{{{{{{{\rm{r}}}}}}}}}\right),$$

where *f*_r_ = *ω*_r_/(2*π*) is the resonator frequency, ${\hat{a}}_{{{{{{{{\rm{r}}}}}}}}}$ is the annihilation operator of the readout resonator, {*ℏω*_*j*_} and $\{\left|j\right\rangle \}$ are the eigenenergies and eigenstates of the bare unimon qubit, and the coupling strengths *g*_*ij*_ are given by

6 $${g}_{ij}\,\approx\, 4{\omega }_{{{{{{{{\rm{r}}}}}}}}}\frac{{C}_{{{{{{{{\rm{g}}}}}}}}}{u}_{m}({x}_{{{{{{{{\rm{g}}}}}}}}}){{\Delta }}{u}_{m}}{2{C}_{l}l+{C}_{{{{{{{{\rm{J}}}}}}}}}+{C}_{{{{{{{{\rm{g}}}}}}}}}{u}_{m}{({x}_{{{{{{{{\rm{g}}}}}}}}})}^{2}}\sqrt{\frac{{Z}_{{{{{{{{\rm{tr}}}}}}}}}}{{R}_{K}}}\left\langle i\right|{{{{{{{\rm{i}}}}}}}}{\hat{n}}_{m}\left|j\right\rangle,$$

where ${{\Delta }}{u}_{m}={u}_{m}({x}_{{{{{{{{\rm{J}}}}}}}}}^{+})-{u}_{m}({x}_{{{{{{{{\rm{J}}}}}}}}}^{-})$, *C*_J_ is the junction capacitance, ${Z}_{{{{{{{{\rm{tr}}}}}}}}}$ is the characteristic impedance of the resonator, and *R*_*K*_ = *h*/*e*^2^ is the von Klitzing constant. Assuming that ∣*ω*_1_ − *ω*_0_ − *ω*_*r*_∣ ≫ ∣*g*_01_∣, we invoke the dispersive approximation allowing us to simplify Eq. (5) as (see Supplementary Methods II)

7 $${\hat{H}}_{{{{{{{{\rm{disp}}}}}}}}}\,\approx\, \hslash \omega_{{{\rm{r}}}}^{\prime} {\hat{a}}_{{{{{{{{\rm{r}}}}}}}}}^{{{{\dagger}}} }{\hat{a}}_{{{{{{{{\rm{r}}}}}}}}}-\frac{\hslash \omega_{01}^{\prime} }{2}{\hat{\sigma }}_{z}-\hslash \chi {\hat{a}}_{{{{{{{{\rm{r}}}}}}}}}^{{{{\dagger}}} }{\hat{a}}_{{{{{{{{\rm{r}}}}}}}}}{\hat{\sigma }}_{z},$$

where $\omega_{{{\rm{r}}}}^{\prime}$ and $\omega_{01}^{\prime}$ are the renormalized resonator and qubit frequencies, ${\hat{\sigma }}_{z}=\left|0\right\rangle \left\langle 0\right|-\left|1\right\rangle \left\langle 1\right|$, and the dispersive shift *χ* is approximately given by

8 $$\chi=\frac{|{g}_{01}{|}^{2}}{{\omega }_{1}-{\omega }_{0}-{\omega }_{{{{{{{{\rm{r}}}}}}}}}}-\frac{1}{2}\frac{|{g}_{12}{|}^{2}}{{\omega }_{2}-{\omega }_{1}-{\omega }_{{{{{{{{\rm{r}}}}}}}}}}.$$

Although the dispersive approximation involves a minor transformation of the qubit and resonator operators, we have for simplicity used identical symbols for the transformed and original operators.

### Hamiltonian based on a path integral approach (model 2)

Here, we summarize our alternative theoretical approach for evaluation of the unimon spectrum. The unimon consists of a non-linear element (the Josephson junction) embedded into a linear non-dissipative environment (the *λ*/2 resonator) as shown in Fig. 1. This environment can be integrated out by the means of a path-integral formalism resulting in an effective action for a single variable, the flux difference *ψ*_−_ across the junction. This action appears to be both non-Gaussian and non-local in imaginary time, and hence extremely challenging to integrate it analytically. In order to obtain the low-frequency spectrum of the unimon, we approximate the non-local part of the action by coupling the *ψ*_−_ degree of freedom to *M* auxiliary linear modes, each described by a flux coordinate *χ*_*k*_, *k* = 1, …, *M*. As described in detail in the Supplementary Methods I, the effective Hamiltonian of the unimon in this model reads as

9 $${\hat{H}}_{M}=\frac{\hat{{Q}_{-}^{2}}}{2C}+\frac{{\hat{\psi }}_{-}^{2}}{2{L}_{\psi }}-{E}_{{{{{{{{\rm{J}}}}}}}}}\cos \left[\frac{2\pi }{{{{\Phi }}}_{0}}\left({\hat{\psi }}_{-}+{{{\Phi }}}_{{{{{{{{\rm{diff}}}}}}}}}\right)\right]+\mathop{\sum }\limits_{k=1}^{M}\left[\frac{{\hat{q}}_{k}^{2}}{2C}+\frac{C{\hat{\chi }}_{k}^{2}}{2}{\left(\frac{\pi k{v}_{{{{{{{{\rm{p}}}}}}}}}}{2l}\right)}^{2}+{\alpha }_{k}{\hat{\chi }}_{k}{\hat{\psi }}_{-}\right],$$

where $[{\hat{\chi }}_{k},{\hat{q}}_{m}]={{{{{{{\rm{i}}}}}}}}\hslash {\delta }_{km}$, $[{\hat{\psi }}_{-},{\hat{Q}}_{-}]={{{{{{{\rm{i}}}}}}}}\hslash$, and all other single-operator commutators are zero, and the parameters *C*, *L*_*ψ*_, and *α*_*k*_ are determined by Supplementary Eqs. (76)–(78). In the limit *M* → *∞*, this approximation becomes exact. We restrict our analysis to the lowest auxiliary mode which gives a non-vanishing contribution to the unimon spectrum. Note that if the unimon is symmetric (*x*_J_ = 0), the coupling of the Josephson junction to the first mode of the resonator vanishes, i.e., *α*_1_ = 0, and hence we need to consider the case *M* = 2. This approximation defines our model 2 which appears accurate enough for the quantitative analysis of the experimental data.

In addition to the technicalities related to the derivation of the models, the main difference between models 1 and 2 lies within the different employed approximations. In model 1, we take the linear part of the unimon into account exactly after linearizing the circuit at the minimum of the potential given by the dc phase, but we apply the single-mode approximation. Model 2 does not require us to solve the dc phase, and consequently we can conveniently work also in the regime *E*_J_ > *E*_*L*_ which is problematic for model 1 owing to multiple solutions for the dc phase. The price we pay for this advantage is that we consider the linear part of the problem to some extent approximately and that we need to solve a multidimensional Schrödinger equation.

### Design of the qubit samples

The samples are designed using KQCircuits^69^ software which is built to work with the open-source computer-automated-design program KLayout^70^. The designs are code-generated and parametrized for convenient adjustments during the design process. As illustrated in Fig. 1e, each of the qubit chips comprise three unimon qubits which are capacitively coupled to individual readout resonators via U-shaped capacitors. All readout resonators are coupled with finger capacitors to the probe line using a single waveguide splitter. For multiplexed readout, the frequencies of the readout resonators are designed to be separated by 300 MHz. All of the unimons have the Josephson junction at the mid-point of the waveguide and are capacitively coupled to individual drive lines.

We present the design values of the main characteristic properties for all of the measured five qubits in Supplementary Table 2 in Supplementary Methods V. To obtain the geometries of the qubit circuits that yield the desired physical properties, first, the dimensions of the center conductor of the qubit are chosen in an effort to obtain the characteristic impedance of $Z=\sqrt{{L}_{l}/{C}_{l}}=100$ Ω. Here, the capacitance per unit length is *C*_*l*_ = 2*ϵ*_0_(*ϵ*_*r*_ − 1)*r*_1_ + *C*_air_ and the inductance per unit length is *L*_*l*_ = 1/(*C*_air_*c*^2^), where *ϵ*_0_ is the vacuum electric permittivity, *ϵ*_*r*_ = 11.45 is the relative dielectric constant of the substrate, ${r}_{1}=K({r}_{2}^{2})/K(1-{r}_{2}^{2})$, where *K* denotes the complete elliptic integral of the first kind, ${r}_{2}=\tanh [\pi a/(4\eta )]/\tanh [\pi b/(4\eta )]$, *a* is the width of the center conductor of the qubit, *η* is the thickness of the substrate, *b* is the total width of the qubit waveguide, *C*_air_ = 2*ϵ*_0_(*r*_1_ + *r*_3_), where ${r}_{3}=K({r}_{4}^{2})/K(1-{r}_{4}^{2})$, *r*_4_ = *a*/*b*, and *c* is the speed of light^71^. Second, a series of finite-element simulations is executed on Ansys Q3D Extractor software to obtain the dimensions of the U-shaped capacitors with the target values of approximately 10 fF for the coupling capacitances *C*_g_ between the readout resonators and the qubits. Third, the dispersive shift of the qubit is approximated based on Eq. (8) as *χ* = *α*∣*g*_01_∣^2^/[Δ(Δ + *α*)], where *α*/(2*π*) = 500 MHz is a rough estimate for the anharmonicity of the unimon, ∣*g*_01_∣/(2*π*) is the targeted coupling strength between the qubit and its readout resonator, and Δ = 2*π*(*f*_01_ − *f*_r_). Finally, we adjust the length of the readout resonator and the capacitance *C*_*κ*_ between the resonator and the probe line in order to obtain a resonator linewidth of *κ* ≈ *χ* and a resonator frequency of *f*_r_. To this end, we carry out the microwave modeling of the device netlist, from which we obtain estimates for the resonant modes and their respective linewidths.

### Sample fabrication

The qubit devices were fabricated at the facilities of OtaNano Micronova cleanroom. First, we sputter a 200 nm-thick layer of highly pure Nb on a high-resistivity (*ρ* > 10 kΩcm) non-oxidized undoped *n*-type (100) 6-inch silicon wafer. Then, the coplanar waveguide is defined in a mask aligner using photo resist. After development, the Nb film is etched with a reactive ion etching (RIE) system. After etching, the resist residuals are cleaned in ultrasonic bath with acetone and isopropyl alcohol (IPA), and dried with a nitrogen gun. Subsequently, the 6-inch wafer is cleaved into 3 × 3 cm^2^ dices by Disco DAD3220, including nine chips in total. Each chip is 1 × 1 cm^2^.

The tunnel junctions are patterned by a 100 keV EPBG5000pES electron beam lithography (EBL) system with a bilayer of methyl methacrylate/poly methyl methacrylate (MMA/PMMA) resist on a single chip. This is followed by a development in a solution of Methyl isobutyl ketone (MIBK) and IPA (1:3) for 20 s, Methyl Glycol for 20 s, and IPA for 20 s. The resist residues are cleaned with oxygen descum for 15 s. The two-angle shadow evaporation technique is applied to form the SIS junctions in an electron beam evaporator. Before evaporation, the native oxides are removed by Ar ion milling. Aluminum is deposited at a rate of 5 Å/s. After lift off in acetone, each chip is cleaved by Disco DAD3220, then packaged and bonded with Al wires.

### Measurement setup

For the experimental characterization, the packaged qubit devices are cooled down to a temperature of 10 mK using a commercial dilution refrigerator. The packaged samples are shielded by nested mu-metal and Aluminum shields. The ports of the sample holder are connected to room temperature electronics according to the more detailed schematic diagram that can be found in Supplementary Methods IV.

To implement the microwave signals for driving the qubits, we up-convert in-phase (*I*) and quadrature-phase (*Q*) waveforms generated by an arbitrary waveform generator with the help of an *IQ* mixer and a local oscillator signal. The generated microwave signal is passed through a room temperature dc block and 60 dB of attenuation within the cryostat before reaching the sample.

For the qubit-state readout, we use an ultrahigh-frequency quantum analyzer (UHFQA) by Zurich Instruments. Using the UHFQA, we create an intermediate-frequency voltage signal that is up-converted to the frequency of the readout resonator with an *IQ* mixer and a local oscillator. The obtained microwave signal is passed through 60 dB of attenuation within the cryostat before entering the probe line. The output readout signal passes through two microwave isolators and a cryogenic high-electron-mobility transistor (HEMT) for amplification. At room temperature, the output signal is further amplified using a series of amplifiers and down-converted back to an intermediate frequency. In the UHFQA, the down-converted voltage signal is digitized and numerically converted to the base band. Due to the qubit-state-dependent dispersive shift of the readout resonator [see Eq. (7)], the measured output voltage is also dependent on the qubit state. To enable convenient calibration of the *IQ* mixer used for the qubit drive, the setup also includes a room temperature switch enabling us to alternatively down-convert and measure the up-converted drive signal.

To control the external flux difference, we use an external coil connected to a dc voltage source via two 50 kΩ resistors and a series of low-pass filters at room temperature and at the 100 mK stage of the cryostat. The coil is not specifically designed to yield a magnetic-field gradient required to bias the unimon, but such a gradient forms naturally owing to the simple circular shape of the coil and to the field-screening superconducting regions in the vicinity of the qubit. Note that the field does not need to be constant along the CPW structure although we have, for simplicity, invoked such an assumption in the derivation of model 1 in Supplementary Methods I.

### Measurement and analysis of qubit frequency and anharmonicity

To measure the frequencies of the one-photon $\left|0\right\rangle \leftrightarrow \left|1\right\rangle$ transition and the two-photon $\left|0\right\rangle \leftrightarrow \left|2\right\rangle$ transition, we use a standard two-tone qubit spectroscopy experiment illustrated in Fig. 2c. In the experiment, we apply a continuous microwave signal to the drive line of the qubit while applying a readout signal through the probe line of the sample. At the sweet spot Φ_diff_ = Φ_0_/2, we further measure the $\left|1\right\rangle \leftrightarrow \left|2\right\rangle$ transition frequency with an ef-Rabi experiment (see Fig. 6) in order to verify the anharmonicities shown in Fig. 3a and summarized in Table 1. In the ef-Rabi experiment, the qubit is first prepared to the excited state with a *π*-pulse followed by another pulse with a varying amplitude and a varying frequency around the estimated $\left|1\right\rangle \leftrightarrow \left|2\right\rangle$ transition. After the drive pulses, a readout pulse is applied and an oscillating output voltage is observed as a result of Rabi oscillations between the states $\left|1\right\rangle$ and $\left|2\right\rangle$.

To estimate the circuit parameters presented in Table 1, we use the following approach. First, we fit the theoretical Hamiltonian in Eq. (4) to the experimental transition frequencies of qubit B in order to estimate *L*_*l*_, *C*_*l*_, and *E*_J_. Subsequently, the coupling capacitance *C*_g_ of qubit B is estimated by fitting Eq. (5) to the data of the avoided crossing in Fig. 2b. For the other qubits, it is assumed that *L*_*l*_ and *C*_*l*_ are equal to those of qubit B due to an identical geometry of the CPW. For these qubits, the Josephson energy *E*_J_ is first approximately fitted based on the measured $\left|0\right\rangle \leftrightarrow \left|1\right\rangle$ transition followed by an estimation of *C*_g_ using data of an avoided unimon–resonator crossing.

### Characterization for readout

To characterize the device for qubit readout, we measure the dispersive shift *χ*/(2*π*) for all of the qubits. This is achieved using an experiment, in which the output readout signal is measured as a function of the signal frequency after preparing the qubit either to its ground or first excited state. In Fig. 7a, the measured dispersive shifts are compared against theoretical predictions computed with Eq. (8) based on the fitted circuit parameters, and the measured qubit frequency *ω*_01_/(2*π*) and anharmonicity *α*/(2*π*). The good agreement between the experiment and the theory validates the dispersive approximation in Eq. (7).

We further measure the single-shot readout fidelity for qubit E with *χ*/(2*π*) = 4.1 MHz. This is achieved by alternately preparing the qubit to the ground state and to the first excited state followed by a state measurement with a 1.6 μs-long readout pulse. The output readout voltage is obtained as an unweighted average of the voltage during a 1.6 μs-long integration window. This experiment is repeated 2000 times. Using an optimized threshold voltage, we extract a readout fidelity $[P(\left|0\right\rangle|\left|0\right\rangle )+P(\left|1\right\rangle|\left|1\right\rangle )]/2$ of 89.0% as shown in Fig. 7b, c. The readout error is dominated by qubit relaxation during the readout pulse. Note that the measured fidelity is reached without a quantum-limited amplifier suggesting that high-fidelity single-shot readout is possible with the unimon. Similarly, the relatively long readout time used in this work can be greatly shortened after the introduction of a quantum-limited amplifier.

### Measurement and analysis of energy relaxation time

To measure the energy relaxation time *T*_1_, an initial *π*-pulse is applied to the ground-state-initialized qubit followed by a varying delay and a subsequent measurement of the qubit population. We use a single exponential function for fitting the qubit population, which is supported by the experimental data of qubit B shown in Fig. 8a. Thus, there is no evidence of quasiparticle-induced losses that result in a double-exponential decay.

For qubit B, the relaxation time is characterized across Φ_diff_/Φ_0_ ∈ [0.0, 0.5] in order to determine the mechanisms limiting *T*_1_. As detailed in Supplementary Methods III, we model the relaxation rate Γ_1_ = 1/*T*_1_ due to a noise source *λ* as^72^

10 $${{{\Gamma }}}_{1}=\frac{|\left\langle 0\right|\partial {\hat{H}}_{m}/\partial \lambda \left|1\right\rangle {|}^{2}}{{\hslash }^{2}}{S}_{\lambda }({\omega }_{01}),$$

where *S*_*λ*_(*ω*_01_) is the symmetrized noise spectral density of the variable *λ* at the qubit angular frequency *ω*_01_. In Fig. 8b, we compare the frequency dependence of the measured relaxation rate to the theoretical models based on Ohmic flux noise, 1/*f* flux noise, dielectric losses, inductive losses, radiative losses, and Purcell decay through the resonator by scaling the theoretical predictions to coincide with the experimental data at Φ_diff_ = Φ_0_/2. As illustrated in Fig. 3b, the experimental data is most accurately explained by a model including Purcell decay and dielectric losses with an effective dielectric quality factor of *Q*_*C*_ = 3.5 × 10^5^.

### Measurement and analysis of coherence time

The coherence time of the qubits is characterized using standard Ramsey and Hahn echo measurements^73^. At the sweet spots, we estimate the Ramsey coherence time ${T}_{2}^{*}$ by fitting an exponentially decaying sinusoidal function to the measured qubit population, whereas we obtain the echo coherence time ${T}_{2}^{{{{{{{{\rm{e}}}}}}}}}$ using an exponential fit. As illustrated in Fig. 8c–e, these models agree well with the experimental data of qubit B at the flux-insensitive sweet spots yielding ${T}_{2}^{*}=3.1\,\upmu{{{{{\rm{s}}}}}}$ and ${T}_{2}^{{{{{{{{\rm{e}}}}}}}}}=8.9\,\upmu{{{{{\rm{s}}}}}}$ for Φ_diff_ = Φ_0_/2, and ${T}_{2}^{*}=6.4\,\upmu{{{{{\rm{s}}}}}}$ and ${T}_{2}^{{{{{{{{\rm{e}}}}}}}}}=9.5\,\upmu{{{{{\rm{s}}}}}}$ for Φ_diff_ = 0.

To study the sensitivity of the qubits to flux noise, we conduct Ramsey and Hahn echo measurements as a function of the external flux bias in the vicinity of Φ_diff_ = Φ_0_/2 (see Fig. 3c). In superconducting qubits, flux noise is often accurately described by 1/*f* noise^74,75^

11 $${S}_{{{{\Phi }}}_{{{{{{{{\rm{diff}}}}}}}}}}(\omega )=\int\nolimits_{-\infty }^{\infty }{{{{{{{\rm{d}}}}}}}}t\exp (-{{{{{{{\rm{i}}}}}}}}\omega t)\langle {{{\Phi }}}_{{{{{{{{\rm{diff}}}}}}}}}(0){{{\Phi }}}_{{{{{{{{\rm{diff}}}}}}}}}(t)\rangle=2\pi \frac{{A}_{{{{\Phi }}}_{{{{{{{{\rm{diff}}}}}}}}}}^{2}}{\omega },$$

where ${A}_{{{{\Phi }}}_{{{{{{{{\rm{diff}}}}}}}}}}/\sqrt{{{{{{{{\rm{Hz}}}}}}}}}$ is the flux noise density at 1 Hz. The 1/*f*-noise gives rise to a Gaussian decay in the echo experiment^55,76^, due to which we model the Hahn echo decay with a product of Gaussian and exponential functions, $\propto \exp (-{{{\Gamma }}}_{\varphi,{{\Phi }}}^{{{{{{{{\rm{e}}}}}}}}}{t}^{2}-{{{\Gamma }}}_{\varphi,0}^{{{{{{{{\rm{e}}}}}}}}}t)$, as illustrated in Fig. 8f. The corresponding ${T}_{2}^{{{{{{{{\rm{e}}}}}}}}}$ is evaluated as the 1/e decay time given by^39^

12 $${T}_{2}^{{{{{{{{\rm{e}}}}}}}}}=\frac{\sqrt{4{({{{\Gamma }}}_{\varphi,{{\Phi }}}^{{{{{{{{\rm{e}}}}}}}}})}^{2}+{({{{\Gamma }}}_{\varphi,0}^{{{{{{{{\rm{e}}}}}}}}})}^{2}}-{{{\Gamma }}}_{\varphi,0}^{{{{{{{{\rm{e}}}}}}}}}}{2{({{{\Gamma }}}_{\varphi,{{\Phi }}}^{{{{{{{{\rm{e}}}}}}}}})}^{2}}.$$

Under the assumption of 1/*f*-noise, the Gaussian dephasing rate ${{{\Gamma }}}_{\varphi,{{\Phi }}}^{{{{{{{{\rm{e}}}}}}}}}$ obtained from an echo measurement is related to the flux noise density as^55,76^

13 $${{{\Gamma }}}_{\varphi,{{\Phi }}}^{{{{{{{{\rm{e}}}}}}}}}=\sqrt{\ln 2}{A}_{{{{\Phi }}}_{{{{{{{{\rm{diff}}}}}}}}}}\left|\right.\frac{\partial {\omega }_{01}}{\partial {{{\Phi }}}_{{{{{{{{\rm{diff}}}}}}}}}}\left|\right.+{{{\Gamma }}}_{\varphi,{{{{{{{\rm{x}}}}}}}}}^{{{{{{{{\rm{e}}}}}}}}},$$

where ${{{\Gamma }}}_{\varphi,x}^{{{{{{{{\rm{e}}}}}}}}}$ is a small residual Gaussian decay rate at the sweet spot. For each of the qubits, we estimate the parameter ${A}_{{{{\Phi }}}_{{{{{{{{\rm{diff}}}}}}}}}}$ in Table 2 by a linear least-squares fit to $(|\partial {\omega }_{01}/\partial {{{\Phi }}}_{{{{{{{{\rm{diff}}}}}}}}}|,{{{\Gamma }}}_{\varphi ,{{\Phi }}}^{{{{{{{{\rm{e}}}}}}}}})$ data, where ∂*ω*_01_/∂Φ_diff_ is estimated by fitting a parabola ${\omega }_{01}=\tilde{a}{{{{\Phi }}}_{{{{{{{{\rm{diff}}}}}}}}}}^{2}+\tilde{b}{{{\Phi }}}_{{{{{{{{\rm{diff}}}}}}}}}+\tilde{c}$ to the measured *ω*_01_ near the sweet spot and then evaluating $\partial {\omega }_{01}/\partial {{{\Phi }}}_{{{{{{{{\rm{diff}}}}}}}}}=2\tilde{a}{{{\Phi }}}_{{{{{{{{\rm{diff}}}}}}}}}+\tilde{b}$.

For Ramsey experiments, we use an exponential decay model also away from the sweet spot to constrain the number of fitting parameters. The theoretical fit shown in Fig. 3c is based on a simple model of the form $1/{T}_{2}^{*}=a^{\prime}|\partial {\omega }_{01}/\partial {{{\Phi }}}_{{{{{{{{\rm{diff}}}}}}}}} |+b^{\prime}$.

### Implementation and benchmark of single-qubit gates

To implement fast high-fidelity single-qubit gates, we use the derivative removal by adiabatic gate (DRAG) framework^56^. Thus, we parametrize the microwave pulses implementing the gates ${V}_{{{{{{{{\rm{rf}}}}}}}}}(t)={I}_{{{{{{{{\rm{qb}}}}}}}}}(t)\cos ({\omega }_{{{{{{{{\rm{d}}}}}}}}}t+\theta )+{Q}_{{{{{{{{\rm{qb}}}}}}}}}(t)\sin ({\omega }_{{{{{{{{\rm{d}}}}}}}}}t+\theta )$ as

14 $${I}_{{{{{{{{\rm{qb}}}}}}}}}(t)=A\exp \left[\frac{-{(t-{t}_{{{{{{{{\rm{g}}}}}}}}}/2)}^{2}}{2{\sigma }^{2}}\right],\,t\in [0,\, {t}_{{{{{{{{\rm{g}}}}}}}}}],$$

15 $${Q}_{{{{{{{{\rm{qb}}}}}}}}}(t)=\beta I_{{{\rm{qb}}}}^{\prime} (t),\,t\in [0,\, {t}_{{{{{{{{\rm{g}}}}}}}}}],$$

where *ω*_d_/(2*π*) is the drive frequency, *θ* determines the rotation axis of the gate, *A* and *β* are amplitudes of *I*_qb_ and *Q*_qb_, respectively, *t*_g_ is the gate duration, and *σ* = *t*_g_/4 is the standard deviation of the Gaussian. The drive frequency *ω*_d_/(2*π*) is set to the qubit frequency *ω*_01_/(2*π*) measured in a Ramsey experiment. The amplitude *A* of the Gaussian pulse is determined using error amplification by applying repeated *π* pulses with varying amplitudes *A* after an initial *π*/2 pulse. The amplitude *β* of the derivative component is chosen to minimize the difference of qubit populations measured after gate sequences (*X*(*π*), *Y*(*π*/2)) and (*Y*(*π*), *X*(*π*/2))^77^.

To characterize the accuracy of the calibrated single-qubit gates, we use the definition of average gate fidelity^78^. To measure the average gate fidelity, we use standard and interleaved randomized benchmarking (RB) protocols^58–60^. In the standard RB protocol, we apply random sequences of Clifford gates appended with a final inverting gate and estimate the average fidelity of gates in the Clifford group *F*_Cl_ based on the decay rate of the ground state probability as a function of the sequence length. We decompose the Clifford gates based on Table 1 in ref. 79 using the native gate set {*I*, *X*(±*π*/2), *Y*(±*π*/2), *X*(*π*), *Y*(*π*)} such that each Clifford gate contains on average 1.875 native gates. The average fidelity per a single native gate is estimated as *F*_g_ = 1 − (1 − *F*_Cl_)/1.875. To estimate the average gate fidelity of individual gates in the set {*I*, *X*(*π*/2), *Y*(*π*/2)}, we utilize the interleaved RB protocol, in which the average gate fidelity is measured by comparing the decay rates for sequences with and without the gate of interest interleaved after each random Clifford gate.

The theoretical coherence limit for the gate fidelity is computed based on the measured *T*_1_ and ${T}_{2}^{{{{{{{{\rm{e}}}}}}}}}$ as ${F}_{{{{{{{{\rm{g,lim}}}}}}}}}=1/6\times \left(3+\exp (-{t}_{{{{{{{{\rm{g}}}}}}}}}/{T}_{1})+2\exp (-{t}_{{{{{{{{\rm{g}}}}}}}}}/{T}_{2}^{{{{{{{{\rm{e}}}}}}}}})\right)$^38^.

## Supplementary information

Supplementary Information

## Data Availability

Data supporting the findings of this article is available at 10.5281/zenodo.7052804.

## Code availability

The authors declare that the theoretical results used in the numerical algorithms for obtaining the findings of this study are available within the paper and its supplementary information.
