# Supplementary material for: Unimon qubit
Source: Nat Commun. 2022 Nov 12;13:6895. doi: 10.1038/s41467-022-34614-w (PMC9653402; doi:10.1038/s41467-022-34614-w)
Supplement: Supplementary file 1 — Supplementary Information [file 41467_2022_34614_MOESM1_ESM.pdf]

Supplementary Information for

Unimon qubit

Eric Hyyppä[1,*], Suman Kundu[2], Chun Fai Chan[1], András Gunyhó[2], Juho Hotari[1], David Janzso[1], Kristinn Juliusson[1], Olavi Kiuru[2], Janne Kotilahti[1], Alessandro Landra[1], Wei Liu[1], Fabian Marxer[1], Akseli Mäkinen[1], Jean-Luc Orgiazzi[1], Mario Palma[1], Mykhailo Savytskyi[1], Francesca Tosto[1], Jani Tuorila[1], Vasilii Vadimov[2], Tianyi Li[1], Caspar Ockeloen-Korppi[1], Johannes Heinsoo[1, †], Kuan Yen Tan[1, †], Juha Hassel[1, †], and Mikko Möttönen[1,2,3,*, †]

[1]IQM, Keilaranta 19, 02150 Espoo, Finland

[2]QCD Labs, QTF Centre of Excellence, Department of Applied Physics, Aalto University, P.O. Box 13500, FIN-00076 Aalto, Finland.

[3]VTT Technical Research Centre of Finland Ltd. & QTF Centre of Excellence, P.O. Box 1000, 02044 VTT, Finland.

[*]Corresponding authors. e-mails: eric@meetiqm.com, mikko.mottonen@aalto.fi

[†]Jointly supervised the work

Supplementary Methods I: Derivation for the Hamiltonian of the unimon qubit using a distributed-element circuit model

In this section, we provide two different derivations for the Hamiltonian of the unimon qubit, both starting from the distributed-element circuit model. These theoretical models correspond to models 1 and 2 in the main text. We begin with model 1 that is based on expressing the Hamiltonian in the basis of interacting classical normal modes that are obtained by linearizing the circuit in the vicinity of its potential energy minimum. Subsequently, we proceed to model 2 which utilizes the path integral formalism to analytically eliminate exactly solvable linear parts of the Lagrangian and to approximate the remaining action with the help of a truncated polynomial. Whereas model 1 does not apply as such in the case of multiple potential minima, no extensions to model 2 are required in such a regime which is, however, not studied experimentally in this work.

Gradiometricity and classical treatment of the dc supercurrent

First, we study the dc response of the unimon circuit in the presence of an external magnetic flux in order to justify why the circuit is gradiometric, and hence it is only sensitive to the external flux difference. To this end, we first consider the lumped-element circuit model for the currents and fluxes shown in Supplementary Fig. **1**. The capacitance can be neglected in the case of dc currents. In our model, we denote the total flux through the left (right) loop of the grounded coplanar waveguide (CPW) structure of the unimon by Φ_1 (Φ_2). The loop flux Φ_1 is given by a sum of the external flux and the flux generated by the induced supercurrents as

$$\Phi_1 = \Phi_{\mathrm{ext},1} + L_{\mathrm{g}} I_1 + L I_{\mathrm{J}}, \tag{1}$$

where $\Phi_{\mathrm{ext},1}$ is the external dc magnetic flux through the left loop, L_{g} is the geometric inductance of the left branch of the ground loop, L is the total inductance of the center conductor, and the currents I_1 and I_{J} are defined as in Supplementary Fig. **1**. Similarly, the total dc flux through the right loop Φ_2 is given by

$$\Phi_2 = \Phi_{\mathrm{ext},2} - L_{\mathrm{g}} I_2 - L I_{\mathrm{J}}, \tag{2}$$

where $\Phi_{\mathrm{ext},2}$ is the external dc magnetic flux through the right loop, and I_2 is the current in the right branch. Note that we assume that the left and right branch of the ground loop are symmetric.

Importantly, we model the outer loop as an ideal superconducting loop with a geometric inductance of L_{g} in both arms of the loop. Due to the phenomenon of flux quantization[1,2], the total dc flux $\Phi_1 + \Phi_2$ must be quantized in units of the flux quantum

$$\Phi_1 + \Phi_2 = \Phi_{\mathrm{ext},1} + \Phi_{\mathrm{ext},2} + L_{\mathrm{g}}(I_1 - I_2) = n\Phi_0 = 0, \tag{3}$$

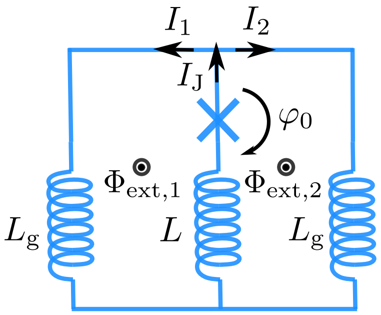

Supplementary Fig. 1: **Direct-current circuit model of the unimon.** Circuit model used for deriving the flux quantization condition of the unimon circuit in the dc regime. Here, L denotes the lumped-element inductance of the center conductor of the CPW resonator, $L_{\rm g}$ denotes the inductance of the right and left arms of the outer superconducting loop, $I_{\rm J}$ denotes the dc current through the Josephson junction embedded in the center conductor of the CPW resonator, and I_1 and I_2 denote the dc current in the left and right branch of the outer superconducting loop, respectively. Furthermore, $\Phi_{\rm ext,1}$ and $\Phi_{\rm ext,2}$ denote the external dc magnetic flux through the left and right superconducting loop, respectively, and φ_0 denotes the dc superconducting phase difference across the Josephson junction due to the external magnetic flux. Note that the used inductor symbols do not show the handedness of the inductors which is defined by equations (1) and (2).

where $\Phi_0 = h/(2e) \approx 2.067 \times 10^{-15}$ Wb is the flux quantum, and we have set $n = 0$ in the last step. Naturally, this implies that the fluxes through the left and right loop must satisfy $\Phi_1 = -\Phi_2$. By considering the flux quantization through the left loop, we obtain an equation that relates the dc phase across the Josephson junction to Φ_1 as

$$\Phi_1 + \frac{\Phi_0}{2\pi}\varphi_0 = 0 \Rightarrow \varphi_0 = -\frac{2\pi}{\Phi_0}\Phi_1, \tag{4}$$

where we have denoted the dc Josephson phase across the Josephson junction with the symbol φ_0. Note that an identical equation is obtained by considering the right superconducting loop.

By combining the information in equations (1)–(4) and using the Kirchhoff current law $I_1 + I_2 = I_{\rm J}$, we find that the dc phase across the Josephson junction reads

$$\varphi_0 = \frac{2\pi}{\Phi_0}[\Phi_{\rm diff} - (L + L_{\rm g}/2)I_{\rm J}], \tag{5}$$

where we have additionally introduced the shorthand notation $\Phi_{\rm diff} = (\Phi_{\rm ext,2} - \Phi_{\rm ext,1})/2$ for the half difference of the external fluxes through the right and the left loop. Importantly, the dc current across the Josephson junction is given by the dc Josephson relation $I_{\rm J} = I_{\rm c}\sin(\varphi_0)$, where $I_{\rm c}$ is

the critical current of the junction. Consequently, the dc phase across the Josephson junction is obtained as a solution of the transcendental equation

$$\varphi_0 + \frac{L_{\mathrm{CPW}}}{L_{\mathrm{J}}}\sin(\varphi_0) = 2\pi\frac{\Phi_{\mathrm{diff}}}{\Phi_0}, \tag{6}$$

where we have used the expression for the Josephson inductance $L_{\mathrm{J}} = \Phi_0/(2\pi I_{\mathrm{c}})$ and defined the shorthand notation $L_{\mathrm{CPW}} = L + L_{\mathrm{g}}/2$. Importantly, the Josephson phase across the junction is controlled by the difference of the fluxes through the two loops since the circuit contains a gradiometric loop. Since the Josephson phase is only affected by the difference of the external fluxes, the circuit and the corresponding qubit are protected against flux noise which is homogeneous over the length scale of the transverse width of the CPW resonator.

Note that the equation has a single-valued solution for all values of the flux bias Φ_{diff} if and only if the inductance of the junction L_{J} dominates over the inductance L_{CPW} of the CPW. This single-valuedness is used below to express the Hamiltonian in terms of the normal modes of the circuit which is linearized around the dc phase. If there are two or more solutions to Eq. (6), the treatment becomes more complex. Thus for model 1, we refrain from going into the multi-valued regime. However, we observe below that for model 2, no special issues arise even in the multivalued regime.

Derivation of the quantum Hamiltonian of coupled normal modes for the unimon qubit (model 1)

To derive the Hamiltonian for the unimon circuit, we use the discretized circuit model visualized in Supplementary Fig. **2**. Our theoretical approach is inspired by Ref.[3], in which the Hamiltonian is derived for a system consisting of a floating CPW resonator with an embedded Josephson junction in its center conductor. However, we extend the theory to model phase-biased Josephson junctions in order to consider effects arising from an external magnetic field. To summarize our derivation of the Hamiltonian, we first derive classical equations of motion for the distributed-element system. Subsequently, we find the classical normal modes of the system by treating the phase-biased Josephson junction as a linear inductor. After finding the classical normal modes, we make a single-mode approximation, in which we neglect all other modes apart from the mode that is used as a qubit. Finally, the classical single-mode Hamiltonian is quantized by enforcing the canonical commutation relation between phase and charge operators, as a result of which we obtain an accurate approximation for the Hamiltonian of the unimon qubit.

In the discretized circuit diagram shown in Supplementary Fig. **2**, we model the CPW of

length $2l$ by N lumped-element inductors and capacitors. The Josephson junction at $x_\mathrm{J} \in (-l, l)$ is taken to be located between the capacitors with indices J and $J+1$. Due to the gradiometricity of the circuit, the total external flux in the circuit model corresponds to the half difference of the external fluxes on the two sides of the center conductor in line with the derivation of the previous section. We use classical circuit theory to derive the equations of motion for the system and to further compute the frequencies and the envelope functions of the normal modes. The equations of motion are obtained from the classical Lagrangian

$$\mathcal{L} = T - U, \tag{7}$$

where T and U are the kinetic and the potential energy, respectively. By choosing the node fluxes Ψ_i as the generalized free coordinates, the total kinetic energy of the circuit can be written as

$$T = \sum_{i=1}^{N} \frac{1}{2} C_l \Delta x \dot{\Psi}_i^2 + \frac{1}{2} C_\mathrm{J} (\dot{\Psi}_{J+1} - \dot{\Psi}_J)^2 \tag{8}$$

$$\xrightarrow[N\to\infty]{} \int_{-l}^{x_\mathrm{J}} \frac{C_l}{2} \dot{\psi}(x)^2 \mathrm{d}x + \int_{x_\mathrm{J}}^{l} \frac{C_l}{2} \dot{\psi}(x)^2 \mathrm{d}x + \frac{1}{2} C_\mathrm{J} (\dot{\psi}(x_\mathrm{J}^+) - \dot{\psi}(x_\mathrm{J}^-))^2, \tag{9}$$

where on the first line $\Psi_i = \int_{-\infty}^{t} V_i(t')\,\mathrm{d}t'$ denotes the node flux related to the ith capacitor in terms of the node voltage V_i, the index i assumes values from the set $\{1, \ldots, J, J+1, \ldots, N\}$, $\Delta x = 2l/N$ describes the length scale of the discretization, C_l denotes the capacitance per unit length of the CPW, and C_J denotes the junction capacitance that is much smaller than the other relevant capacitances in the circuit. The second line represents the kinetic energy in the continuum limit, $N \to \infty$, where $\Psi_i \to \psi(x_i, t)$, and x_J^- and x_J^+ correspond to the locations of the left and right electrode of the Josephson junction, respectively.

Before writing down the potential energy of the circuit, we relate the flux across the ith inductor to the external magnetic flux using Faraday's law

$$-\Phi_{\mathrm{diff},i} + \Psi_i + \Psi_{L,i} - \Psi_{i-1} = 0 \Rightarrow \Psi_{L,i} = \Psi_{i-1} - \Psi_i + \Phi_{\mathrm{diff},i}, \tag{10}$$

where $\Psi_{L,i}$ denotes the flux across the ith inductor, the index i assumes values from the set $\{1, \ldots, J, J+2, \ldots, N+1\}$, and $\Phi_{\mathrm{diff},i}$ corresponds to half of the external magnetic flux difference on the interval $x \in [x_{i-1}, x_i]$. Note that there is no inductor between the indices J and $J+1$ due to the presence of the Josephson junction. Using the above equation, we express the

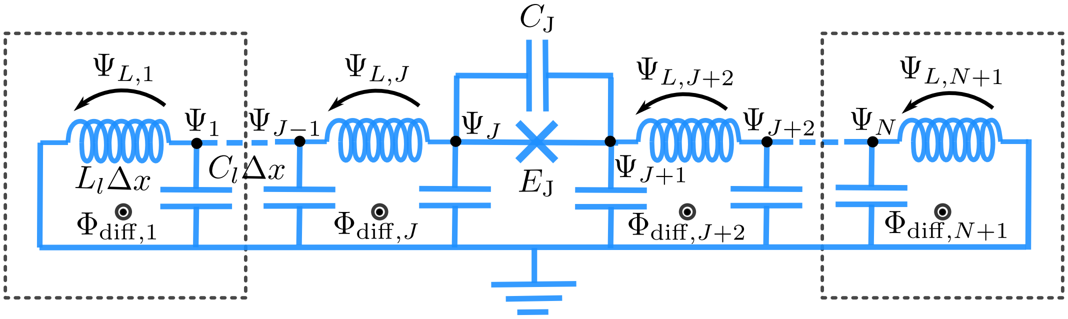

Supplementary Fig. 2: **Distributed-element circuit model of the unimon.** Discretized model of the unimon circuit in the presence of a flux bias Φ_diff. The circuit model consists of N lumped-element inductors and capacitors such that J inductors and capacitors are located on the left side of the Josephson junction and $N-J$ inductors and capacitors are located on the right side of the Josephson junction. The two boxes with dashed black contours illustrate the repeating elements of the lumped-element circuit on the left and right side of the junction. The node flux Ψ_i at the ith capacitor is defined as the time integral of the corresponding node voltage, whereas $\Psi_{L,i}$ denotes the branch flux across the ith inductor. Note that $\Phi_{\mathrm{diff},i}$ should be interpreted as the corresponding half difference of the external magnetic fluxes on the two sides of the center conductor of the CPW. A positive applied flux is defined to point out of the page, and hence to give rise to a clockwise rotating dc supercurrent.

potential energy of the system as

$$U = \frac{1}{2L_l\Delta x}\sum_{i=1,i\neq J+1}^{N+1}(\Psi_{i-1}-\Psi_i+\Phi_{\mathrm{diff},i})^2 - E_\mathrm{J}\cos\left[\frac{2\pi}{\Phi_0}(\Psi_{J+1}-\Psi_J)\right] \tag{11}$$

$$\xrightarrow[N\to\infty]{} \int_{-l}^{x_\mathrm{J}}\frac{(\partial_x\psi - sB_\mathrm{diff})^2}{2L_l}\mathrm{d}x + \int_{x_\mathrm{J}}^{l}\frac{(\partial_x\psi - sB_\mathrm{diff})^2}{2L_l}\mathrm{d}x$$
$$-E_\mathrm{J}\cos\left(\frac{2\pi}{\Phi_0}(\psi(x_\mathrm{J}^+)-\psi(x_\mathrm{J}^-))\right), \tag{12}$$

where on the first line we define $\Psi_0 = \Psi_{N+1} = 0$, L_l denotes the total inductance per unit length, and E_J denotes the Josephson energy. The second line represents the potential energy in the continuum limit, where $\Phi_\mathrm{diff,i}/\Delta x \to sB_\mathrm{diff}(x_i)$ with s being the perpendicular distance between the center conductor and the ground plane and $B_\mathrm{diff}(x_i)$ denoting half of the effective magnetic flux density difference on the two sides of the center conductor at the location x_i.

The classical equation of motion for the ith node flux Ψ_i ($i \neq J, J+1$) is obtained from the

Euler–Lagrange equation

$$\frac{\mathrm{d}}{\mathrm{d}t}\frac{\partial \mathcal{L}}{\partial \dot{\Psi}_i} - \frac{\partial \mathcal{L}}{\partial \Psi_i} = 0. \tag{13}$$

Inserting the Lagrangian in equation (7) into the Euler–Lagrange equation, we acquire

$$C_l \Delta x \ddot{\Psi}_i - \frac{1}{L_l \Delta x}\left(\Psi_{i+1} - 2\Psi_i + \Psi_{i-1} + \Phi_{\mathrm{diff},i} - \Phi_{\mathrm{diff},i+1}\right) = 0, \quad i \neq J, J+1. \tag{14}$$

By taking the the continuum limit, $\Delta x \to 0$, of the above equation of motion, we further obtain

$$C_l \ddot{\psi} = \frac{1}{L_l}\partial_{xx}\psi - \frac{s}{L_l}\partial_x B_{\mathrm{diff}}. \tag{15}$$

Note that equation (15) corresponds to a wave equation with a source term. In the following calculations, we assume that the applied magnetic field is sufficiently homogeneous in the x direction such that we can neglect the magnetic-field-dependent term from equation (15) resulting in the wave equation

$$\ddot{\psi} = v_{\mathrm{p}}^2 \partial_{xx}\psi, \tag{16}$$

where we have defined the phase velocity $v_{\mathrm{p}} = 1/\sqrt{L_l C_l}$.

Subsequently, we derive the equation of motion for the node flux Ψ_J that corresponds to the left electrode of the junction. According to the Euler–Lagrange equation, we obtain

$$C_l \Delta x \ddot{\Psi}_J + C_{\mathrm{J}}(\ddot{\Psi}_J - \ddot{\Psi}_{J+1}) + I_{\mathrm{c}} \sin\left[\frac{2\pi}{\Phi_0}(\Psi_J - \Psi_{J+1})\right] = -\frac{1}{L_l \Delta x}(\Psi_J - \Psi_{J-1} - \Phi_{\mathrm{diff},J}). \tag{17}$$

In the continuum limit, $\Delta x \to 0$, the above equation of motion can be written as

$$-C_{\mathrm{J}}\Delta\ddot{\psi} - I_{\mathrm{c}}\sin\left(\frac{2\pi}{\Phi_0}\Delta\psi\right) = -\frac{1}{L_l}\partial_x \psi\bigg|_{x=x_{\mathrm{J}}^-} + \frac{\Phi_{\mathrm{diff}}}{2lL_l}, \tag{18}$$

where $\Delta\psi = \Psi_{J+1} - \Psi_J$ denotes the change in the flux across the junction, $\Phi_{\mathrm{diff}} = \sum_i \Phi_{\mathrm{diff},i}$ is the total external half flux difference as above, and we have utilized the assumption of a homogenous magnetic field in order to write $\Phi_{\mathrm{diff},J}/\Delta x \to \Phi_{\mathrm{diff}}/(2l)$. Note that our definition of $\Delta\psi$ has a sign convention opposite to $\Psi_{L,i}$. Intuitively speaking, the above equation imposes the current continuity condition at the left electrode of the Josephson junction. The term on the left side of equation (18) can be interpreted as the total current across the Josephson junction including the charging of the junction electrodes and a supercurrent across an ideal Josephson element. The term on the right side of equation (18) corresponds to the current in the CPW resonator in the presence of an external magnetic flux as can be inferred from Eq. (10), according to which the current at location x_i in the center conductor is given by

$$I = \frac{\Psi_{i-1} - \Psi_i + \Phi_{\mathrm{diff},i}}{L_l \Delta x} \xrightarrow[N\to\infty]{} -\frac{1}{L_l}\partial_x\psi\bigg|_{x=x_i} + \frac{\Phi_{\mathrm{diff}}}{2lL_l}, \tag{19}$$

where we have again imposed the assumption of a homogeneous flux bias Φ_{diff}. Importantly, we can also derive a similar boundary condition at the right electrode of the Josephson junction

$$-C_{\mathrm{J}}\Delta\ddot{\psi} - I_{\mathrm{c}}\sin\left(\frac{2\pi}{\Phi_0}\Delta\psi\right) = -\frac{1}{L_l}\partial_x\psi\Big|_{x=x_{\mathrm{J}}^+} + \frac{\Phi_{\mathrm{diff}}}{2lL_l}. \tag{20}$$

Classically, the solution to the wave equation in equation (16) can be decomposed into a sum of oscillatory normal modes and a dc component corresponding to a spatially and temporally constant current as

$$\psi(x,t) = \phi_0 u_0(x) + \sum_{n\geq 1} u_n(x)\psi_n(t), \tag{21}$$

where the result applies for $x \neq x_{\mathrm{J}}$. In the above equation, ϕ_0 is the coefficient of the dc mode in the units of flux, and $u_0(x)$ is the corresponding dimensionless envelope function. Furthermore, $u_n(x)$ are dimensionless mode envelopes and $\psi_n(t) = c_n \exp(-\mathrm{i}\omega_n t)$ are the corresponding temporally oscillating coefficients in the units of flux with ω_n being the classical mode frequency. In order for the decomposition to make sense, the mode envelopes must be required to satisfy the wave equation in equation (16), and the boundary conditions corresponding to the grounding of the CPW and the current continuity across the Josephson junction [see equations (18) and (20)].

Importantly, the dc component of the current should behave in line with the results of the dc analysis presented in the previous section. To recover equation (6) and to comply with the grounding of the CPW, the envelope function of the dc mode must be chosen as a piece-wise linear function

$$u_0(x) = \begin{cases} (x+l)/(2l), & \text{for } x \in [-l, x_{\mathrm{J}}), \\ (x-l)/(2l), & \text{for } x \in (x_{\mathrm{J}}, l], \end{cases} \tag{22}$$

which corresponds to a spatially constant current of $I = (\Phi_{\mathrm{diff}} - \phi_0)/(2lL_l)$ based on Eq. (19) and to a dc Josephson phase difference of $\varphi_0 = 2\pi\phi_0/\Phi_0$. Importantly, we use the decomposition in equation (21) and rewrite the current continuity condition in equation (18) as

$$C_{\mathrm{J}}\sum_{n\geq 1}\omega_n^2\Delta u_n\psi_n + I_{\mathrm{c}}\sin\left[\frac{2\pi}{\Phi_0}\left(\phi_0 - \sum_{n\geq 1}\Delta u_n\psi_n\right)\right] + \overbrace{I_{\mathrm{c}}\sin\left(\frac{2\pi\phi_0}{\Phi_0}\right) - I_{\mathrm{c}}\sin\left(\frac{2\pi\phi_0}{\Phi_0}\right)}^{=0}$$
$$= -\frac{1}{L_l}\left(\frac{\phi_0}{2l} + \sum_{n\geq 1}\psi_n\partial_x u_n\Big|_{x=x_{\mathrm{J}}^-}\right) + \frac{\Phi_{\mathrm{diff}}}{2lL_l}, \tag{23}$$

where $\Delta u_n = u_n(x_\mathrm{J}^+) - u_n(x_\mathrm{J}^-)$ denotes the change in the envelope of the nth mode across the Josephson junction. The above equation can be grouped into a time-independent part and a time-dependent part that should both vanish

$$\left\{ I_\mathrm{c} \sin\left(\frac{2\pi\phi_0}{\Phi_0}\right) + \frac{\phi_0}{2lL_l} - \frac{\Phi_\mathrm{diff}}{2lL_l} \right\} + \left\{ C_\mathrm{J} \sum_{n\geq 1} \omega_n^2 \Delta u_n \psi_n \right.$$
$$\left. + I_\mathrm{c} \sin\left[\frac{2\pi}{\Phi_0}\left(\phi_0 - \sum_{n\geq 1} \Delta u_n \psi_n\right)\right] - I_\mathrm{c} \sin\left(\frac{2\pi\phi_0}{\Phi_0}\right) + \frac{1}{L_l} \sum_{n\geq 1} \psi_n \partial_x u_n\big|_{x=x_\mathrm{J}^-} \right\} = 0. \tag{24}$$

We simplify the time-dependent part further using the trigonometric identity $\sin(x+y) = \sin(x)\cos(y) + \cos(x)\sin(y)$ as

$$C_\mathrm{J} \sum_{n\geq 1} \omega_n^2 \Delta u_n \psi_n + I_\mathrm{c} \sin\left(\frac{2\pi\phi_0}{\Phi_0}\right) \cos\left(\frac{2\pi}{\Phi_0} \sum_{n\geq 1} \Delta u_n \psi_n\right)$$
$$- I_\mathrm{c} \cos\left(\frac{2\pi\phi_0}{\Phi_0}\right) \sin\left(\frac{2\pi}{\Phi_0} \sum_{n\geq 1} \Delta u_n \psi_n\right) - I_\mathrm{c} \sin\left(\frac{2\pi\phi_0}{\Phi_0}\right) + \frac{1}{L_l} \sum_{n\geq 1} \psi_n \partial_x u_n\big|_{x=x_\mathrm{J}^-} = 0. \tag{25}$$

By invoking the assumption of small ac oscillations $\left(\zeta := \sum_{n\geq 1} 2\pi \Delta u_n \psi_n / \Phi_0 \ll 1\right)$, we write the above equation into a simple form

$$\sum_{n\geq 1} \psi_n \left[C_\mathrm{J} \omega_n^2 \Delta u_n - \frac{\cos(2\pi\phi_0/\Phi_0)}{L_\mathrm{J}} \Delta u_n + \frac{1}{L_l} \partial_x u_n\big|_{x=x_\mathrm{J}^-} \right] \approx 0, \tag{26}$$

where we used the approximations $\sin(\zeta) \approx \zeta$ and $\cos(\zeta) \approx 1$ together with the relation $L_\mathrm{J} = \Phi_0/(2\pi I_\mathrm{c})$. Importantly, each term corresponding to a different n in equation (26) must vanish independently since all the coefficients $\psi_n \propto \exp(-\mathrm{i}\omega_n t)$ oscillate in time with different frequencies. Note that our assumption of small ac oscillations $\left(\sum_{n\geq 1} 2\pi \Delta u_n \psi_n / \Phi_0 \ll 1\right)$ is equivalent to linearizing the circuit around its dc operation point, which also has the consequence of decoupling the different modes of the circuit. In the following equations, we drop the approximation sign for notational simplicity.

Under the assumption of small ac oscillations, it is thus possible to summarize the current continuity conditions across the Josephson junction with the following set of decoupled equations

$$I_\mathrm{c} \sin\left(\frac{2\pi}{\Phi_0}\phi_0\right) + \frac{\phi_0}{2lL_l} = \frac{\Phi_\mathrm{diff}}{2lL_l}, \tag{27}$$

$$\omega_m^2 C_\mathrm{J} \Delta u_m - \frac{\cos(2\pi\phi_0/\Phi_0)}{L_\mathrm{J}} \Delta u_m = -\frac{1}{L_l} \partial_x u_m\big|_{x=x_\mathrm{J}^-}, \quad m \geq 1, \tag{28}$$

$$-\frac{1}{L_l} \partial_x u_m\big|_{x=x_\mathrm{J}^-} = -\frac{1}{L_l} \partial_x u_m\big|_{x=x_\mathrm{J}^+}, \quad m \geq 1, \tag{29}$$

where the final equation essentially requires current continuity on both sides of the junction and it has been obtained by also considering the current continuity condition of the right electrode in equation (20). We make a few important observations based on the above set of boundary conditions. Assuming that the ground-loop inductance is much smaller than the total inductance of the center conductor $2lL_l$, we have $L_{\mathrm{CPW}} = 2lL_l$, in the case of which equation (27) reduces to the dc flux quantization condition presented in equation (6). On the other hand, equation (28) can be interpreted such that the dc supercurrent essentially changes the phase bias of the Josephson junction by $2\pi\phi_0/\Phi_0$ resulting in an effective linear inductance of $L_{\mathrm{J}}/\cos(2\pi\phi_0/\Phi_0)$ in the limit of small oscillations. Importantly, the effective inductance of the biased Josephson junction $L_{\mathrm{J}}/\cos(2\pi\phi_0/\Phi_0)$ can be negative for certain values of the dc phase $\varphi_0 = 2\pi\phi_0/\Phi_0$. The operation points corresponding to negative inductances are classically stable with a single energy minimum if the Josephson inductance L_{J} exceeds the total inductance of the CPW $L_{\mathrm{CPW}} = 2lL_l$.

Subsequently, we use the derived set of boundary conditions to determine the normal-mode angular frequencies ω_m and the mode envelope functions $u_m(x)$. To satisfy both the wave equation (16) and the grounding condition, we use a piece-wise sinusoidal ansatz for each of the envelope functions

$$u_m(x) = \begin{cases} A_m \sin[k_m(x+l)], & \text{if } x \in [-l, x_{\mathrm{J}}) \\ A_m B_m \sin[k_m(x-l)], & \text{if } x \in (x_{\mathrm{J}}, l] \end{cases} \tag{30}$$

where k_m is the wavenumber of the mth mode obeying $k_m = \omega_m/v_{\mathrm{p}}$ in order to satisfy the wave equation, and A_m and B_m are dimensionless constants. We solve B_m by inserting the piece-wise sinusoidal ansatz of the mode envelope function to the boundary condition in equation (29), which yields

$$B_m = \frac{\cos[k_m(x_{\mathrm{J}}+l)]}{\cos[k_m(x_{\mathrm{J}}-l)]}. \tag{31}$$

By further inserting the piece-wise sinusoidal mode envelopes to the boundary condition in equation (28) and using equation (31) to carry out simplifications, we obtain a transcendental equation for the wavenumbers k_m ($m \geq 1$) of the normal modes

$$k_m l \cos[k_m(x_{\mathrm{J}}-l)] \cos[k_m(x_{\mathrm{J}}+l)] - \left[\frac{C_{\mathrm{J}}(k_m l)^2}{C_l l} - \frac{L_l l}{L_{\mathrm{J}}} \cos\left(\frac{2\pi\phi_0}{\Phi_0}\right)\right] \sin(2k_m l) = 0, \tag{32}$$

where the value of ϕ_0 should be obtained by first solving equation (27), and the wave numbers k_m are related to the classical mode angular frequencies ω_m through the relation $\omega_m = v_{\mathrm{p}} k_m$. Importantly, the mode envelope functions corresponding to solutions of the above equation may or

may not have a discontinuity ($\Delta u_m \neq 0$) at the location of the Josephson junction. The modes with a discontinuity at the junction are the modes of interest since in the following quantum-mechanical treatment, a mode can have an anharmonic energy spectrum only if the non-linear junction couples to it, i.e., a non-zero current flows across the junction.

In Supplementary Fig. **3**, we illustrate the mode envelope functions of the three normal modes with the lowest frequency at external flux bias of $\Phi_{\mathrm{diff}}/\Phi_0 = 0.0$ and $\Phi_{\mathrm{diff}}/\Phi_0 = 0.5$. The mode envelope functions at the two external fluxes differ due to a different value of the effective Josephson inductance: At $\Phi_{\mathrm{diff}}/\Phi_0 = 0.0$, the effective Josephson inductance is L_{J}, whereas at $\Phi_{\mathrm{diff}}/\Phi_0 = 0.5$, the effective inductance is $-L_{\mathrm{J}}$. Note that the lowest anharmonic mode corresponds to the second normal mode of the system at $\Phi_{\mathrm{diff}}/\Phi_0 = 0.0$, whereas the first normal mode is the lowest anharmonic mode at $\Phi_{\mathrm{diff}}/\Phi_0 = 0.5$.

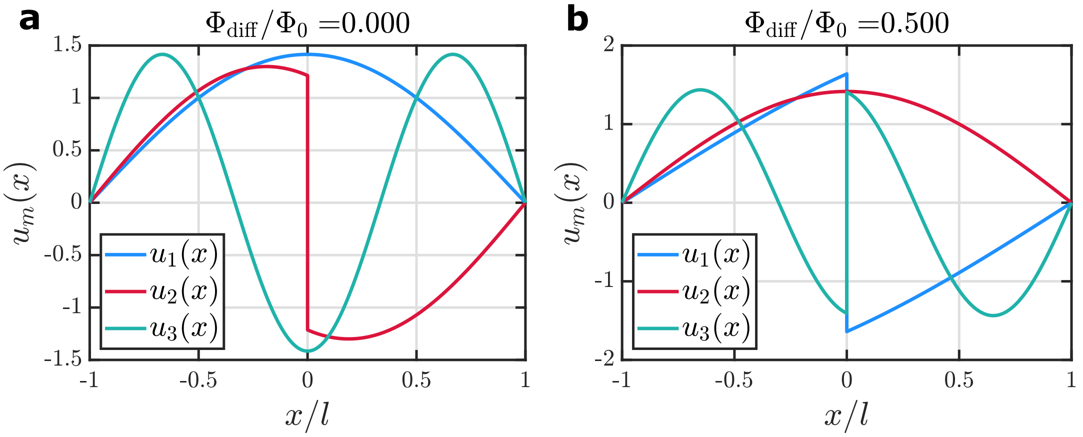

Supplementary Fig. 3: **Mode envelopes of the unimon. a**, **b**, Flux envelope functions for the three lowest-frequency normal modes at a flux bias of $\Phi_{\mathrm{diff}}/\Phi_0 = 0.0$ (**a**) and at $\Phi_{\mathrm{diff}}/\Phi_0 = 0.5$ (**b**). The results were obtained using the example parameter set presented in Supplementary Table **1**.

Importantly, the classical normal modes satisfy useful orthogonality relations[3]

$$\langle u_m, u_n \rangle = \int_{-l}^{l} C_l u_m(x) u_n(x)\,\mathrm{d}x + C_{\mathrm{J}} \Delta u_m \Delta u_n = C_\Sigma \delta_{mn}, \tag{33}$$

$$\langle \partial_x u_m, \partial_x u_n \rangle = \int_{-l}^{l} \frac{1}{L_l} \partial_x u_m(x) \partial_x u_n(x)\,\mathrm{d}x + \frac{\cos(2\pi\phi_0/\Phi_0)}{L_{\mathrm{J}}} \Delta u_m \Delta u_n = \frac{\delta_{mn}}{L_m}, \tag{34}$$

where $C_\Sigma = 2C_l l + C_{\mathrm{J}}$ denotes the total capacitance of the circuit, and the effective inductance corresponding to the mth mode is given by $L_m = (C_\Sigma \omega_m^2)^{-1}$. Note that the above orthogonality relations fix the normalization coefficients $\{A_m\}$ introduced in equation (30).

Supplementary Table 1: **Example set of parameter values used in Supplementary Methods I.** The parameter values listed in the table include the relative location of the Josephson junction x_J/l, the total length $2l$ of the center conductor in the unimon, the Josephson energy E_J and the capacitance C_J of the Josephson junction, the capacitance C_l and inductance L_l per unit length of the CPW structure, the characteristic impedance Z_0 of the CPW structure, and the ratio of the CPW inductance to the Josephson inductance $2lL_l/L_\mathrm{J}$ that is derived from the other values in the table.

x_J/l	$2l$ (mm)	E_J/h (GHz)	C_J (fF)	C_l (pF/m)	L_l (μH/m)	Z_0 (Ω)	$2lL_l/L_\mathrm{J}$
0.0	8.0	19.0	1.4	83	0.83	100	0.772

Above, we have described how to solve the classical equations of motion for the system and how to determine the frequencies and flux mode envelopes of the normal modes in the limit of small flux oscillations across the junction. In the limit of small oscillations, the classical Hamiltonian decouples to a sum of harmonic oscillators corresponding to the normal modes. Subsequently, we derive an approximation for the classical Hamiltonian of the unimon circuit by invoking a single-mode approximation, in which all other modes apart from the mode used as a qubit are neglected. The single-mode approximation describes the system with a good accuracy since the frequency difference of the anharmonic modes coupled to each other is of the order of 10 GHz in our system and consequently, the coupling between the modes has only a small perturbative effect.

Note that a more accurate expression for the Hamiltonian may be obtained by keeping more than one of the modes in the following calculations. Such a treatment is needed especially if one considers the case of several solutions to the dc flux in equation (6). Since the mode envelopes provide a complete set of basis functions for the grounded CPW system, an exact expression for the Hamiltonian can, in principle, be obtained by taking into account an infinite number of the modes.

In the single-mode approximation, we truncate the flux decomposition in equation (21) as $\psi(x,t) = \phi_0 u_0(x) + \psi_m(t)u_m(x)$, where m is the index of the anharmonic mode ($\Delta u_m \neq 0$) utilized as the qubit. Invoking the single-mode approximation and the continuum limit, the kinetic energy of the circuit in equation (8) can be expressed as

$$T = \int_{-l}^{l} \frac{C_l}{2}\dot{\psi}_m^2 u_m^2 \mathrm{d}x + \frac{C_\mathrm{J}}{2}\dot{\psi}_m^2(\Delta u_m)^2 = \frac{C_\Sigma}{2}\dot{\psi}_m^2, \tag{35}$$

where we have used the orthogonality relation in equation (33) to obtain the final result. Under

these approximations, we simplify the potential energy of the circuit in equation (11) to obtain

$$\begin{aligned} U &= \int_{-l}^{l} \frac{1}{2L_l}\left(\frac{\phi_0}{2l} + \psi_m \partial_x u_m - \frac{\Phi_{\text{diff}}}{2l}\right)^2 \mathrm{d}x - E_{\text{J}} \cos\left[\frac{2\pi}{\Phi_0}(-\phi_0 + \psi_m \Delta u_m)\right] \\ &= \frac{\psi_m^2}{2\tilde{L}_m(\phi_0)} + \frac{1}{2lL_l}\psi_m \Delta u_m (\Phi_{\text{diff}} - \phi_0) - E_{\text{J}} \cos\left[\frac{2\pi}{\Phi_0}(-\phi_0 + \psi_m \Delta u_m)\right], \end{aligned} \tag{36}$$

where we have dropped a constant term and used the orthogonality relation presented in equation (34). Furthermore, we have defined the effective inductance $\tilde{L}_m(\phi_0)$ in the last step as

$$\frac{1}{\tilde{L}_m(\phi_0)} = \frac{1}{L_m} - \frac{\cos(2\pi\phi_0/\Phi_0)}{L_{\text{J}}}(\Delta u_m)^2. \tag{37}$$

Subsequently, we define the flux variable $\phi_m = \psi_m \Delta u_m$ that equals the (time-dependent) flux difference across the Josephson junction. This allows us to write the classical single-mode Lagrangian as

$$\mathcal{L} = \frac{C'_m}{2}\dot{\phi}_m^2 - \frac{\phi_m^2}{2\tilde{L}'_m} - \frac{1}{2lL_l}\phi_m(\Phi_{\text{diff}} - \phi_0) + E_{\text{J}} \cos\left[\frac{2\pi}{\Phi_0}(\phi_m - \phi_0)\right], \tag{38}$$

where $C'_m = C_\Sigma/(\Delta u_m)^2$ and $\tilde{L}'_m = \tilde{L}_m(\Delta u_m)^2$ are the rescaled capacitance and inductance. By taking a Legendre transformation of the Lagrangian, we obtain the corresponding classical Hamiltonian

$$H_m = \frac{q_m^2}{2C'_m} + \frac{\phi_m^2}{2\tilde{L}'_m} + \frac{1}{2lL_l}\phi_m(\Phi_{\text{diff}} - \phi_0) - E_{\text{J}} \cos\left[\frac{2\pi}{\Phi_0}(\phi_m - \phi_0)\right], \tag{39}$$

where we have defined the conjugate momentum with units of charge as $q_m = \partial\mathcal{L}/\partial\dot{\phi}_m = C'_m\dot{\psi}_m$.

Finally, we obtain the quantum-mechanical single-mode Hamiltonian by imposing the canonical commutation relation $[\hat{\phi}_m, \hat{q}_m] = \mathrm{i}\hbar$. Furthermore, we define the dimensionless charge operator $\hat{n}_m = \hat{q}_m/(2e)$ and the phase operator $\hat{\varphi}_m = 2\pi\hat{\phi}_m/\Phi_0$, which allows us to express the quantum Hamiltonian in a form resembling the fluxonium Hamiltonian as

$$\hat{H}_m = 4E_{C,m}(\varphi_0)\hat{n}_m^2 + \frac{1}{2}E_{L,m}(\varphi_0)\hat{\varphi}_m^2 + E_L\hat{\varphi}_m(\varphi_{\text{diff}} - \varphi_0) - E_{\text{J}}\cos(\hat{\varphi}_m - \varphi_0), \tag{40}$$

where we have defined $E_{C,m}(\varphi_0) = e^2/[2C'_m(\varphi_0)]$, $E_{L,m}(\varphi_0) = \Phi_0^2/(2\pi)^2/\tilde{L}'_m(\varphi_0)$, $E_L = \Phi_0^2/(2\pi)^2/(2lL_l)$, $\varphi_{\text{diff}} = 2\pi\Phi_{\text{diff}}/\Phi_0$, and $\varphi_0 = 2\pi\phi_0/\Phi_0$. Note that the conjugate operators $\hat{\varphi}_m$ and $\hat{n}_m$ satisfy the commutation relation $[\hat{\varphi}_m, \hat{n}_m] = \mathrm{i}$.

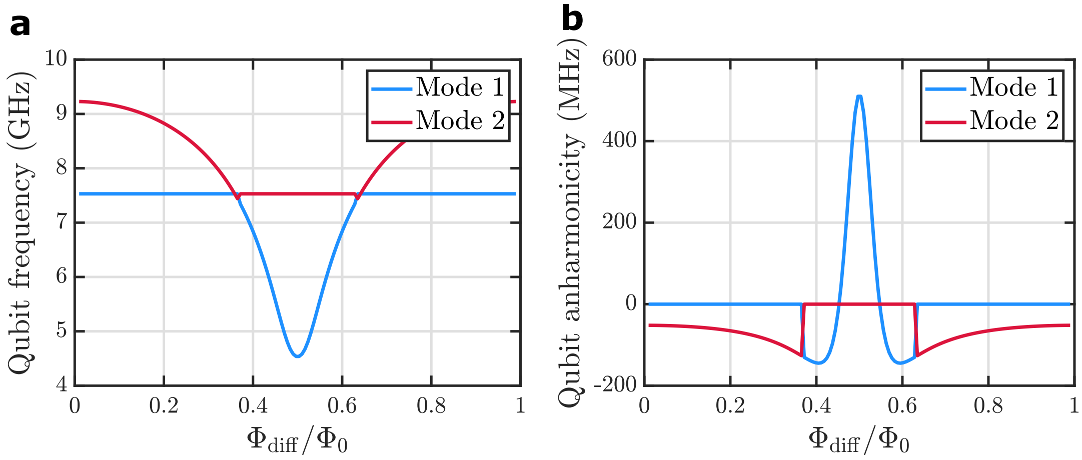

Supplementary Fig. 4: **Numerically computed frequency and anharmonicity of the unimon.** **a**, Qubit frequency $f_{01,m}$ as a function of the flux bias Φ_{diff} for the two lowest-frequency modes. **b**, Anharmonicity $\alpha_m/(2\pi)$ as a function of Φ_{diff} for the two lowest-frequency modes. The results were obtained using the example parameter set presented in Supplementary Table **1**. Note that the results were computed individually for each of the modes by enforcing the single-mode approximation and diagonalizing the Hamiltonian in equation (40).

To study the qubit frequency and anharmonicity of the unimon, we numerically diagonalize the Hamiltonian in equation (40) for flux biases in the range $\Phi_{\text{diff}}/\Phi_0 \in [0, 1]$. For each of the modes, the corresponding qubit frequency and anharmonicity are computed as

$$f_{01,m} = \frac{E_{1,m} - E_{0,m}}{h}, \tag{41}$$

$$\frac{\alpha_m}{2\pi} = \frac{(E_{2,m} - E_{1,m}) - (E_{1,m} - E_{0,m})}{h}, \tag{42}$$

where $f_{01,m}$ and $\alpha_m/(2\pi)$ denote the qubit frequency and anharmonicity corresponding to the mth mode, whereas $E_{i,m}$ is the ith eigenenergy of the mth mode. In Supplementary Fig. **4**, we illustrate the numerically computed qubit frequency and anharmonicity of the two lowest-frequency modes as functions of the flux bias. Importantly, there is a flux-insensitive sweet spot at $\Phi_{\text{diff}}/\Phi_0 = 0.5$ that corresponds to the smallest frequency and highest anharmonicity of the lowest-frequency mode. For the parameter values presented in Supplementary Table **1**, the qubit frequency at $\Phi_{\text{diff}}/\Phi_0 = 0.5$ is approximately 4.5 GHz, whereas the corresponding anharmonicity is +510 MHz. Thus, the sweet spot $\Phi_{\text{diff}}/\Phi_0 = 0.5$ corresponds to the optimal operation point of the unimon.

To gain intuitive understanding of the enhanced anharmonicity at $\Phi_{\text{diff}}/\Phi_0 = 0.5$, we note

that the single-mode Hamiltonian simplifies into

$$\hat{H}_m = 4E_{C,m}(\pi)\hat{n}_m^2 + \frac{1}{2}E_{L,m}(\pi)\hat{\varphi}_m^2 + E_\mathrm{J}\cos(\hat{\varphi}_m), \tag{43}$$

where we have used the fact that $\varphi_\mathrm{diff} = \varphi_0 = \pi$, which is valid if $E_\mathrm{J} \leq E_L$. By expanding the cosine potential as a Taylor series, we obtain

$$\hat{H}_m = 4E_{C,m}(\pi)\hat{n}_m^2 + \frac{E_{L,m}(\pi) - E_\mathrm{J}}{2}\hat{\varphi}_m^2 + \frac{E_\mathrm{J}}{24}\hat{\varphi}_m^4 + \mathcal{O}(\hat{\varphi}_m^6). \tag{44}$$

Importantly, the unimon is designed to work in the regime $E_\mathrm{J} \lesssim E_{L,m}(\pi)$. Thus, the enhanced anharmonicity can be attributed to the partial cancellation of the inductive energy $E_{L,m}(\pi)$ and the Josephson energy E_J, as result of which the quadratic potential energy term practically vanishes while the quartic and higher order terms are preserved.

Path-integral-based model for the unimon (model 2)

We start from the classical action for the electromagnetic field of the unimon, which can be expressed with the help of the temporally dependent flux density $\psi(x,t)$ in the unimon circuit based on the continuum-limit Lagrangian given by Eqs. (7), (9), (12) as

$$S[\psi(x,t)] = \int \left(\int_{-l}^{x_{\rm J}} \left\{ \frac{C_l \dot{\psi}(x,t)^2}{2} - \frac{\left[\partial_x \psi(x,t) - sB_{\rm diff}(x)\right]^2}{2L_l} \right\} {\rm d}x \right.$$
$$+ \int_{x_{\rm J}}^{l} \left\{ \frac{C_l \dot{\psi}(x,t)^2}{2} - \frac{\left[\partial_x \psi(x,t) - sB_{\rm diff}(x)\right]^2}{2L_l} \right\} {\rm d}x + \frac{C_{\rm J}\left[\dot{\psi}\left(x_{\rm J}^-,t\right) - \dot{\psi}\left(x_{\rm J}^+,t\right)\right]^2}{2}$$
$$\left. + E_{\rm J} \cos\left\{ \frac{2\pi}{\Phi_0}\left[\psi\left(x_{\rm J}^-,t\right) - \psi\left(x_{\rm J}^+,t\right)\right]\right\}\right) {\rm d}t \quad (45)$$

The spectrum of the unimon can be extracted from the partition function $Z(\beta)$, where $\beta = 1/(k_{\rm B}T)$. The partition function of the unimon can be expressed through a path integral of an exponent of an Euclidean action in the imaginary time $\tau = {\rm i}t$ as

$$Z(\beta) = \int \boldsymbol{D}[\psi(x,\tau)] e^{-\frac{1}{\hbar} S^{\rm E}[\psi(x,\tau)]}, \quad (46)$$

where the path integral is taken over all periodic trajectories in imaginary time and the Euclidean action $S^{\rm E}[\psi(x,\tau)] = S[\psi(x,-i\tau)]$ is given by

$$S^{\rm E}[\psi(x,\tau)] = \int_0^{\hbar\beta} \left(\int_{-l}^{x_{\rm J}} \left\{ \frac{C_l [\partial_\tau \psi(x,\tau)]^2}{2} + \frac{\left[\partial_x \psi(x,\tau) - sB_{\rm diff}(x)\right]^2}{2L_l} \right\} {\rm d}x \right.$$
$$+ \int_{x_{\rm J}}^{l} \left\{ \frac{C_l [\partial_\tau \psi(x,\tau)]^2}{2} + \frac{\left[\partial_x \psi(x,\tau) - sB_{\rm diff}(x)\right]^2}{2L_l} \right\} {\rm d}x$$
$$\left. + \frac{C_{\rm J}\left[\partial_\tau \psi(x_{\rm J}^-,\tau) - \partial_\tau \psi(x_{\rm J}^+,\tau)\right]^2}{2} - E_{\rm J}\cos\left\{\frac{2\pi}{\Phi_0}\left[\psi(x_{\rm J}^-,\tau) - \psi(x_{\rm J}^+,\tau)\right]\right\}\right) {\rm d}\tau, \quad (47)$$

where the interval of imaginary time is determined by the inverse temperature β. First, we define

$\psi'(x,\tau) = \psi(x,\tau) - \Phi_{\mathrm{diff}}(x)$, where

$$\Phi_{\mathrm{diff}}(x) = s \begin{cases} \displaystyle\int_{-l}^{x} B_{\mathrm{diff}}(x')\,\mathrm{d}x',\ x < x_{\mathrm{J}} \\ \displaystyle -\int_{x}^{l} B_{\mathrm{diff}}(x')\,\mathrm{d}x',\ x > x_{\mathrm{J}}. \end{cases} \tag{48}$$

Then the action takes the following form:

$$S^{\mathrm{E}}[\psi'(x,\tau)] = \int_0^{\hbar\beta} \left(\int_{-l}^{x_{\mathrm{J}}} \left\{ \frac{C_l[\partial_\tau \psi'(x,\tau)]^2}{2} + \frac{\left[\partial_x \psi'(x,\tau)\right]^2}{2L_l} \right\} \mathrm{d}x \right.$$
$$+ \int_{x_{\mathrm{J}}}^{l} \left\{ \frac{C_l[\partial_\tau \psi'(x,\tau)]^2}{2} - \frac{\left[\partial_x \psi'(x,\tau)\right]^2}{2L_l} \right\} \mathrm{d}x + \frac{C_{\mathrm{J}}\left[\partial_\tau \psi'(x_{\mathrm{J}}^-,\tau) - \partial_\tau \psi'(x_{\mathrm{J}}^+,\tau)\right]^2}{2}$$
$$\left. - E_{\mathrm{J}} \cos\left\{ \frac{2\pi}{\Phi_0} \left[\psi'(x_{\mathrm{J}}^-,\tau) - \psi'(x_{\mathrm{J}}^+,\tau) + \Phi_{\mathrm{diff}} \right] \right\} \right) \mathrm{d}\tau, \tag{49}$$

where $\Phi_{\mathrm{diff}} = \Phi_{\mathrm{diff}}(x_{\mathrm{J}}^-) - \Phi_{\mathrm{diff}}(x_{\mathrm{J}}^+)$.

In the following derivation, we eliminate all the linear resonator degrees of freedom and arrive at a model expressed in terms of a single variable, namely phase difference across the junction. To this end, we seek for fixed $\psi'(x_{\mathrm{J}}^-,\tau)$ and $\psi'(x_{\mathrm{J}}^+,\tau)$ classical trajectories which minimize the Euclidean action of the linear resonator parts of the system. We first focus on the left half of the resonator with $x \in [-l, x_{\mathrm{J}})$ and then adjust the results for the right side of the resonator. The equation for the classical trajectory $\psi_{\mathrm{c}}(x,\tau)$ corresponds to the Euler–Lagrange equation for the Euclidean action that is given as

$$\partial_\tau^2 \psi_{\mathrm{c}}(x,\tau) + v_{\mathrm{p}}^2 \partial_x^2 \psi_{\mathrm{c}}(x,\tau) = 0 \tag{50}$$

where $v_{\mathrm{p}}^2 = 1/(L_l C_l)$. Since we integrate only over the periodic in imaginary time trajectories with the fixed value of the flux at the junction, we impose the following boundary conditions:

$$\psi_{\mathrm{c}}(-l,\tau) = 0,\ \text{for}\ \tau \in [0,\hbar\beta], \tag{51}$$

$$\psi_{\mathrm{c}}(x_{\mathrm{J}},\tau) = \psi'(x_{\mathrm{J}}^-,\tau),\ \text{for}\ \tau \in [0,\hbar\beta], \tag{52}$$

$$\psi_{\mathrm{c}}(x,0) = \psi_{\mathrm{c}}(x,\hbar\beta),\ \text{for}\ x \in [-l, x_{\mathrm{J}}]. \tag{53}$$

We expand the flux at the junction into a Fourier series as

$$\psi'(x_{\rm J}^{-},\tau)=\frac{1}{\hbar\beta}\sum_{n=-\infty}^{\infty}\psi'_n(x_{\rm J}^{-})e^{-{\rm i}\omega_n\tau}, \tag{54}$$

where $\omega_n=2\pi nk_{\rm B}T/\hbar$ are the bosonic Matsubara frequencies. Consequently, the classical trajectory is given by the following expression:

$$\psi_{\rm c}(x,\tau)=\frac{1}{\hbar\beta}\sum_{n=-\infty}^{\infty}\psi'_n(x_{\rm J}^{-})\frac{\sinh\left[\frac{\omega_n}{v_{\rm p}}(x+l)\right]}{\sinh\left[\frac{\omega_n}{v_{\rm p}}(x_{\rm J}+l)\right]}e^{-i\omega_n\tau}. \tag{55}$$

We make a substitution $\psi'(x,\tau)=\psi_{\rm c}(x,\tau)+\tilde{\psi}(x,\tau)$ in the path integral expression for the partition function. Thus the action for the Euclidean left half of the resonator reads as

$$\begin{aligned}&S_{\rm l}^{\rm E}\left[\tilde{\psi}(x,\tau),\psi'(x_{\rm J}^{-},\tau)\right]=\\ &\int_0^{\hbar\beta}\int_{-l}^{x_{\rm J}}\left\{\frac{C_l[\partial_\tau\psi_{\rm c}(x,\tau)]^2}{2}+\frac{[\partial_x\psi_{\rm c}(x,\tau)]^2}{2L_l}+\frac{C_l[\partial_\tau\tilde{\psi}(x,\tau)]^2}{2}+\frac{[\partial_x\tilde{\psi}(x,\tau)]^2}{2L_l}\right\}\,{\rm d}x\,{\rm d}\tau=\\ &\int_{-l}^{x_{\rm J}}\left\{\frac{C_l[\partial_\tau\tilde{\psi}(x,\tau)]^2}{2}+\frac{[\partial_x\tilde{\psi}(x,\tau)]^2}{2L_l}\right\}\,{\rm d}x\,{\rm d}\tau+\frac{1}{\hbar\beta}\sum_{n=-\infty}^{\infty}|\psi'_n(x_{\rm J}^{-})|^2\frac{\omega_n}{2Z\tanh\left(\frac{\omega_n}{v_p}l_{\rm l}\right)},\end{aligned} \tag{56}$$

where $Z=\sqrt{L_l/C_l}$ and $l_{\rm l}=l+x_{\rm J}$. Repeating the above procedure for the right half of the resonator, we obtain the following expression for Euclidean action of the whole unimon with $\psi'(x_{\rm J}^{-},\tau)$ and $\psi'(x_{\rm J}^{+},\tau)$ given:

$$\begin{aligned}&S^{\rm E}\left[\tilde{\psi}(x,\tau),\psi'(x_{\rm J}^{-},\tau),\psi'(x_{\rm J}^{+},\tau)\right]=\int_0^{\hbar\beta}\left(\int_{-l}^{x_{\rm J}}\left\{\frac{C_l\left[\partial_\tau\tilde{\psi}(x,\tau)\right]^2}{2}+\frac{\left[\partial_x\tilde{\psi}(x,\tau)\right]^2}{2L_l}\right\}\,{\rm d}x\right.\\ &+\int_{x_{\rm J}}^{l}\left\{\frac{C_l\left[\partial_\tau\tilde{\psi}(x,\tau)\right]^2}{2}+\frac{\left[\partial_x\tilde{\psi}(x,\tau)\right]^2}{2L_l}\right\}\,{\rm d}x+\frac{C_{\rm J}\left[\partial_\tau\psi'(x_{\rm J}^{-},\tau)-\partial_\tau\psi'(x_{\rm J}^{+},\tau)\right]^2}{2}\\ &\left.-E_{\rm J}\cos\left\{\frac{2\pi}{\Phi_0}\left[\psi'(x_{\rm J}^{-},\tau)-\psi'(x_{\rm J}^{+},\tau)+\Phi_{\rm diff}\right]\right\}\right)\,{\rm d}\tau+\frac{1}{\hbar\beta}\sum_{n=-\infty}^{+\infty}\frac{\omega_n}{2Z}\left[\frac{\left|\psi'_n(x_{\rm j}^{-})\right|^2}{\tanh\left(\frac{\omega_n}{v_{\rm p}}l_{\rm l}\right)}+\frac{\left|\psi'_n(x_{\rm j}^{+})\right|^2}{\tanh\left(\frac{\omega_n}{v_{\rm p}}l_{\rm r}\right)}\right],\end{aligned} \tag{57}$$

where $l_{\mathrm{r}} = l - x_{\mathrm{J}}$. The partition function is expressed through this action as

$$Z(\beta) = \int \boldsymbol{D} \left[\tilde{\psi}(x,\tau), \psi'(x_{\mathrm{J}}^-,\tau), \psi'(x_{\mathrm{J}}^+,\tau)\right] e^{-\frac{1}{\hbar} S^{\mathrm{E}}\left[\tilde{\psi}(x,\tau),\psi'(x_{\mathrm{J}}^-,\tau),\psi'(x_{\mathrm{J}}^+,\tau)\right]}. \tag{58}$$

The field $\tilde{\psi}(x,\tau)$ is uncoupled from $\psi'(x_{\mathrm{J}}^{\pm},\tau)$ since the boundary conditions of the total flux $\psi'(x,\tau) = \psi_{\mathrm{c}}(x,\tau) + \tilde{\psi}(x,\tau)$ are obeyed by the classical part according to equations (51)–(53) and consequently the field $\tilde{\psi}(x,\tau)$ has zero boundary conditions independent of $\psi'(x_{\mathrm{J}}^{\pm},\tau)$. Thus the field $\tilde{\psi}(x,\tau)$ can be integrated out. Since it satisfies zero boundary conditions at $x=-l$, $x=x_{\mathrm{J}}$, and $x=l$, it is equal to a product of partition functions of two grounded $\lambda/2$ resonators with lengths l_{l} and l_{r}. Consequently, we have

$$Z(\beta) = Z'(\beta) \int \boldsymbol{D}[\psi'(x_{\mathrm{J}}^-,\tau), \psi'(x_{\mathrm{J}}^+,\tau)] e^{-\frac{1}{\hbar} S_{\mathrm{J}}^{\mathrm{E}}[\psi'(x_{\mathrm{J}}^-,\tau),\psi'(x_{\mathrm{J}}^+,\tau)]}, \tag{59}$$

where

$$Z'(\beta) = \prod_{m=1}^{\infty} \frac{1}{\left(1 - e^{-\hbar\beta \frac{m v_{\mathrm{p}}}{l_{\mathrm{l}}}}\right)\left(1 - e^{-\hbar\beta \frac{m v_{\mathrm{p}}}{l_{\mathrm{r}}}}\right)}, \tag{60}$$

$$S_{\mathrm{J}}^{\mathrm{E}}[\psi'(x_{\mathrm{J}}^-,\tau), \psi'(x_{\mathrm{J}}^+,\tau)] = \int_0^{\hbar\beta} \left(\frac{C_{\mathrm{J}} \left[\partial_\tau \psi'(x_{\mathrm{J}}^-,\tau) - \partial_\tau \psi'(x_{\mathrm{J}}^+,\tau)\right]^2}{2} \right.$$
$$\left. - E_{\mathrm{J}} \cos\left\{ \frac{2\pi}{\Phi_0} \left[\psi'(x_{\mathrm{J}}^-,\tau) - \psi'(x_{\mathrm{J}}^+,\tau) + \Phi_{\mathrm{diff}}\right] \right\} \right) \mathrm{d}\tau + \frac{1}{\hbar\beta} \sum_{n=-\infty}^{+\infty} \left[\left|\psi_n'(x_{\mathrm{J}}^-)\right|^2 K_{\mathrm{l}n} + \left|\psi_n'(x_{\mathrm{J}}^+)\right|^2 K_{\mathrm{r}n} \right], \tag{61}$$

and

$$K_{\alpha n} = \frac{\omega_n}{2Z \tanh\left(\frac{\omega_n}{v_{\mathrm{p}}} l_\alpha\right)}, \text{ for } \alpha \in \{\mathrm{r}, \mathrm{l}\}. \tag{62}$$

Here, we introduce

$$\psi_+(\tau) = \frac{1}{2}\left[\psi'(x_{\mathrm{J}}^-,\tau) + \psi'(x_{\mathrm{J}}^+,\tau)\right],$$
$$\psi_-(\tau) = \psi'(x_{\mathrm{J}}^-,\tau) - \psi'(x_{\mathrm{J}}^+,\tau),$$

and

$$K_{\pm,n} = K_{\mathrm{l}n} \pm K_{\mathrm{r}n},$$

and express the corresponding Euclidean action in terms of these variables as

$$S_{\mathrm{J}}^{\mathrm{E}}[\psi_+(\tau),\psi_-(\tau)] = \int\limits_0^{\hbar\beta} \left(\frac{C_{\mathrm{J}}\left[\partial_\tau \psi_-(\tau)\right]^2}{2} - E_{\mathrm{J}} \cos\left\{ \frac{2\pi}{\Phi_0}\left[\psi_-(\tau)+\Phi_{\mathrm{diff}}\right]\right\}\right) \mathrm{d}\tau +$$

$$\frac{1}{\hbar\beta}\sum_{n=-\infty}^{+\infty}\left[\left|\psi_{+,n}+\frac{1}{2}K_{+,n}^{-1}K_{-,n}\psi_{-,n}\right|^2 K_{+,n}+\frac{1}{4}\left(K_{+,n}-K_{-,n}^2K_{+,n}^{-1}\right)|\psi_{-,n}|^2\right], \quad (63)$$

where $\psi_{\pm,n}$ are the Fourier components of $\psi_\pm(\tau)$ in the spirit of equation (54). We proceed by introducing $\psi'_{+,n}=\psi_{+,n}+\frac{1}{2}K_{+,n}^{-1}K_{-,n}\psi_{-,n}$ and integrate out the ψ'_+ degree of freedom to obtain

$$Z(\beta) = Z'(\beta)Z_+(\beta)\int \boldsymbol{D}[\psi_-(\tau)]e^{-\frac{1}{\hbar}S_-^{\mathrm{E}}[\psi_-(\tau)]}, \quad (64)$$

where

$$Z_+(\beta) = \lim_{M\to\infty}\prod_{n=-M}^{M}\frac{\mathcal{N}_M}{\sqrt{K_{+,n}}}, \quad (65)$$

where $\mathcal{N}_M$ are normalization constants needed to enforce the convergence of the product. Thus, we obtain

$$S_-^{\mathrm{E}}[\psi_-(\tau)] = \int\limits_0^{\hbar\beta}\left\{\frac{C_{\mathrm{J}}\left[\partial_\tau\psi_-(\tau)\right]^2}{2} - E_{\mathrm{J}}\cos\left[\frac{2\pi}{\Phi_0}\left(\psi_-(\tau)+\Phi_{\mathrm{diff}}\right)\right]\right\}\mathrm{d}\tau + \frac{1}{\hbar\beta}\sum_{n=-\infty}^{+\infty}\frac{1}{4}K_n|\psi_{-,n}|^2, \quad (66)$$

where

$$K_n = K_{+,n}-K_{-,n}^2K_{+,n}^{-1} = \frac{2\omega_n}{Z\left[\tanh\left(\frac{\omega_n}{v_{\mathrm{p}}}l_{\mathrm{l}}\right)+\tanh\left(\frac{\omega_n}{v_{\mathrm{p}}}l_{\mathrm{r}}\right)\right]}. \quad (67)$$

Until this point for model 2, we have not applied any approximations, i.e., the expression for the action $S_-^{\mathrm{E}}[\psi_-(\tau)]$ is exact. However, this action is challenging to utilize further in analytical calculations since it is both non-Gaussian and non-local in imaginary time. The corresponding low-temperature partition function is determined by the low-Matsubara-frequency contribution of the non-local kernel K_n. To proceed, we expand this kernel in the vicinity of $\omega_n=0$ as

$$K_n \approx \frac{1}{lL_l}+\frac{C_l(l^2+3x_{\mathrm{J}}^2)}{3l}\omega_n^2. \quad (68)$$

These terms correspond to an effective capacitance and inductance, induced by the resonator. Consequently, we write a non-Gaussian action that is local in imaginary time as

$$S_0^{\mathrm{E}}[\psi_-(\tau)] = \int\limits_0^{\hbar\beta}\left(\frac{C_{\mathrm{eff}}\left[\partial_\tau\psi_-(\tau)\right]^2}{2}+\frac{\psi_-^2(\tau)}{2L_{\mathrm{eff}}} - E_{\mathrm{J}}\cos\left\{\frac{2\pi}{\Phi_0}\left[\psi_-(\tau)+\Phi_{\mathrm{diff}}\right]\right\}\right)\mathrm{d}\tau, \quad (69)$$

where

$$C_{\text{eff}} = C_{\text{J}} + \frac{C_l(l^2 + 3x_{\text{J}}^2)}{6l}, \tag{70}$$

$$L_{\text{eff}} = 2lL_l. \tag{71}$$

Partition function, corresponding to this action, can be evaluated numerically by diagonalization of the following Hamiltonian:

$$\hat{H}_0 = \frac{\hat{Q}_-^2}{2C_{\text{eff}}} + \frac{\hat{\psi}_-^2}{2L_{\text{eff}}} - E_{\text{J}} \cos\left[\frac{2\pi}{\Phi_0}\left(\hat{\psi}_- + \Phi_{\text{diff}}\right)\right], \tag{72}$$

where $[\hat{\psi}_-, \hat{Q}_-] = \mathrm{i}\hbar$. This Hamiltonian has indentical form to that of the lumped-element unimon and can be used to obtain a qualitative spectrum of unimon. However it is too inaccurate for any quantitative analysis so we need to develop a better approximation.

To proceed, we rewrite the non-local kernel (67) as follows:

$$K_n(\omega_n) = \frac{\mathrm{i}\omega_n \left[\cos\left(\frac{2\mathrm{i}\omega_n}{v_{\text{p}}} x_{\text{J}}\right) + \cos\left(\frac{2\mathrm{i}\omega_n}{v_{\text{p}}} l\right)\right]}{Z \sin\left(\frac{2\mathrm{i}\omega_n}{v_{\text{p}}} l\right)}. \tag{73}$$

Importantly, $K_n(\omega_n)$ has poles at frequencies of the $\lambda/2$ resonator modes $\omega_n = \pm \mathrm{i}\pi k v_{\text{p}}/(2l)$, $k \in \mathbb{Z}_+$. The residues of these poles are equal to $\pm \mathrm{i}\pi k v_{\text{p}}^2/(4l^2 Z)\left[(-1)^k \cos\left(\frac{\pi k x_{\text{J}}}{l}\right) + 1\right]$. We approximate the resonator with a set of M auxiliary modes with frequencies $\Omega_k = \pi k v_{\text{p}}/(2l)$, inductively coupled to the non-linear oscillator described by ψ_- degree of freedom. Thus we consider a trial action of the form

$$S^{\text{E}}_{\text{trial}} = \int_0^{\hbar\beta} \left(\frac{C[\partial_\tau \psi_-(\tau)]^2}{2} + \frac{\psi_-^2(\tau)}{2L_\psi} - E_{\text{J}} \cos\left\{\frac{2\pi}{\Phi_0}\left[\psi_-(\tau) + \Phi_{\text{diff}}\right]\right\} \right.$$
$$\left. + \sum_{k=1}^{M} \left\{ \frac{C[\partial_t \chi_k(\tau)]^2}{2} + \frac{C\Omega_k^2 \chi_k^2(\tau)}{2} + \alpha_k \chi_k(\tau)\psi_-(\tau) \right\} \right) \mathrm{d}\tau =$$
$$\int_0^{\hbar\beta} \left(\frac{C[\partial_\tau \psi_-(\tau)]^2}{2} + \frac{\psi_-^2(\tau)}{2L_\psi} - E_{\text{J}} \cos\left\{\frac{2\pi}{\Phi_0}\left[\psi_-(\tau) + \Phi_{\text{diff}}\right]\right\} \right) \mathrm{d}\tau +$$
$$\frac{1}{\hbar\beta} \sum_{k=1}^{M} \sum_{n=-\infty}^{\infty} \left[\frac{C}{2}\left(\omega_n^2 + \Omega_k^2\right) \left| \chi_{k,n} + \frac{\alpha_k \psi_{-,n}}{C(\omega_n^2 + \Omega_k^2)} \right|^2 - \frac{\alpha_k^2 |\psi_{-,n}|^2}{2C(\omega_n^2 + \Omega_k^2)} \right], \tag{74}$$

where $\{\chi_k\}_{k=1}^M$ are the flux variables of the auxiliary modes, $\chi_{k,n}$ is the nth Fourier coefficient of $\chi_k(\tau)$, and C, L_ψ, and α_k are coefficients used below to match the trial action with that in equation (66). To this end, we define $\chi'_{k,n} = \chi_{k,n} + \alpha_k/[(C(\omega_n^2 + \Omega_k^2)]\psi_{-,n}$ and integrate out variables $\chi'_{k,n}$. We obtain an effective action for the variable ψ_- as

$$S_M^{\rm E} = \int_0^{\hbar\beta} \left(\frac{C[\partial_\tau \psi_-(\tau)]^2}{2} + \frac{\psi_-^2(\tau)}{2L_\psi} - E_{\rm J} \cos\left\{ \frac{2\pi}{\Phi_0} \left[\psi_-(\tau) + \Phi_{\rm diff} \right] \right\} \right) {\rm d}\tau \\ - \frac{1}{\hbar\beta} \sum_{n=-\infty}^{+\infty} \sum_{k=1}^{M} \frac{\alpha_k^2 |\psi_{-,n}|^2}{2C(\omega_n^2 + \Omega_k^2)}. \quad (75)$$

The kernel of non-local part of the action also has poles in the frequency domain at $\omega_n = \pm {\rm i}\Omega_k$. The residues of the kernel at these poles are equal to $\pm {\rm i}\alpha_k^2/(4C\Omega_k)$. Thus it is natural to equate the residues of the exact kernel K_n to the residues of this trial kernel as

$$\frac{\alpha_k^2}{4C\Omega_k} = \frac{\Omega_k^2}{4\pi k Z} \left[(-1)^k \cos\left(\frac{\pi k x_{\rm J}}{l} \right) + 1 \right]. \quad (76)$$

We have two more free parameters, namely, C and L_ψ which we choose for the second-order Taylor expansion of the trial kernel to match with that of the exact kernel given in equation (68). Consequently, we have

$$\frac{1}{2L_\psi} - \sum_{k=1}^{M} \frac{\alpha_k^2}{2C\Omega_k^2} = \frac{1}{4lL_l}, \quad (77)$$

$$\frac{C}{2} + \sum_{k=1}^{M} \frac{\alpha_k^2}{2C\Omega_k^4} = \frac{C_{\rm J}}{2} + \frac{C_l(l^2 + 3x_{\rm J}^2)}{12l}. \quad (78)$$

Finally, the spectrum of the unimon is given by the Hamiltonian

$$\hat{H}_M = \frac{\hat{Q}_-^2}{2C} + \frac{\hat{\psi}_-^2}{2L_\psi} - E_{\rm J} \cos\left[\frac{2\pi}{\Phi_0} \left(\hat{\psi}_- + \Phi_{\rm diff} \right) \right] + \sum_{k=1}^{M} \left(\frac{\hat{q}_k^2}{2C} + \frac{C\Omega_k^2 \hat{\chi}_k^2}{2} + \alpha_k \hat{\chi}_k \hat{\psi}_- \right), \quad (79)$$

where $[\hat{\chi}_k, \hat{q}_m] = {\rm i}\hbar\delta_{km}$, $[\hat{\psi}_-, \hat{Q}_-] = {\rm i}\hbar$, and all other single-operator commutators are zero.

In model 2 of this work, we choose $M = 2$, i.e., we consider two lowest auxiliary modes. Thus in general, we need to solve a 3D Schrödinger equation which is feasible with the modern computational techniques. Note that if the unimon is symmetric ($x_{\rm J} = 0$), the first mode is decoupled from the junction, i.e., $\alpha_1 = 0$. However, the second mode is strongly coupled to the junction, and hence it cannot be neglected or treated perturbatively. In this case, the problem is reduced to solving a 2D Schrödinger equation which is computationally convenient.

Supplementary Methods II: Derivation of the theoretical model for a coupled unimon-resonator system

Hamiltonian for the coupled unimon-resonator system

Here, we derive the Hamiltonian for a coupled system consisting of a unimon qubit and a $\lambda/4$ readout resonator using an approach extended from the theoretical model 1 presented in Supplementary Methods I. In the derivation, we take into account the qubit mode of the unimon and the lowest-frequency mode of the readout resonator. See Supplementary Fig. **5** for a schematic illustration of the coupled system and a lumped-element circuit model of the coupling.

We begin by writing the Lagrangians of the different parts of the coupled system. Referring to equation (38), we write the classical Lagrangian owing to the bare qubit mode m of the unimon as

$$\mathcal{L}_{\rm u} = \frac{C_m'}{2}\dot{\phi}_m^2 - \frac{\phi_m^2}{2\tilde{L}_m'} - \frac{1}{2lL_l}\phi_m(\Phi_{\rm diff} - \phi_0) + E_{\rm J}\cos[2\pi(\phi_m - \phi_0)/\Phi_0], \tag{80}$$

where as above C_m' is the effective capacitance of the qubit mode m, ϕ_m is the flux across the Josephson junction owing to the mth mode, $\tilde{L}_m'$ is the effective inductance of the qubit mode, $2l$ is the length of the unimon, $E_{\rm J}$ is the Josephson energy, ϕ_0 is the flux across the Josephson junction owing to the dc component, L_l is the inductance per unit length of the unimon, and $\Phi_{\rm diff}$ is the half of the external flux difference. In the following, we assume that the coupling to the readout resonator does not affect the mode envelope functions of the unimon.

To write the Lagrangian related to the capacitive coupling, we note that the flux at the coupling location $x_{\rm g}$ is related to the flux across the Josephson junction as

$$\phi_{m,{\rm g}} = \phi_m u_m(x_{\rm g})/(\Delta u_m), \tag{81}$$

where $u_m(x_{\rm g})$ is the envelope function of the mth mode evaluated at the coupling location $x_{\rm g}$ and Δu_m is the discontinuity in the envelope function $\Delta u_m = u_m(x_{\rm J}^+) - u_m(x_{\rm J}^-)$ across the junction. Thus, the Lagrangian corresponding to the capacitive coupling between the unimon and the resonator (see Supplementary Fig. **5**) can be written as

$$\mathcal{L}_{\rm g} = \frac{1}{2}C_{\rm g}\left(\dot{\phi}_m\frac{u_m(x_{\rm g})}{\Delta u_m} - \dot{\phi}_{\rm r}\right)^2, \tag{82}$$

where $\phi_{\rm r}$ is the node flux of the readout resonator at the coupling location. Next, the Lagrangian of the resonator is expressed as

$$\mathcal{L}_{\rm r} = \frac{1}{2}C_{\rm r}\dot{\phi}_{\rm r}^2 - \frac{\phi_{\rm r}^2}{2L_{\rm r}}, \tag{83}$$

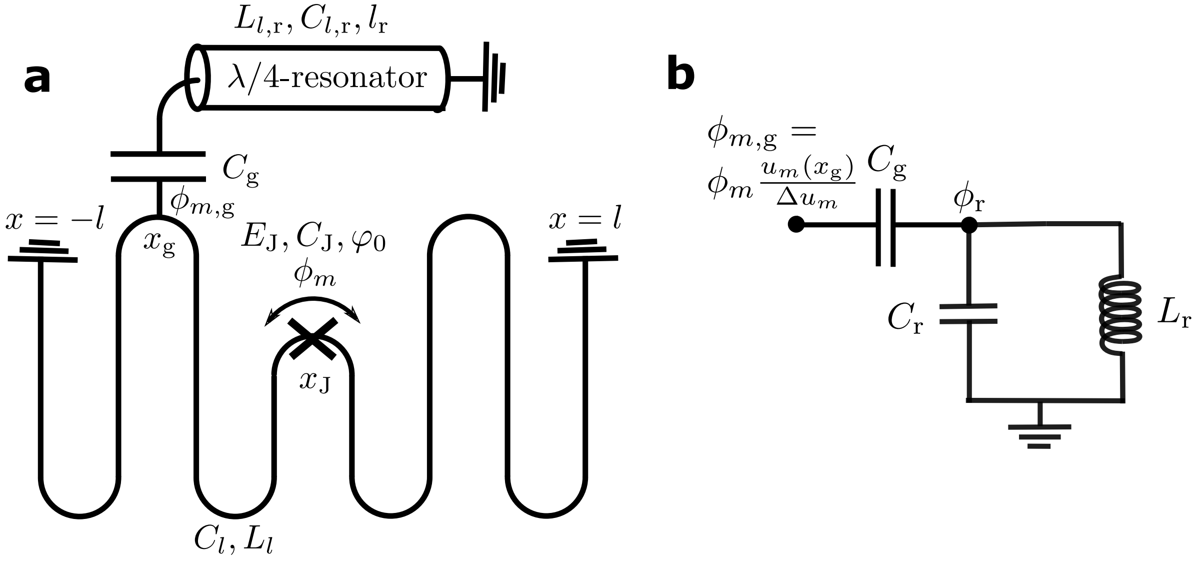

Supplementary Fig. 5: **Schematic diagram of a coupled unimon-resonator system.** **a**, Schematic illustration of a coupled system consisting of a unimon qubit and $\lambda/4$ readout resonator together with symbols corresponding to the relevant circuit parameters. Here, $2l$ is the total length of the unimon circuit, x_J is the junction location, E_J is the Josephson energy, C_J is the capacitance of the junction, φ_0 is the dc Josephson phase, ϕ_m is the flux difference across the junction owing to the qubit mode m, x_g is the location of the coupling capacitance, C_g is the coupling capacitance between the unimon and its readout resonator, $\phi_{m,\mathrm{g}}$ is the flux of the unimon center conductor at the coupling location, $L_{l,\mathrm{r}}$ is the inductance per unit length of the readout resonator, $C_{l,\mathrm{r}}$ is the capacitance per unit length of the readout resonator, and l_r is the length of the readout resonator. **b**, Circuit diagram corresponding to the coupling capacitance and the lumped-element approximation of the readout resonator in a coupled unimon-resonator system. Here, ϕ_r is the flux at the end of the readout resonator, and C_r and L_r are the effective capacitance and inductance of the readout resonator.

where C_r and L_r are the effective capacitance and inductance of the readout resonator. For a $\lambda/4$ readout resonator, we have $C_\mathrm{r} = C_{l,\mathrm{r}} l_\mathrm{r}/2$, and $L_\mathrm{r} = 8L_{l,\mathrm{r}} l_\mathrm{r}/\pi^2$, where $C_{l,\mathrm{r}}$ and $L_{l,\mathrm{r}}$ are the capacitance and inductance per unit length of the readout resonator and l_r is the length of the readout resonator. Note also that the characteristic impedance of the lumped-element resonator mode is $Z_\mathrm{r} = \sqrt{L_\mathrm{r}/C_\mathrm{r}} = 4Z_\mathrm{tr}/\pi$, where $Z_\mathrm{tr} = \sqrt{L_{l,\mathrm{r}}/C_{l,\mathrm{r}}}$. Thus, the total Lagrangian of the qubit-resonator system is given by

$$\mathcal{L} = \mathcal{L}_\mathrm{u} + \mathcal{L}_\mathrm{g} + \mathcal{L}_\mathrm{r}. \tag{84}$$

To derive the Hamiltonian of the circuit, we first write the kinetic part of the total Lagrangian in a matrix form as

$$T = \frac{1}{2}\begin{pmatrix}\dot{\phi}_m & \dot{\phi}_\mathrm{r}\end{pmatrix}\begin{pmatrix} C'_m + C_\mathrm{g}\frac{u_m(x_\mathrm{g})^2}{(\Delta u_m)^2} & -C_\mathrm{g}\frac{u_m(x_\mathrm{g})}{\Delta u_m} \\ -C_\mathrm{g}\frac{u_m(x_\mathrm{g})}{\Delta u_m} & C_\mathrm{r}+C_\mathrm{g}\end{pmatrix}\begin{pmatrix}\dot{\phi}_m \\ \dot{\phi}_\mathrm{r}\end{pmatrix} = \frac{1}{2}\dot{\boldsymbol{\phi}}^T\boldsymbol{C}\dot{\boldsymbol{\phi}}, \tag{85}$$

where $\boldsymbol{\phi} = (\phi_m, \phi_\mathrm{r})^T$ is a node flux vector and $\boldsymbol{C}$ is the corresponding capacitance matrix. With the help of the matrix notation, we can express the conjugate charges in terms of the node fluxes as

$$\boldsymbol{q} = \frac{\partial \mathcal{L}}{\partial \dot{\boldsymbol{\phi}}} = \boldsymbol{C}\dot{\boldsymbol{\phi}}, \tag{86}$$

where $\boldsymbol{q} = (q_m, q_\mathrm{r})^T$ is a vector containing the conjugate charges. Using the matrix notation, the classical Hamiltonian can be written as

$$H = \dot{\boldsymbol{\phi}}^T\frac{\partial \mathcal{L}}{\partial \dot{\boldsymbol{\phi}}} - \mathcal{L} = \frac{1}{2}\boldsymbol{q}^T\boldsymbol{C}^{-1}\boldsymbol{q} + U(\boldsymbol{\phi}). \tag{87}$$

We can approximate the matrix elements of $\boldsymbol{C}^{-1}$ as

$$\boldsymbol{C}^{-1}(1,1) = \frac{C_\mathrm{g}+C_\mathrm{r}}{\left[C'_m + C_\mathrm{g}\frac{u_m(x_\mathrm{g})^2}{(\Delta u_m)^2}\right](C_\mathrm{r}+C_\mathrm{g}) - C_\mathrm{g}^2\frac{u_m(x_\mathrm{g})^2}{(\Delta u_m)^2}} \approx \frac{1}{C'_m + C_\mathrm{g}\frac{u_m(x_\mathrm{g})^2}{(\Delta u_m)^2}}, \tag{88}$$

$$\boldsymbol{C}^{-1}(1,2) = \frac{C_\mathrm{g}\frac{u_m(x_\mathrm{g})}{\Delta u_m}}{\left[C'_m + C_\mathrm{g}\frac{u_m(x_\mathrm{g})^2}{(\Delta u_m)^2}\right](C_\mathrm{r}+C_\mathrm{g}) - C_\mathrm{g}^2\frac{u_m(x_\mathrm{g})^2}{(\Delta u_m)^2}} \approx \frac{C_\mathrm{g}\frac{u_m(x_\mathrm{g})}{\Delta u_m}}{\left[C'_m + C_\mathrm{g}\frac{u_m(x_\mathrm{g})^2}{(\Delta u_m)^2}\right](C_\mathrm{r}+C_\mathrm{g})}, \tag{89}$$

$$\boldsymbol{C}^{-1}(2,2) = \frac{C'_m + C_\mathrm{g}\frac{u_m(x_\mathrm{g})^2}{(\Delta u_m)^2}}{\left[C'_m + C_\mathrm{g}\frac{u_m(x_\mathrm{g})^2}{(\Delta u_m)^2}\right](C_\mathrm{r}+C_\mathrm{g}) - C_\mathrm{g}^2\frac{u_m(x_\mathrm{g})^2}{(\Delta u_m)^2}} \approx \frac{1}{C_\mathrm{r}+C_\mathrm{g}}, \tag{90}$$

where we have used the valid assumption that the coupling capacitance C_g is much smaller than the effective unimon capacitance C'_m and the resonator capacitance C_r. Inserting these approximations to the Hamiltonian in equation (87), we obtain

$$\begin{aligned} H = &\left\{\frac{q_m^2(\Delta u_m)^2}{2C_\mathrm{u,tot}} + \frac{\phi_m^2}{2\tilde{L}'_m} + \frac{1}{2lL_l}\phi_m(\Phi_\mathrm{diff}-\phi_0) - E_\mathrm{J}\cos[2\pi(\phi_m-\phi_0)/\Phi_0]\right\} \\ &+ \left\{\frac{q_m q_\mathrm{r} C_\mathrm{g}\frac{u_m(x_\mathrm{g})}{\Delta u_m}}{\frac{C_\mathrm{u,tot}}{(\Delta u_m)^2}C_\mathrm{r,tot}}\right\} + \left\{\frac{q_\mathrm{r}^2}{2C_\mathrm{r,tot}} + \frac{\phi_\mathrm{r}^2}{2L_\mathrm{r}}\right\}, \end{aligned} \tag{91}$$

where we have defined the total capacitance of the unimon as $C_\mathrm{u,tot} = C'_m(\Delta u_m)^2 + C_\mathrm{g}u_m(x_\mathrm{g})^2 = 2C_l l + C_\mathrm{J} + C_\mathrm{g}u_m(x_\mathrm{g})^2$ and the total capacitance of the readout resonator as $C_\mathrm{r,tot} = C_\mathrm{r} + C_\mathrm{g}$.

In the above equation, the first curly brackets correspond to the unimon, the second curly brackets correspond to the coupling, and the third curly brackets correspond to the readout resonator. Finally, we quantize the above Hamiltonian to obtain

$$\hat{H} = \left\{4E_{C,m}^{\rm tot}\hat{n}_m^2 + \frac{1}{2}E_{L,m}\hat{\varphi}_m^2 + E_L\hat{\varphi}_m(\varphi_{\rm diff} - \varphi_0) - E_{\rm J}\cos(\hat{\varphi}_m - \varphi_0)\right\}$$
$$+ \left\{\frac{4e^2C_{\rm g}u_m(x_{\rm g})\Delta u_m}{C_{u,\rm tot}C_{\rm r,tot}}\hat{n}_m\hat{n}_{\rm r}\right\} + \left\{4E_{C,\rm r}^{\rm tot}\hat{n}_{\rm r}^2 + \frac{1}{2}E_{L,\rm r}\hat{\varphi}_{\rm r}^2\right\}, \tag{92}$$

where we have defined the charge operators as $\hat{n}_i = \hat{q}_i/(2e)$ and the phase operators as $\hat{\varphi}_i = 2\pi\hat{\phi}_i/\Phi_0$ for $i \in \{m, \rm r\}$. The charge and phase operators satisfy the usual commutation relation $[\hat{\varphi}_i, \hat{n}_j] = {\rm i}\delta_{ij}$. Furthermore, we have defined the following energy scales for the mth mode of the unimon $E_{C,m}^{\rm tot} = e^2\Delta u_m^2/(2C_{\rm u,tot})$, $E_{L,m} = \Phi_0^2/(2\pi)^2/\tilde{L}'_m$, and $E_L = \Phi_0^2/(2\pi)^2/(2lL_l)$. For the readout resonator, we have defined the energy scales $E_{C,\rm r}^{\rm tot} = e^2/(2C_{\rm r,tot})$, and $E_{L,\rm r} = \Phi_0^2/(2\pi)^2/L_{\rm r}$.

It is instructive to express the Hamiltonian in equation (92) using the eigenbasis of the unimon, and the annihilation and creation operators of the resonator. The unimon part of the Hamiltonian can be diagonalized, e.g., in the phase basis to obtain the eigenenergies $\{\hbar\omega_j\}$ and eigenstates $\{|j\rangle\}$ of the bare unimon. When it comes to the resonator, the annihilation and creation operators are related to the charge and phase operators $\hat{n}_{\rm r}$ and $\hat{\varphi}_{\rm r}$ as

$$\hat{n}_{\rm r} = -\frac{\rm i}{2}\left(\frac{E_{L,\rm r}}{2E_{C,\rm r}^{\rm tot}}\right)^{1/4}(\hat{a}_{\rm r} - \hat{a}_{\rm r}^\dagger), \tag{93}$$

$$\hat{\varphi}_{\rm r} = \left(\frac{2E_{C,\rm r}^{\rm tot}}{E_{L,\rm r}}\right)^{1/4}(\hat{a}_{\rm r} + \hat{a}_{\rm r}^\dagger), \tag{94}$$

which allows us to express the resonator Hamiltonian as

$$\hat{H}_{\rm r} = \hbar\omega_{\rm r}\hat{a}_{\rm r}^\dagger\hat{a}_{\rm r}, \tag{95}$$

where $\omega_{\rm r} = \sqrt{8E_{C,\rm r}^{\rm tot}E_{L,\rm r}}/\hbar$.

Using equations (92)–(95), the Hamiltonian of the coupled unimon-resonator system can be written as

$$\hat{H} = \hbar\omega_{\rm r}\hat{a}_{\rm r}^\dagger\hat{a}_{\rm r} + \sum_j \hbar\omega_j|j\rangle\langle j| - 2{\rm i}e^2\frac{C_{\rm g}u_m(x_{\rm g})\Delta u_m}{C_{\rm u,tot}C_{\rm r,tot}}\left(\frac{E_{L,\rm r}}{2E_{C,\rm r}^{\rm tot}}\right)^{1/4}\hat{n}_m(\hat{a}_{\rm r} - \hat{a}_{\rm r}^\dagger). \tag{96}$$

In the eigenbasis of the unimon, we can express the charge operator $\hat{n}_m$ further as

$$\hat{n}_m = -\mathrm{i}\sum_{i,j}\langle i|\mathrm{i}\hat{n}_m|j\rangle|i\rangle\langle j| = -\mathrm{i}\sum_{i,j} n_{ij}|i\rangle\langle j|, \tag{97}$$

where we have defined $n_{ij} = \langle i|\mathrm{i}\hat{n}_m|j\rangle$. If the eigenenergies and states of the unimon have been solved in the phase basis, the matrix elements can be evaluated as

$$n_{ij} = \int_{-\infty}^{\infty} \Psi_i^*(\varphi)\frac{\partial}{\partial\varphi}\Psi_j(\varphi)\mathrm{d}\varphi, \tag{98}$$

where $\Psi_j(\varphi) = \langle\varphi|j\rangle$ is the jth eigenstate of the unimon in the phase basis. Note also that $n_{ij}^* = \langle i|\mathrm{i}\hat{n}_m|j\rangle^\dagger = -\langle j|\mathrm{i}\hat{n}_m|i\rangle = -n_{ji}$.

The qubit–resonator coupling term in the Hamiltonian can be simplified as

$$\begin{aligned}
\hat{H}_\mathrm{c} &= -2\mathrm{i}e^2\frac{C_\mathrm{g}u_m(x_\mathrm{g})\Delta u_m}{C_\mathrm{u,tot}C_\mathrm{r,tot}}\left(\frac{E_{L,\mathrm{r}}}{2E_{C,\mathrm{r}}^\mathrm{tot}}\right)^{1/4}\left(-\mathrm{i}\sum_{i,j} n_{ij}|i\rangle\langle j|\right)(\hat{a}_\mathrm{r} - \hat{a}_\mathrm{r}^\dagger)\\
&= 2e^2\frac{C_\mathrm{g}u_m(x_\mathrm{g})\Delta u_m}{C_\mathrm{u,tot}C_\mathrm{r,tot}}\left(\frac{E_{L,\mathrm{r}}}{2E_{C,\mathrm{r}}^\mathrm{tot}}\right)^{1/4}\sum_{i,j}\left(n_{ij}|i\rangle\langle j|\hat{a}_\mathrm{r}^\dagger + n_{ij}^*|j\rangle\langle i|\hat{a}_\mathrm{r}\right)\\
&= \hbar\sum_{i,j}\left(g_{ij}|i\rangle\langle j|\hat{a}_\mathrm{r}^\dagger + g_{ij}^*|j\rangle\langle i|\hat{a}_\mathrm{r}\right),
\end{aligned} \tag{99}$$

where the coupling strength g_{ij} is given by

$$\begin{aligned}
g_{ij} &= \frac{2e^2}{\hbar}\frac{C_\mathrm{g}u_m(x_\mathrm{g})\Delta u_m}{C_\mathrm{u,tot}C_\mathrm{r,tot}}\left(\frac{E_{L,\mathrm{r}}}{2E_{C,\mathrm{r}}^\mathrm{tot}}\right)^{1/4} n_{ij}\\
&= 2\omega_\mathrm{r}\frac{C_\mathrm{g}u_m(x_\mathrm{g})\Delta u_m}{C_\mathrm{u,tot}}\sqrt{\frac{Z_\mathrm{r,tot}\pi}{R_K}}n_{ij} &(100)\\
&\approx 2\omega_\mathrm{r}\frac{C_\mathrm{g}u_m(x_\mathrm{g})\Delta u_m}{C_\mathrm{u,tot}}\sqrt{\frac{4Z_\mathrm{tr}}{R_K}}n_{ij}, &(101)
\end{aligned}$$

where $R_K = h/e^2$ is the von Klitzing constant and $Z_\mathrm{r,tot} = \sqrt{L_\mathrm{r}/C_\mathrm{r,tot}}$ is the effective impedance of the resonator. In the simplifications, we have used the equation

$$\left(\frac{E_{L,\mathrm{r}}}{2E_{C,\mathrm{r}}^\mathrm{tot}}\right)^{1/4}\frac{1}{C_\mathrm{r,tot}} = \frac{1}{2\sqrt{\pi}}\sqrt{R_K Z_\mathrm{r,tot}}\omega_\mathrm{r}, \tag{102}$$

and noted that $Z_\mathrm{r,tot} \approx Z_\mathrm{r} = 4Z_\mathrm{tr}/\pi$ for a $\lambda/4$-readout resonator.

After all these simplifications, the Hamiltonian of the coupled system in equation (96) can be expressed as

$$\hat{H} = \hbar\omega_{\mathrm{r}}\hat{a}_{\mathrm{r}}^{\dagger}\hat{a}_{\mathrm{r}} + \sum_{j}\hbar\omega_{j}|j\rangle\langle j| + \hbar\sum_{i,j}\left(g_{ij}|i\rangle\langle j|\hat{a}_{\mathrm{r}}^{\dagger} + g_{ij}^{*}|j\rangle\langle i|\hat{a}_{\mathrm{r}}\right). \tag{103}$$

Approximation of the Hamiltonian in the dispersive limit

In this section, we derive an approximation for the coupled Hamiltonian in the dispersive regime ($|\omega_1 - \omega_0 - \omega_{\mathrm{r}}| \gg g_{01}$), which allows us to obtain an equation for the dispersive shift of the readout resonator coupled to a unimon. Importantly, the Hamiltonian of the coupled system in equation (103) is of identical form to the Hamiltonian used in Appendix B of Ref.[4] in the derivation of the dispersive approximation for a general qubit-resonator Hamiltonian. Thus, we apply the results of Ref.[4], and obtain the following dispersive approximation of the coupled Hamiltonian

$$\hat{H}_{\mathrm{disp}} \approx \hbar\omega_{\mathrm{r}}\hat{a}_{\mathrm{r}}^{\dagger}\hat{a}_{\mathrm{r}} + \sum_{j}\hbar(\omega_j + \Lambda_j)|j\rangle\langle j| + \sum_{j}\hbar\chi_j\hat{a}_{\mathrm{r}}^{\dagger}\hat{a}_{\mathrm{r}}|j\rangle\langle j|, \tag{104}$$

where

$$\Lambda_j = \sum_{i=0}^{\infty}\chi_{ij}, \tag{105}$$

$$\chi_j = \sum_{i=0}^{\infty}(\chi_{ij} - \chi_{ji}), \tag{106}$$

with

$$\chi_{ij} = \frac{|g_{ij}|^2}{\omega_j - \omega_i - \omega_{\mathrm{r}}}. \tag{107}$$

If the dispersive Hamiltonian in equation (104) is projected to the lowest two levels of the qubit, the Hamiltonian can be further simplified as

$$\hat{H}_{\mathrm{disp}} \approx \hbar\omega_{\mathrm{r}}'\hat{a}_{\mathrm{r}}^{\dagger}\hat{a}_{\mathrm{r}} - \frac{\hbar\omega_{01}'}{2}\hat{\sigma}_z - \hbar\chi\hat{a}_{\mathrm{r}}^{\dagger}\hat{a}_{\mathrm{r}}\hat{\sigma}_z, \tag{108}$$

where we have defined $\omega_{\mathrm{r}}' = \omega_{\mathrm{r}} + (\chi_0 + \chi_1)/2$, $\hat{\sigma}_z = |0\rangle\langle 0| - |1\rangle\langle 1|$, $\omega_{01}' = \omega_1 - \omega_0 + \Lambda_1 - \Lambda_0$, and the dispersive shift is given by

$$\chi = (\chi_1 - \chi_0)/2. \tag{109}$$

According to the dispersive Hamiltonian in equation (108), the frequency of the readout resonator attains a frequency shift of $\pm\chi$ depending on whether the unimon is in the ground state or in the

excited state. Thus, we can use dispersive readout similarly to the conventional transmon systems in order to measure the state of the qubit.

Let us then derive a useful approximate expression for the dispersive shift at $\Phi_{\text{diff}}/\Phi_0 = 0.5$. At this sweet spot, the symmetry of the wave functions ensures that $g_{i,i+2k} = 0 = n_{i,i+2k}$, where $k \in \mathbb{Z}$. Furthermore, the parameter regime of the unimon ensures that $|g_{i,i+1}| \gg |g_{i,i+2k+1}|$, where $k \geq 1$. Thus, it is a reasonably accurate approximation to only take into account the coupling terms of the form $g_{i,i+1}$ or $g_{i+1,i}$ and neglect the rest of the terms. Within this approximation, we can simplify the equation of the dispersive shift by noting that

$$\chi_0 = \sum_{i=0}^{\infty}(\chi_{i0} - \chi_{0i}) \approx \chi_{10} - \chi_{01}, \tag{110}$$

and

$$\chi_1 = \sum_{i=0}^{\infty}(\chi_{i1} - \chi_{1i}) \approx \chi_{01} - \chi_{10} + \chi_{21} - \chi_{12}. \tag{111}$$

By inserting these results to equation (109), we obtain

$$\begin{aligned}\chi &\approx \frac{\chi_{01} - \chi_{10} + \chi_{21} - \chi_{12} - (\chi_{10} - \chi_{01})}{2} \\ &= |g_{01}|^2 \left(\frac{1}{\omega_{01} - \omega_{\mathrm{r}}} - \frac{-1}{\omega_{01} + \omega_{\mathrm{r}}}\right) + \frac{|g_{12}|^2}{2}\left(\frac{-1}{\omega_{12} + \omega_{\mathrm{r}}} - \frac{1}{\omega_{12} - \omega_{\mathrm{r}}}\right) \\ &\approx \frac{|g_{01}|^2}{\omega_{01} - \omega_{\mathrm{r}}} - \frac{1}{2}\frac{|g_{12}|^2}{\omega_{12} - \omega_{\mathrm{r}}} = \chi_{01} - \frac{1}{2}\chi_{12},\end{aligned} \tag{112}$$

where we have discarded small terms inversely proportional to the sum of two angular frequencies. Within this approximation, the equation for the dispersive shift is similar to that typically used for transmons[4] apart from the fact that the equation for g_{ij} is different for the unimon.

As illustrated in Supplementary Fig. **6**, we compare the experimentally measured dispersive shifts $\chi/(2\pi)$ to theoretical predictions based on equations (109) and (112). This allows us to make the following observations: The best agreement with the experimental results is obtained with the approximation in equation (112) and the experimentally measured transition frequencies $\omega_{01}/(2\pi)$ and $\omega_{12}/(2\pi)$, in the case of which the theoretical predictions and experimental measurements agree within 0–15% for qubits B–E and within 32% for qubit A. Secondly, we observe that the theoretically predicted dispersive shift is overestimated if we use numerically computed transition frequencies $\omega_{01}/(2\pi)$ and $\omega_{12}/(2\pi)$ based on the fitted circuit parameters presented in Table 1 of the main text. This arises from the fact that the theoretically predicted anharmonicity is slightly

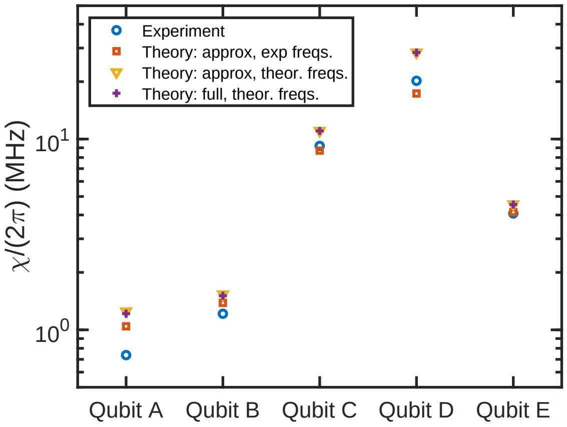

Supplementary Fig. 6: **Measured and theoretical dispersive shifts.** Measured dispersive shifts (blue circles) for the five qubits characterized in this work together with theoretical predictions. The orange squares show the theoretical prediction based on the approximation in equation (112) and experimentally measured frequencies ω_{01} and ω_{12}. The yellow triangles show the theoretical prediction corresponding to the approximation in equation (112) and numerically computed frequencies ω_{01} and ω_{12} based on the Hamiltonian in equation (40) and the fitted circuit parameters presented in Table 1 of the main text. The violet crosses present the theoretical prediction corresponding to equation (109) and numerically computed frequencies ω_{01} and ω_{12} based on the Hamiltonian in equation (40) and the fitted circuit parameters presented in Table 1 of the main text. For all of the theoretical predictions, we use a coupling capacitance C_{g} estimated from the avoided crossing of the unimon and the resonator.

higher than the experimentally measured anharmonicity as can be seen from Fig. 3(a) of the main text. Furthermore, we observe that the theoretical predictions based on equation (109) and the approximation in equation (112) agree within a few percent, thus validating the approximation in equation (112).

Supplementary Methods III: Theoretical models for the relaxation rate of the unimon

In this section, we provide models for estimating the relaxation rate of a unimon qubit due to different noise sources. If a qubit is only susceptible to a noise source λ, the T_1 decay rate of the qubit can be described with an expression that is of the Fermi golden rule type[5]

$$\Gamma_1 = \frac{1}{T_1} = \frac{|\langle 0|\partial \hat{H}_m/\partial\lambda|1\rangle|^2}{\hbar^2} S_\lambda(\omega_{01}), \tag{113}$$

where $S_\lambda(\omega_{01})$ is the symmetrized noise power spectral density of the variable λ at the qubit frequency ω_{01}. The symmetrized noise power spectral density is defined as[5]

$$S_\lambda(\omega) = \int_{-\infty}^{\infty} \mathrm{d}t(\mathrm{e}^{+\mathrm{i}\omega t} + \mathrm{e}^{-\mathrm{i}\omega t})\langle\lambda(t)\lambda(0)\rangle, \tag{114}$$

where $\langle\lambda(t)\lambda(0)\rangle$ denotes an ensemble average over the noise realizations. In the calculations below, we use the Hamiltonian $\hat{H}_m$ in Eq. (40) or its extensions, which means that that we employ the theoretical model 1 derived in Supplementary Methods I. Below, we consider relaxation due to flux noise (ohmic and $1/f$), dielectric losses, inductive losses, radiative losses, and Purcell decay through the readout resonator. In the case where many uncorrelated noise sources are relevant for the qubit, the T_1 decay rates of each noise source simply add such that the T_1 of the qubit is the inverse of the sum of all different decay rates.

Flux noise

In the case of the unimon, flux noise is caused by fluctuations of the external flux difference. Fluctuations of the external flux difference can be caused by current fluctuations in the flux line or environmental flux noise. Current fluctuations in the resistive flux line can be described with an ohmic power spectral density, whereas the environmental flux noise is often well-described by $1/f$-type noise in superconducting circuits[6].

Using equation (40), we evaluate the matrix element $\langle 0|\partial\hat{H}_m/\partial\Phi_{\mathrm{diff}}|1\rangle$ needed to compute the relaxation rate due to flux noise as

$$\langle 0|\partial\hat{H}_m/\partial\Phi_{\mathrm{diff}}|1\rangle = \frac{2\pi E_L}{\Phi_0}\langle 0|\hat{\varphi}_m|1\rangle. \tag{115}$$

Note that the relaxation is caused by fluctuations at the qubit frequency and therefore, the dc Josephson phase φ_0 can be taken to be a constant.

To estimate the relaxation rate due to ohmic flux noise, we note that the current in the flux line is related to the external flux bias according to $\Phi_{\mathrm{diff}} = MI$, where M is the mutual inductance

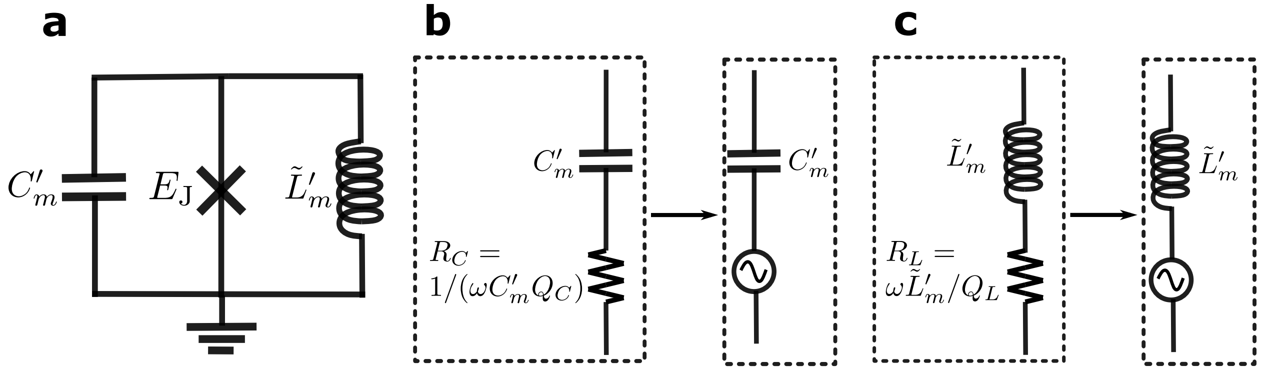

Supplementary Fig. 7: **Circuit models for relaxation rate models.** **a**, Lumped-element circuit diagram of the unimon. **b**, Lossy capacitor can be modeled with an ideal capacitor in series with an effective resistance. **c**, Lossy inductor can be modeled with an inductor in series with a resistance. In (**b**) and (**c**), fluctuations in the voltage across the effective resistance can result in the relaxation of the qubit.

of the flux coupling. In this case, the noise power spectral density of $\Phi_{\rm diff}$ can be written as

$$S_{\Phi_{\rm diff}}(\omega) = M^2 S_I(\omega) = \frac{2M^2\hbar\omega}{R}\coth\left(\frac{\hbar\omega}{2k_{\rm B}T}\right), \tag{116}$$

where R is the resistance of the flux line, $k_{\rm B}$ is the Boltzmann constant, T is temperature of the resistance, and we have utilized the noise power spectral density for the current in a resistor based on the derivation of Ref.[5]. By combining equations (115) and (116), we obtain the following equation for the relaxation rate due to Ohmic flux noise

$$\Gamma_1^M = \frac{8\pi^2 E_L^2 M^2 \omega_{01}}{\Phi_0^2\hbar R}|\langle 0|\hat{\varphi}_m|1\rangle|^2\coth\left(\frac{\hbar\omega_{01}}{2k_{\rm B}T}\right). \tag{117}$$

For the $1/f$ noise, the noise power spectral density is given by

$$S_\Phi(\omega) = \frac{2\pi A^2_{\Phi_{\rm diff}}}{\omega}, \tag{118}$$

where the constant $A_{\Phi_{\rm diff}}$ yields the noise power spectral density at 1 Hz. By combining equations (115) and (118), we obtain the following equation for the relaxation rate due to $1/f$ flux noise

$$\Gamma_1^{1/f} = 8\pi^3\frac{E_L^2}{\hbar^2}\frac{A^2_{\Phi_{\rm diff}}}{\Phi_0^2}\frac{|\langle 0|\hat{\varphi}_m|1\rangle|^2}{\omega_{01}}. \tag{119}$$

Dielectric losses

To estimate the relaxation rate due to dielectric losses, we assume that the capacitance C'_m in the

lumped-element circuit model of the unimon [see Supplementary Fig. **7**(a)] suffers from losses. These losses can be modeled with a series resistance $R_C = 1/(\omega C'_m Q_C)$ corresponding to a fixed quality factor of Q_C as illustrated in Supplementary Fig. **7**(b). Due to voltage fluctuations of the effective resistance R_C, an additional term is introduced to the Hamiltonian of the unimon such that the Hamiltonian becomes

$$\hat{H}'_m = \hat{H}_m + 2eV_{R_C}\hat{n}_m, \tag{120}$$

where V_{R_C} denotes the voltage across the effective resistance. We estimate the matrix element $\langle 0|\partial \hat{H}'_m/\partial V_{R_C}|1\rangle$ and obtain

$$\langle 0|\partial \hat{H}'_m/\partial V_{R_C}|1\rangle = 2e\langle 0|\hat{n}_m|1\rangle. \tag{121}$$

By inserting equation (121) into equation (113), we acquire that the relaxation rate owing to dielectric losses is given as

$$\Gamma_1^{\mathrm{cap}} = \frac{16E_{C,m}}{\hbar Q_C}|\langle 0|\hat{n}_m|1\rangle|^2 \coth\left(\frac{\hbar\omega_{01}}{2k_{\mathrm{B}}T}\right), \tag{122}$$

where we have used the fact that the noise power spectral density of the voltage across the effective resistor can be written as

$$S_{V_R}(\omega) = \frac{2\hbar}{Q_C C'_m}\coth\left(\frac{\hbar\omega}{2k_{\mathrm{B}}T}\right). \tag{123}$$

Note that equation (122) resembles the dielectric loss rate of fluxonium qubits [7,8].

Inductive losses

To model inductive losses, we assume that the inductor $\tilde{L}'_m$ in the lumped-element circuit model of the unimon is connected in series with a resistance $R_C = \omega\tilde{L}'_m/Q_L$ as illustrated in Supplementary Fig. **7**(c). Here, the inductor is assumed to yield a fixed quality factor of Q_L. Due to the presence of the series resistance, the quadratic potential energy associated with $\tilde{L}'_m$ is modified into the form $1/(2\tilde{L}'_m)(\hat{\phi}_m - \phi_R)^2$, where ϕ_R denotes the fluctuating flux across the series resistance. As a result, we need to evaluate the matrix element $\langle 0|\partial \hat{H}_m/\partial \phi_{R_L}|1\rangle$, which gives us

$$\langle 0|\partial \hat{H}_m/\partial \phi_R|1\rangle = \frac{\Phi_0}{2\pi\tilde{L}'_m}\langle 0|\hat{\varphi}_m|1\rangle. \tag{124}$$

The noise power spectral density for fluctuations of ϕ_R can be evaluated as[9]

$$S_{\phi_R}(\omega) = \frac{1}{\omega^2}S_{V_R}(\omega) = \frac{2\hbar\tilde{L}'_m}{Q_L}\coth\left(\frac{\hbar\omega}{2k_{\mathrm{B}}T}\right), \tag{125}$$

where V_R denotes the voltage across the effective resistor. By combining equations (124) and (125), we obtain the following equation for the relaxation rate due to inductive losses

$$\Gamma_1^{\rm ind} = \frac{2E_{L,m}}{\hbar Q_L}|\langle 0|\hat{\varphi}_m|1\rangle|^2 \coth\left(\frac{\hbar\omega_{01}}{2k_{\rm B}T}\right), \tag{126}$$

which resembles the inductive relaxation rate of fluxonium qubits [7,8].

Radiative losses

The capacitive coupling to the drive line can also give rise to relaxation due to radiative losses. We can modify an expression of the radiative relaxation rate for a fluxonium[8] to obtain the following result for the unimon

$$\Gamma_1^{\rm rad} = \frac{\omega_{01}}{Q_{\rm rad}} \coth\left(\frac{\hbar\omega_{01}}{2k_{\rm B}T}\right) |\langle 0|\hat{n}_m|1\rangle|^2, \tag{127}$$

where $Q_{\rm rad}$ is an effective quality factor associated with the relaxation to the drive line.

Purcell decay to the readout resonator

To estimate the Purcell decay to the readout resonator, we note that the Hamiltonian of a coupled unimon-resonator system in equation (103) is similar to the Hamiltonian of a coupled transmon-resonator system apart from different $\{\omega_j\}$ and $\{g_{ij}\}$. Thus, we can approximate the Purcell decay rate at a low temperature using a result similar to transmon qubits[10]

$$\Gamma_1^{\rm P} = \kappa\frac{|g_{01}|^2}{(\omega_{01}-\omega_{\rm r})^2}, \tag{128}$$

where $\kappa/(2\pi)$ is the linewidth of the readout resonator in Hz.

Comparing frequency dependence of measured T_1 to theoretical models

In Supplementary Fig. **8**, we compare the measured T_1 of the qubit B to theoretical predictions based on equations (116), (118), (122), (126), (127), and (128). We have scaled the theoretically predicted relaxation times, apart from the Purcell decay, to coincide with the experimental data at $\Phi_{\rm diff}/\Phi_0 = 0.5$ in order to compare, which relaxation mechanism could explain the measured data. Based on our analysis, the dielectric losses and Purcell decay appear to be the dominating loss mechanisms for the qubit B. When considering the dielectric loss as the dominating loss mechanism, a good fit to the data is obtained with a quality factor of $Q_C = 3.5\times 10^5$.

Note that our analysis assumes that the quality factors Q_L, Q_C, and $Q_{\rm rad}$ are independent of the qubit frequency, which may be violated in practice. Namely, the flux mode envelope functions

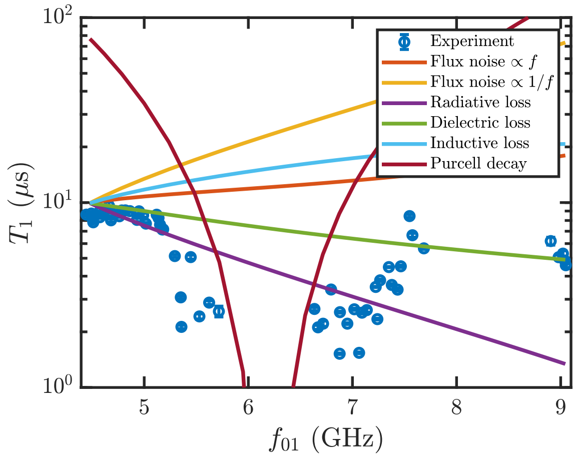

Supplementary Fig. 8: **Measured T_1 together with theoretical models.** Measured T_1 (blue circles) for qubit B as a function of qubit frequency together with theoretical predictions based on taking into account a single decay channel at a time as indicated. The theoretical predictions are based on the following results: ohmic flux noise in equation (116), $1/f$ flux noise in equation (118), dielectric losses in equation (122), inductive losses in equation (126), radiative losses in equation (127) and Purcell decay in equation (128). The theoretically predicted relaxation times, apart from the Purcell decay, were scaled to coincide with the experimental data at $\Phi_{\text{diff}}/\Phi_0 = 0.5$ corresponding to $f_{01} \approx 4.5$ GHz.

of the unimons are frequency-dependent, and thus, the electric field strengths in different parts of the circuit vary with the qubit frequency.

Supplementary Methods IV: More detailed schematic of the experimental setup

Supplementary Figure **9** presents a detailed schematic illustration of the experimental setup used in the measurements of the unimon qubit devices studied in this work. See Methods in the main article text for a discussion of the experimental setup.

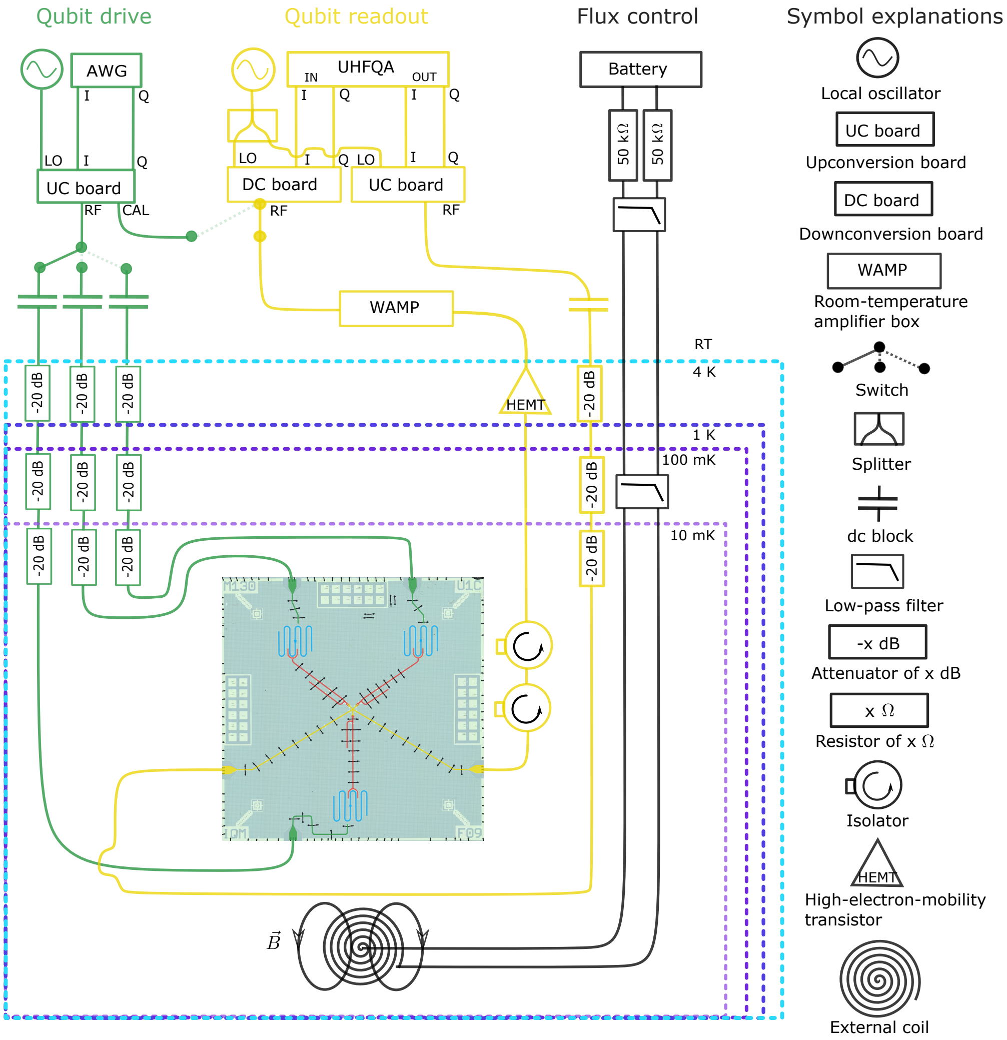

Supplementary Fig. 9: **Experimental setup.** Schematic experimental setup used in the measurements of the unimon qubit devices. Electronics and components related to qubit drive, qubit readout, and flux control are denoted with green, yellow, and black colors, respectively. The dashed rectangles illustrate boundaries between the different temperature stages of the cryostat. Brief explanations of the symbols are provided in the rightmost column of the figure. Note that the qubit readout does not utilize any parametric amplifier.

Supplementary Methods V: Targeted design values for the most relevant parameters of the unimon devices

Supplementary Table **2** presents the targeted design values for the most relevant circuit parameters of the five unimon qubit devices studied in this work.

Supplementary Table 2: **Design values of the characteristic parameters of the five unimon qubits.** The design values listed in the table include the inductance L_l and capacitance C_l per unit length of the center conductor of the qubit, the characteristic impedance Z of the transmission line in the qubit, the coupling capacitance C_g and coupling strength $|g_{01}|/(2\pi)$ between the qubit and its readout resonator, the coupling capacitance C_d between the qubit and its drive line, the dispersive shift $\chi/(2\pi)$ of the qubit, the frequency f_r of the readout resonator, the coupling capacitance C_κ between the readout resonator and the readout transmission line, and the linewidth of the readout resonator $\kappa/(2\pi)$. Note that the coupling strength $|g_{01}|/(2\pi)$ is estimated by a simulation, in which the resonator frequency is tuned on resonance with a linearized unimon circuit in order to measure $|g_{01}|/(2\pi)$ from the frequency separation of the resonances in the formed avoided crossing.

Qubit	L_l (μH/m)	C_l (pF/m)	Z (Ω)	C_g (fF)	$\|g_{01}\|/(2\pi)$ (MHz)	C_d (fF)	$\chi/(2\pi)$ (MHz)	f_r (GHz)	C_κ (fF)	$\kappa/(2\pi)$ (MHz)
**A**	0.83	83	100	10.4	53.2	0.083	0.89	6.0	16.8	0.89
**B**	0.83	83	100	10.4	53.2	0.083	0.58	6.3	12.8	0.58
**C**	0.83	83	100	14.0	69.4	0.083	2.48	5.7	29.6	2.48
**D**	0.83	83	100	14.0	69.4	0.083	1.44	6.0	28.1	1.44
**E**	0.83	83	100	14.0	69.4	0.083	0.94	6.3	21.5	0.94

Supplementary Note I: Two-qubit gates for unimon

In order to implement universal quantum computation, one needs to implement entangling two-qubit gates in addition to single-qubit gates. Here, we theoretically show that methods common for conventional qubits such as transmons to realize two-qubit gates can be utilized for unimons as well.

We focus on cross-resonant implementation of the CNOT gate[11, 12] between two capacitively coupled unimons shown in Supplementary Fig. **10**(a). We drive both qubits coherently with the frequency of the target qubit. The amplitudes and relative phases of the drive pulses are fine-tuned in such a way that the unwanted transition between $|00\rangle$ and $|01\rangle$ states is suppressed. The Hamiltonian of the two-unimon system reads as

$$\hat{H} = \sum_{j=1}^{2} \left[4E_{Cj}\hat{n}_j^2 + \frac{1}{2}E_{Lj}\hat{\varphi}_j^2 + E_{\mathrm{J}j}\cos\left(\hat{\varphi}_j\right) \right] + E_{\mathrm{c}}\hat{n}_1\hat{n}_2, \tag{129}$$

where E_{Cj}, E_{Lj}, and $E_{\mathrm{J}j}$ are the capacitive, inductive, and Josephson energies of the j:th unimon, respectively, E_{c} is the coupling energy, and we take the flux difference through the loops of each unimon to be $\Phi_0/2$. Owing to the small but finite direct coupling $E_{\mathrm{c}} \neq 0$, the eigenstates of this Hamiltonian deviate from the eigenstates of the uncoupled unimons. Consequently, we construct the computational basis $|00\rangle$, $|01\rangle$, $|10\rangle$, and $|11\rangle$ from the eigenstates which have the highest overlap with the respective bare qubit states.

To proceed, we apply a coherent drive pulse to the qubits described by the following operator

$$\hat{H}_{\mathrm{d}} = A\,\mathrm{e}^{-\frac{t^2}{\tau^2}}\sin(\omega_2 t)\left(\hat{n}_1 - a\hat{n}_2\right), \tag{130}$$

where τ is the duration and A is the maximum amplitude of the gate, ω_2 is the angular frequency between states $|10\rangle$ and $|11\rangle$, and the relative drive amplitude between of the first and the second qubit is given by $a = \langle 00|\hat{n}_1|01\rangle/\langle 00|\hat{n}_2|01\rangle$ in order to suppress transition between the states $|00\rangle$ and $|01\rangle$. The maximum amplitude is adjusted to

$$A = \frac{\sqrt{\pi}\hbar}{\tau|\langle 10|\hat{n}_1 - a\hat{n}_2|11\rangle|}. \tag{131}$$

We define the gate as an evolution operator in the rotating frame of the two-unimon system projected onto the computational subspace as

$$\hat{U}_{\mathrm{CNOT}} = e^{i\hat{H}(t_{\mathrm{f}}-t_{\mathrm{i}})/\hbar}\,\mathcal{T}\exp\left\{ -i\int_{t_{\mathrm{i}}}^{t_{\mathrm{f}}} \left[\hat{H} + \hat{H}_{\mathrm{d}}(t)\right]/\hbar\,\mathrm{d}t \right\}, \tag{132}$$

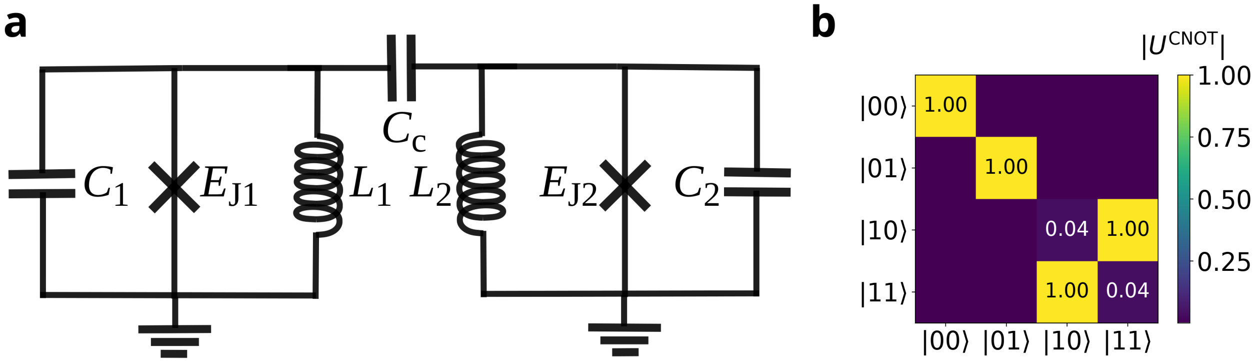

Supplementary Fig. 10: **Two-qubit gates for unimons.** **a**, Lumped-element circuit diagram for two capacitively coupled unimon qubits. **b**, Absolute values of the unitary matrix corresponding to the temporal evolution arising from the cross-resonance CNOT gate as described in the text below. Parameters of the unimons are equal to $E_{C1}/h = 0.318$ GHz, $E_{L1}/h = 24.9$ GHz, $E_{\rm J1}/h = 23.3$ GHz, $E_{C2}/h = 0.297$ GHz, $E_{L1}/h = 25.2$ GHz, $E_{\rm J2}/h = 19.0$ GHz, and $E_{\rm c}/h = 5$ MHz which correspond to the experimental parameters of the unimons A and B (see Table 1 of the main text) and a much weaker coupling energy than that of the capacitive resonator–unimon coupling implemented in our experiments. The characteristic duration of the gate is set to $\tau = 200$ ns. We estimate the fidelity of the CNOT gate as $\frac{1}{16}\left|\mathrm{Tr}\left\{\hat{U}^\dagger_{\rm ideal}\hat{U}_{\rm CNOT}\right\}\right|^2 = 0.996$, where $U_{\rm ideal}$ is the ideal CNOT gate and $U_{\rm CNOT}$ is that implemented with these parameters in the employed rotating frame.

where $t_{\rm i} \sim -3\tau$ and $t_{\rm f} \sim 3\tau$ are the times of beginning and the end of the gate. Note that to highlight the capabilities of two-qubit unimon gates in comparison to the state-of-the-art transmons, we effectively cancel the residual ZZ coupling by the above choice of the rotating frame, justified by the fact that the residual coupling can be suppressed by off-resonant drive fields which are not included in our simulations for the sake of simplicity. As shown in Supplementary Fig. **10**(b), the temporal evolution according to the above two-unimon Hamiltonian augmented by the drive Hamiltonian leads to a high-fidelity CNOT gate. Thus, we have theoretically demonstrated that two-qubit gates are implementable in a multiqubit unimon processor. In the future, we aim to study also tunable couplers for unimons and to focus on the most promising approaches for two-qubit unimon gates.

Supplementary Note II: Dispersive shift and critical photon number in qubit readout

In the usual dispersive qubit readout, its speed and fidelity are largely determined by the dispersive shift $\chi/(2\pi)$ and the linewidth of the readout resonator $\kappa/(2\pi)$[13,14]. This can be understood by considering a semi-classical model, in which the readout resonator is treated as a classical damped and driven oscillator, the frequency of which depends on the state of the qubit due to the dispersive shift. In such a model, the state-dependent complex output response signal $s_{\mathrm{g/e}}(t)$ can be derived to be[14]

$$s_{\mathrm{g/e}}(t) \propto \frac{\kappa}{\kappa + 2\mathrm{i}\Delta_{\mathrm{g/e}}} \left[1 - \mathrm{e}^{-t(\mathrm{i}\Delta_{\mathrm{g/e}}+\kappa/2)}\right], \tag{133}$$

where $\Delta_{\mathrm{g/e}} = \omega_{\mathrm{d}} - \omega_{\mathrm{r}} \pm \chi$ with ω_{d} being the frequency of the resonator drive. Based on this model, the dispersive shift $\chi/(2\pi)$ determines the initial increase rate of the readout contrast $s_{\mathrm{g}}(t) - s_{\mathrm{e}}(t)$, whereas the linewidth $\kappa/(2\pi)$ accounts for the initial increase rate of the overall signal. Furthermore, an optimal readout contrast is achieved by designing the coupled resonator-qubit system such that $\chi = \kappa/2$. Thus, a large dispersive shift together with a correspondingly large resonator linewidth are required for fast and high-fidelity single-shot readout. However, the increased resonator linewidth also brings about an increased Purcell decay according to Eq. (128), the effect of which can be reduced by an appropriately designed Purcell filter[15].

For the unimon that has anharmonicity exceeding that of the transmon but lying well below the qubit angular frequency, the expression of the dispersive shift in Eq. (112) can be further approximated as

$$\chi \approx \frac{|g_{01}|^2\alpha}{(\omega_{01} - \omega_{\mathrm{r}})(\omega_{01} - \omega_{\mathrm{r}} + \alpha)}, \tag{134}$$

where we have invoked the assumption that $g_{12}/g_{01} = n_{12}/n_{01} \approx \sqrt{2}$, which holds within 5.2% (11.0%) across all values of the applied flux difference for the parameters of qubit B (qubit A) as illustrated in Supplementary Fig. **11**(a). Note that the obtained approximation for the dispersive shift is similar to the case of the transmon qubits[10]. In Supplementary Fig. **11**(b), we illustrate the dispersive shift based on the above approximation as a function of the qubit anharmonicity by assuming that only the anharmonicity is changed while the circuit parameters are chosen such that the unimon-resonator coupling g_{01} and the unimon-resonator detuning $\omega_{01} - \omega_{\mathrm{r}}$ are fixed to the values measured for qubit B at $\Phi_{\mathrm{diff}}/\Phi_0 = 0.5$. From the figure, we observe that the dispersive shift is drastically enhanced with increasing anharmonicity, which suggests that the high anharmonicity of the unimon may provide an advantage over transmon qubits with a low anharmonicity in implementing fast and high-fidelity single-shot readout. On the other hand, an increase in the qubit anharmonicity enables one to reach an equally large dispersive shift with a smaller coupling

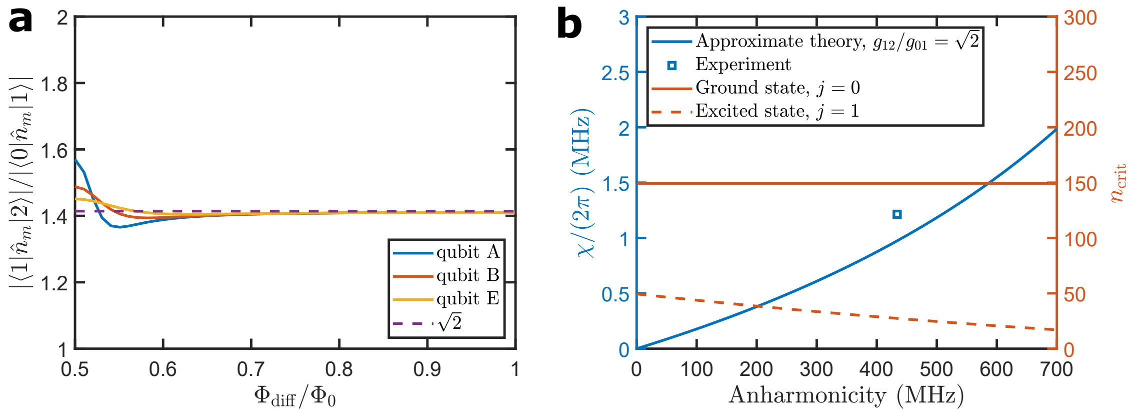

Supplementary Fig. 11: **Dispersive shift and critical photon number in qubit readout. a**, Ratio of the charge matrix elements $n_{12} = \langle 1|\hat{n}_m|2\rangle$ and $n_{01} = \langle 0|\hat{n}_m|1\rangle$ as a function of the external flux difference for the parameters of qubits A, B, and E (see Table 1 of the main text). The ratio n_{12}/n_{01} differs from that of a harmonic oscillator ($n_{12}/n_{01} = \sqrt{2}$) by at most 5.2% (11.0%) in the case of qubit B (qubit A) that has a sweet-spot anharmonicity of $\alpha/(2\pi) = +434$ MHz ($\alpha/(2\pi) = +744$ MHz). **b**, Dispersive shift $\chi/(2\pi)$ (blue colour) and critical photon number $n_{\rm crit}$ (orange colour) as functions of the qubit anharmonicity for a coupling strength g_{01} and qubit-resonator detuning $\omega_{01} - \omega_r$ set to the measured values of qubit B, i.e., $g_{01}/(2\pi) = 70.0$ MHz and $(\omega_{01} - \omega_{\rm r})/(2\pi) = -1.71$ GHz. The solid blue line shows the dispersive shift based on the approximate expression in Eq. (134), whereas the blue rectangle shows the experimentally measured dispersive shift of qubit B. The solid and dashed orange lines show the critical photon number computed using Eq. (135) for the ground state ($j = 0$) and for the excited state ($j = 1$), respectively.

rate g_{01} or a larger detuning $\omega_{01} - \omega_{\rm r}$, which helps to reduce the relaxation rate due to the Purcell effect while keeping the speed and fidelity of the readout essentially unchanged.

However, the increased anharmonicity may slightly lower the critical photon number of the readout resonator coupled to the unimon. Under the approximation $g_{12}/g_{01} = n_{12}/n_{01} \approx \sqrt{2}$, the critical photon number can be approximated using a similar expression as in the case of transmon qubits[4]

$$n_{\rm crit} = \frac{1}{2j+1}\left(\frac{|\omega_{01} - \omega_{\rm r} + j\alpha|^2}{4g_{01}^2} - j\right), \tag{135}$$

where $j = 0$ and $j = 1$ correspond to the qubit being in either the ground state or the excited state. As illustrated in Supplementary Fig. **11**(b), the critical photon number corresponding to $j = 1$ decreases with increasing anharmonicity, but the relative decrease of the critical photon number is

much smaller than the relative increase of the dispersive shift.

An enhanced dispersive shift resulting from a high anharmonicity is not the only advantage that the unimon may have in comparison to transmon qubits when it comes to the qubit readout. As a consequence of the cosine-shaped Josephson potential of the transmon, the transmon has unbound states above the cosine potential, to which the transmon may be excited during a high-amplitude readout pulse required for fast readout[16,17]. Interestingly, the readout pulse may cause resonances with the unbound states already at the few-photon level degrading the fidelity and quantum-non-demolition properties of the readout with transmon qubits[17]. Owing to the confinement of the potential in the unimon, all eigenstates of the unimon are bound, implying that the unimon should not suffer from the undesired effects arising from resonances with unbound states. Naturally, this argument also applies to fluxonium qubits that also include an inductor shunting the Josephson junction. However, the above analysis on the dispersive shift and critical photon number does not necessarily apply to fluxonium qubits, for which the anharmonicity is on par or exceeds the qubit transition frequency.

Supplementary References

1. Deaver Jr, B. S. & Fairbank, W. M. Experimental evidence for quantized flux in superconducting cylinders. *Physical Review Letters* **7**, 43 (1961).

2. Doll, R. & Näbauer, M. Experimental proof of magnetic flux quantization in a superconducting ring. *Physical Review Letters* **7**, 51 (1961).

3. Bourassa, J., Beaudoin, F., Gambetta, J. M. & Blais, A. Josephson-junction-embedded transmission-line resonators: From kerr medium to in-line transmon. *Physical Review A* **86**, 013814 (2012).

4. Blais, A., Grimsmo, A. L., Girvin, S. & Wallraff, A. Circuit quantum electrodynamics. *Reviews of Modern Physics* **93**, 025005 (2021).

5. Schoelkopf, R., Clerk, A., Girvin, S., Lehnert, K. & Devoret, M. Qubits as spectrometers of quantum noise. In *Quantum noise in mesoscopic physics*, 175–203 (Springer, 2003).

6. Bylander, J. *et al.* Noise spectroscopy through dynamical decoupling with a superconducting flux qubit. *Nature Physics* **7**, 565–570 (2011).

7. Hazard, T. *et al.* Nanowire superinductance fluxonium qubit. *Physical review letters* **122**, 010504 (2019).

8. Zhang, H. *et al.* Universal fast-flux control of a coherent, low-frequency qubit. *Physical Review X* **11**, 011010 (2021).

9. Vool, U. & Devoret, M. Introduction to quantum electromagnetic circuits. *International Journal of Circuit Theory and Applications* **45**, 897–934 (2017).

10. Koch, J. *et al.* Charge-insensitive qubit design derived from the cooper pair box. *Physical Review A* **76**, 042319 (2007).

11. Paraoanu, G. S. Microwave-induced coupling of superconducting qubits. *Phys. Rev. B* **74**, 140504 (2006). URL `https://link.aps.org/doi/10.1103/PhysRevB.74.140504`.

12. Rigetti, C. & Devoret, M. Fully microwave-tunable universal gates in superconducting qubits with linear couplings and fixed transition frequencies. *Phys. Rev. B* **81**, 134507 (2010). URL `https://link.aps.org/doi/10.1103/PhysRevB.81.134507`.

13. Ikonen, J. *et al.* Qubit measurement by multichannel driving. *Physical review letters* **122**, 080503 (2019).

14. Heinsoo, J. *Digital quantum computation with superconducting qubits*. Ph.D. thesis, ETH Zurich (2019).

15. Reed, M. D. *et al.* Fast reset and suppressing spontaneous emission of a superconducting qubit. *Applied Physics Letters* **96**, 203110 (2010).

16. Lescanne, R. *et al.* Escape of a driven quantum josephson circuit into unconfined states. *Physical Review Applied* **11**, 014030 (2019).

17. Shillito, R. *et al.* Dynamics of transmon ionization. *arXiv preprint arXiv:2203.11235* (2022).
